# Supplementary material for: Curriculum Design and Scholarship for New Educators: A Professional Development Workshop for Medical Students
Source: MedEdPORTAL. 2021 Apr 26;17:11130. doi: 10.15766/mep_2374-8265.11130 (PMC8071841; doi:10.15766/mep_2374-8265.11130)
Supplement: Supplementary file 1 — Workshop Agenda.docxPresentation.pptxWorksheet.docxFacilitator Notes.docxWorkshop Survey.docx [file mep_2374-8265.11130-s001.zip › A. Workshop Agenda.docx]

**Curriculum Design and Scholarship for New Educators:
A Professional Development Workshop for Medical Students**

**3-hr Workshop**

**Educational Objectives**

By the end of this activity, learners will be able to:

1. Apply principles of the generally accepted six-step approach for curriculum development for health professions education
2. Describe characteristics of adult learners
3. Develop SMART Learning Objectives
4. Describe educational strategies considered to promote active learning
5. Identify factors influencing the implementation of health professions curricula
6. Differentiate assessment and evaluation
7. Describe foundations of educational scholarship

**Setting**:

1. Participants in small breakout groups (breakout rooms in ZOOM) of 4-6 students each.
2. The content is developed in several steps with the participants in breakout groups guided by a worksheet or Google docs. Each group will elect a scribe to record work on charts for reporting out through screen sharing.
3. 1-2 groups report out after each section, other groups comment
   1. Groups alternate in reporting/commenting
      1. Two (2) groups report out to the plenary completion of Steps 1-2; other groups comment
      2. One – two (1-2) groups report out to the plenary completion of Step 3; other groups comment
      3. One – two (1-2) groups report out to the plenary completion of Steps 4-5; other groups comment
      4. One – two (1-2) groups report out to the plenary completion of Step 6; other groups comment
      5. Individual participants reflect on and complete Steps 7-8; facilitators answer individual questions after session closes.
4. Optional non-science approachable topics most are familiar with:
   1. Medical Spanish
   2. Disaster Preparedness
   3. Maternal and Fetal Health
   4. LGBTQ+ Health
   5. Pain Management
   6. Addiction (Opioid) Problem
   7. Wellness and Resilience

**Curriculum Design and Scholarship for New Educators:
A Professional Development Workshop for Students**

| Format | Timeframe | Facilitator Guide Section | Slides | Who |
| --- | --- | --- | --- | --- |
| Large Group | 10 min | **Welcome & Introduction of Facilitators**  Introduction of Objectives  Introduction of Workshop Ground Rules | 1-3 | All Facilitators |
| Large Group | 5 min | **Students as active Collaborators and Leaders in Medical Education**  Student-centered overview of medical education professional development and the growing role of students as active collaborators and leaders in medical education. | 4-8 | One Facilitator |
| Large Group | 15 min | **Principles of Program Development**  Overview of Curriculum Design and Program Development following Kern’s Six-Step Cycle | 9-15 | 1-2 Facilitators |
| Small group - large group report out | 20 min | **Curriculum Design: Kern’s Six-Steps 1-2**   - Using a given topic or choosing your own, apply steps 1-2 of Kern’s Six-Steps Cycle with peers - Complete worksheet - Two groups report out | 16-22 | 1-2 Facilitators lead; all facilitate groups |
| Large Group didactics.  Small group - large group report out | 25 min | **Curriculum Design: Kern’s Six-Steps 3**   - Goals, and SMART objectives - Using a given topic, apply Step 3 of Kern’s Six-Steps Cycle with peers - Complete worksheet - Two groups report out | 23-28 | One Facilitator leads; all facilitate groups |
| **10 min BREAK** | | | |  |
| Large Group didactics.  Small group - large group report out | 25 min | **Curriculum Design: Kern’s Six-Steps 4-5**  Educational Strategies Overview  Implementation Overview   - Complete worksheet - Two groups report out | 29-34 | One Facilitator leads; all facilitate groups |
| Large Group didactics.  Small group - large group report out | 25 min | **Curriculum Design: Kern’s Six-Step 6**  Assessment   - Using a given problem, apply Step 6 of Kern’s Six-Steps Cycle with your peers - Complete worksheet - Two groups report out | 35-40 | One Facilitator leads; all facilitate groups |
| Large Group didactics.  Individual reflection and Q & A with individual facilitators | 40 min | **Scholarship Challenge**   - Individuals reflect on scholarship initiation - Facilitators answer individual Q & A - Wrap-up/Revisiting | 41-45 | All Facilitators |
