# Supplementary material for: Curriculum Design and Scholarship for New Educators: A Professional Development Workshop for Medical Students
Source: MedEdPORTAL. 2021 Apr 26;17:11130. doi: 10.15766/mep_2374-8265.11130 (PMC8071841; doi:10.15766/mep_2374-8265.11130)
Supplement: Supplementary file 1 — Workshop Agenda.docxPresentation.pptxWorksheet.docxFacilitator Notes.docxWorkshop Survey.docx [file mep_2374-8265.11130-s001.zip › B. Presentation.pptx]

## Slide 1
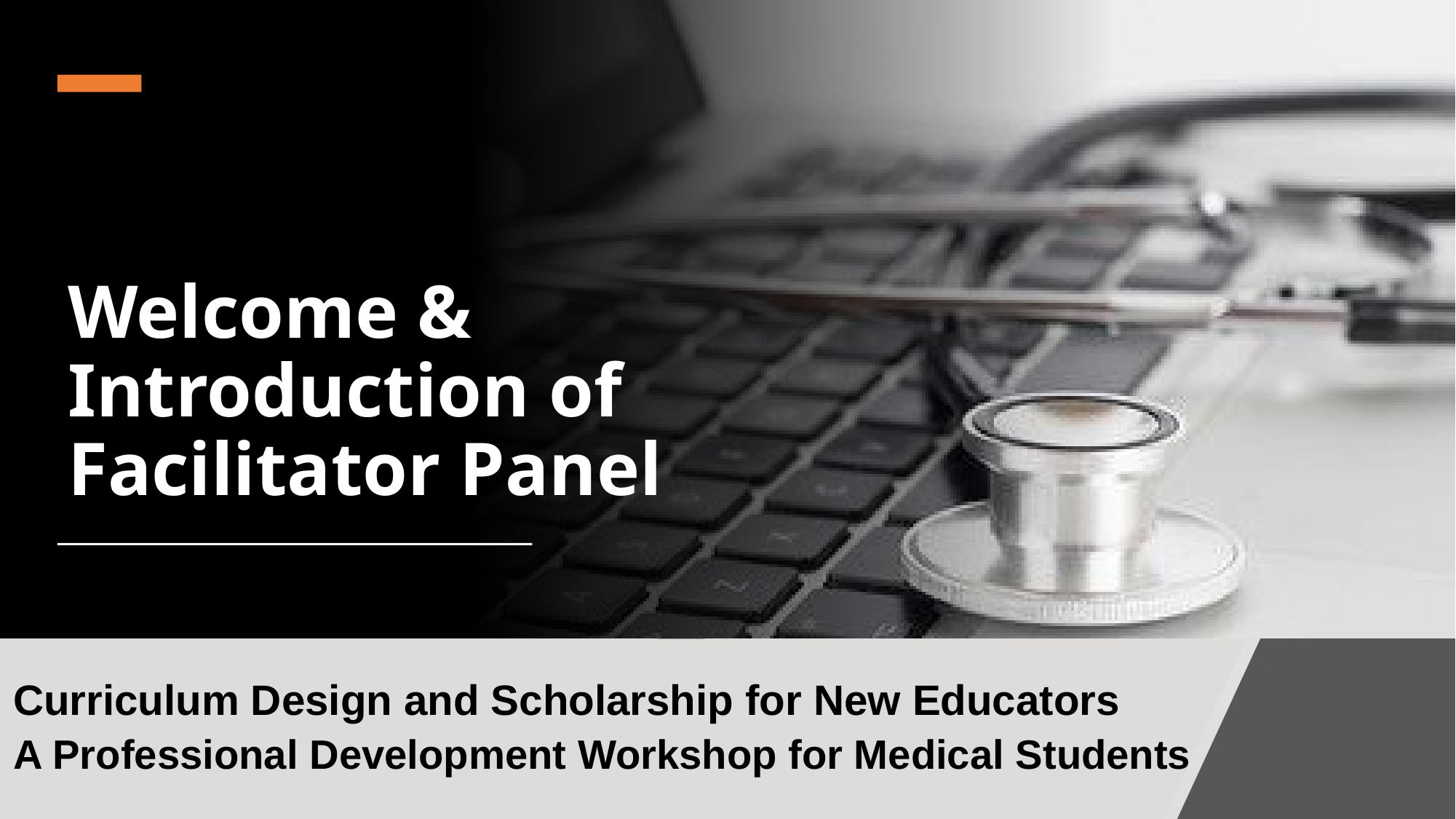

# Welcome & Introduction of Facilitator Panel
Curriculum Design and Scholarship for New Educators
A Professional Development Workshop for Medical Students

## Slide 2
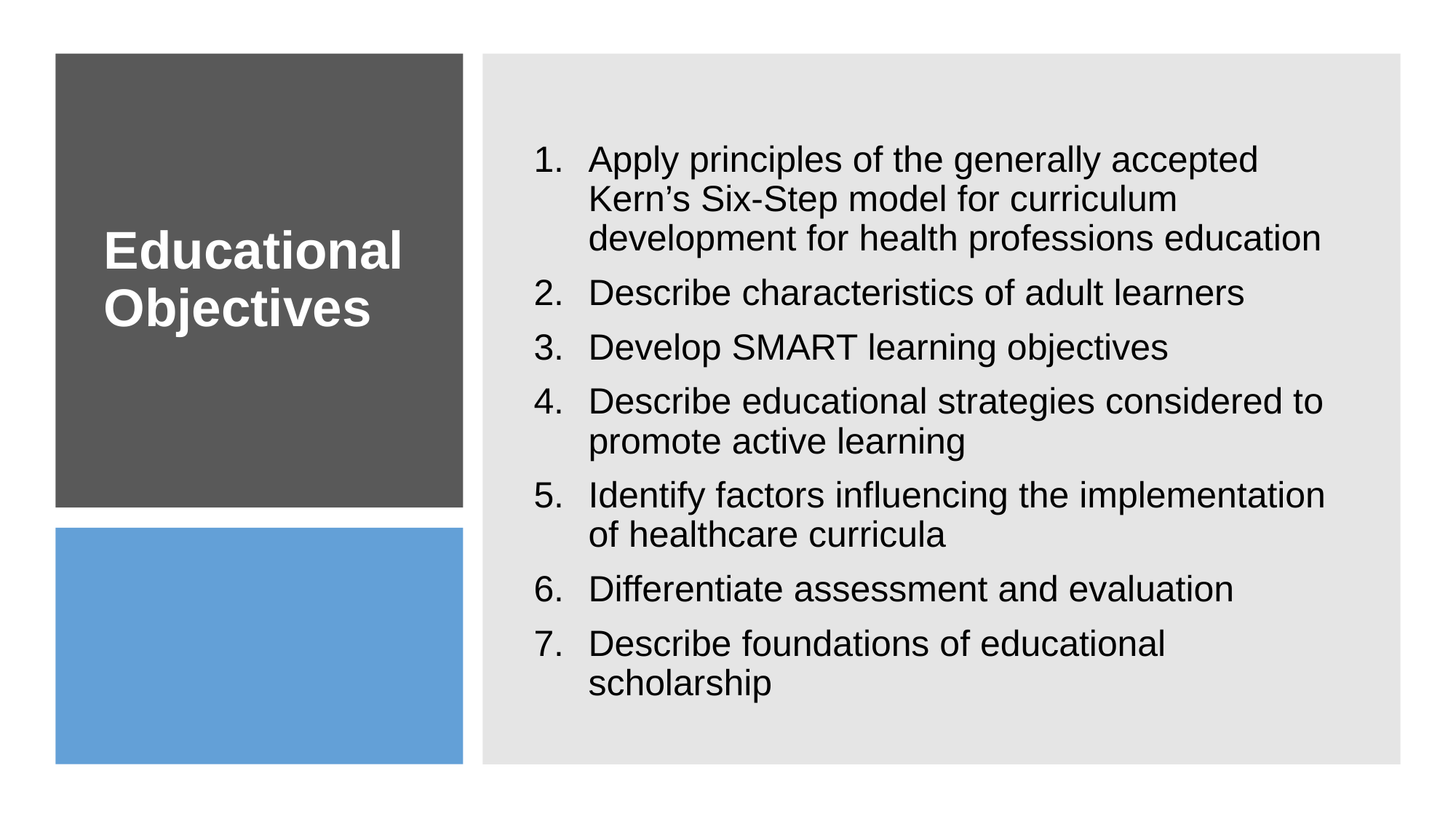

Apply principles of the generally accepted Kern’s Six-Step model for curriculum development for health professions education
Describe characteristics of adult learners
Develop SMART learning objectives
Describe educational strategies considered to promote active learning
Identify factors influencing the implementation of healthcare curricula
Differentiate assessment and evaluation
Describe foundations of educational scholarship
# Educational Objectives

## Slide 3
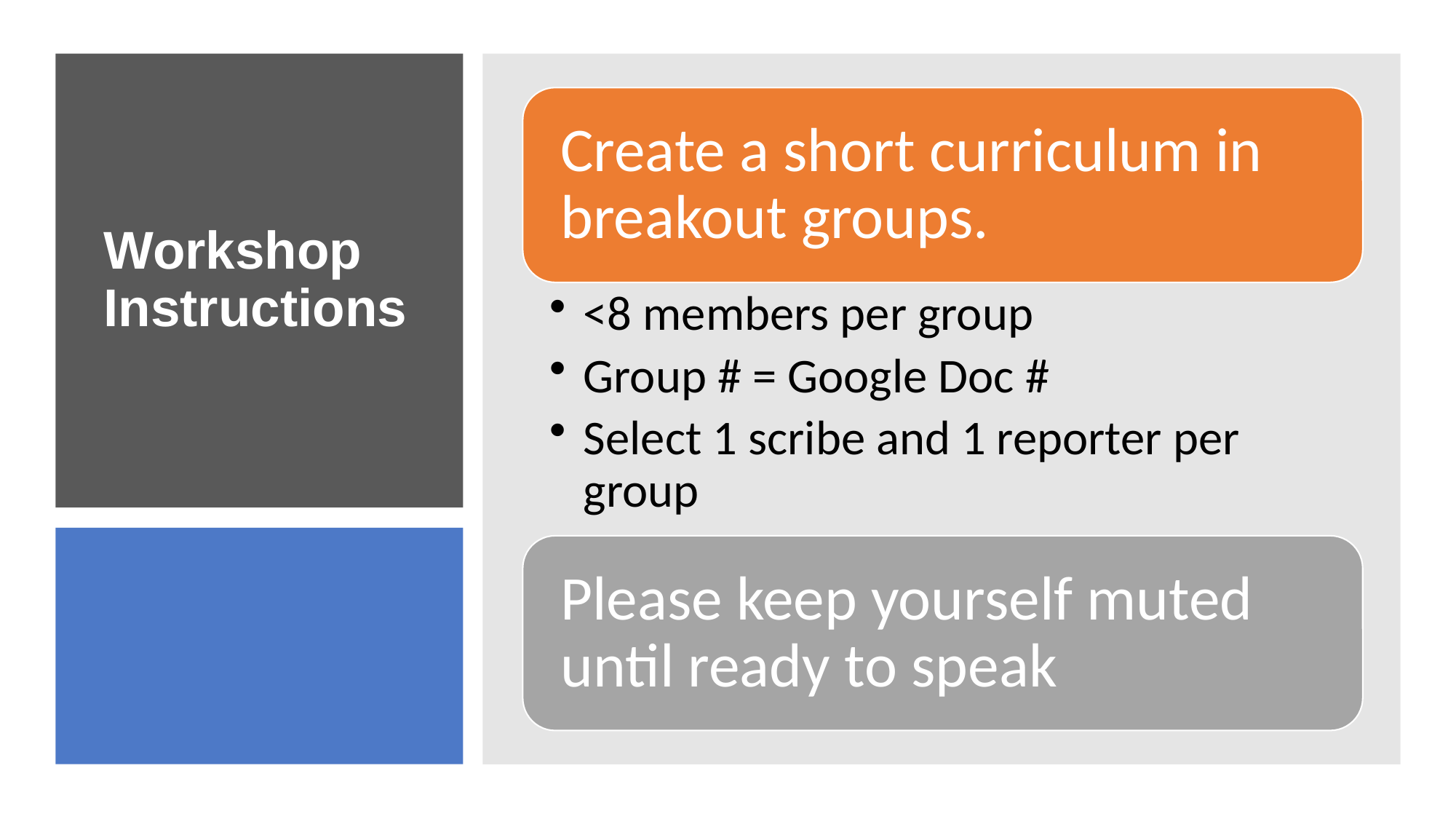

# Workshop Instructions

## Slide 4
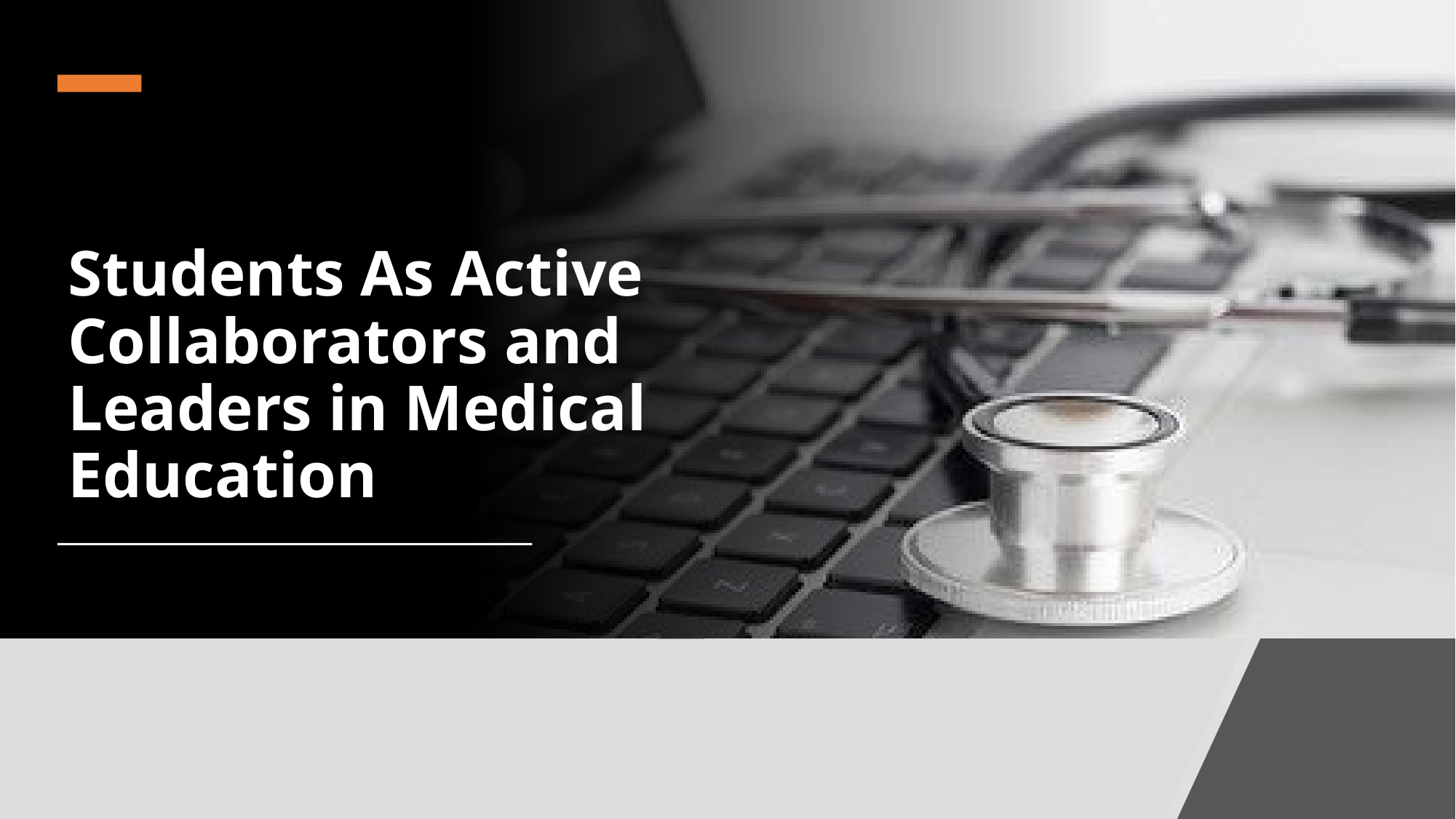

# Students As Active Collaborators and Leaders in Medical Education

## Slide 5
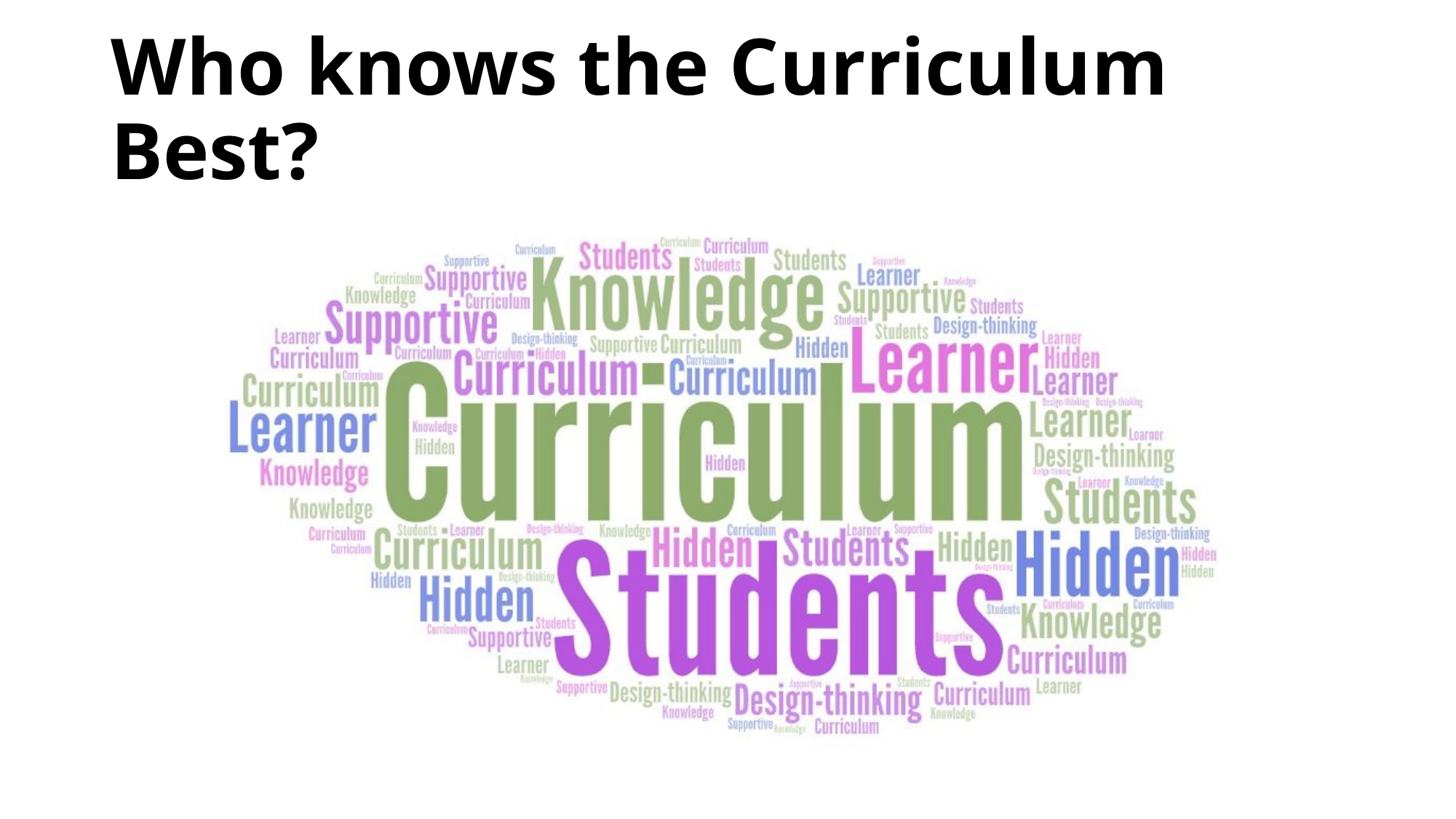

# Who knows the Curriculum Best?

## Slide 6
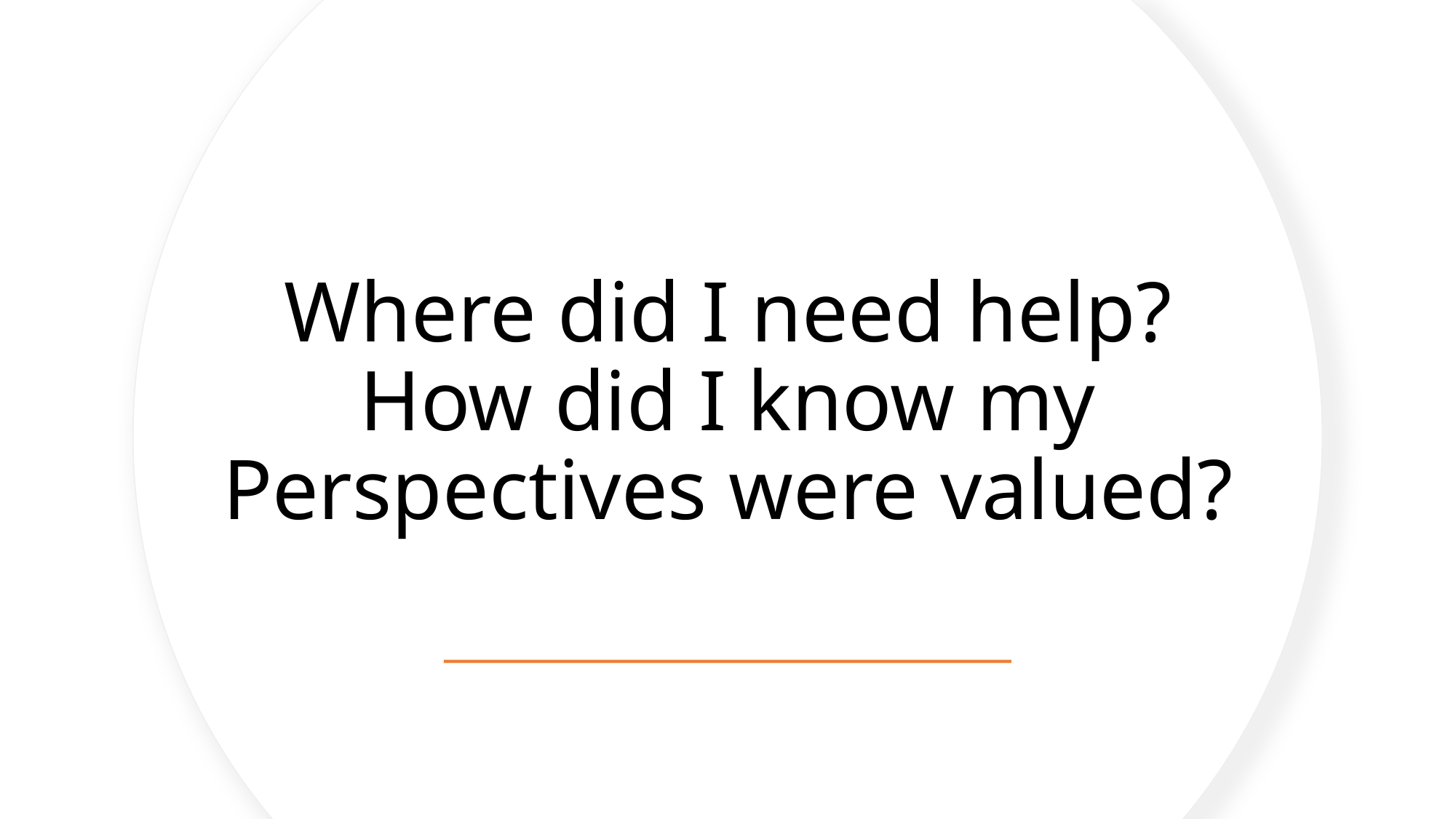

# Where did I need help? How did I know my Perspectives were valued?

## Slide 7
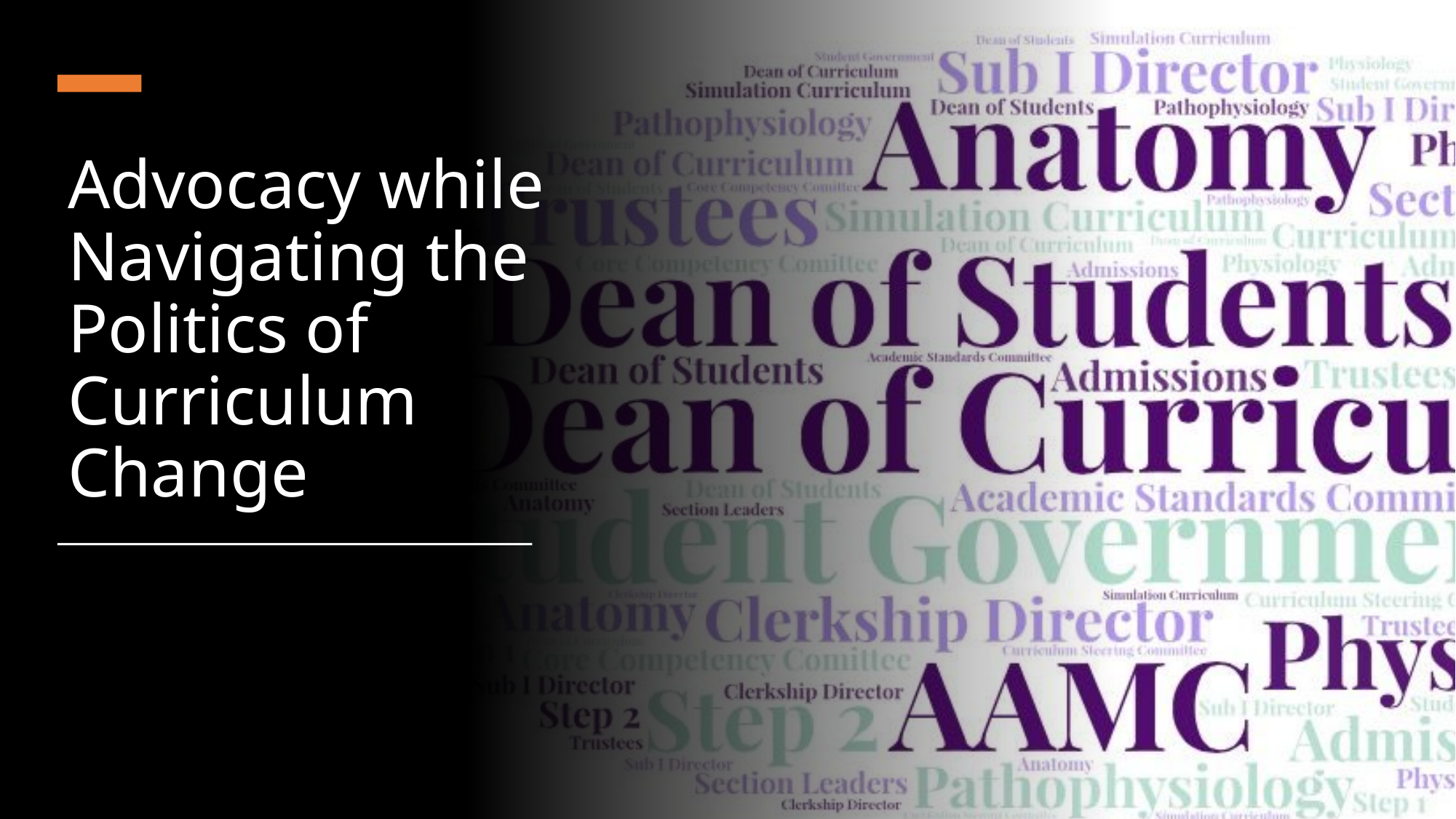

# Advocacy while Navigating the Politics of Curriculum Change

## Slide 8
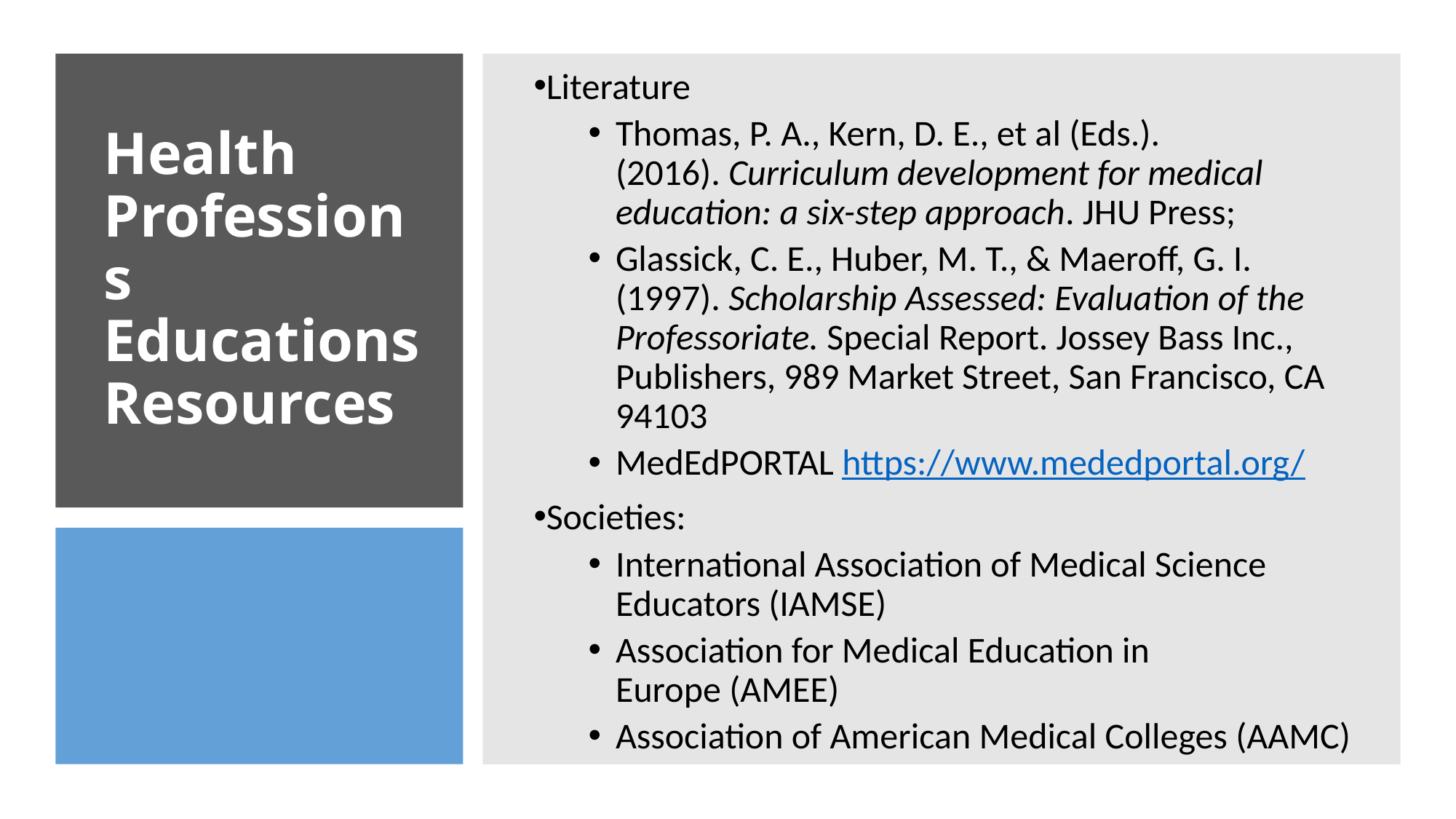

Literature
Thomas, P. A., Kern, D. E., et al (Eds.). (2016). Curriculum development for medical education: a six-step approach. JHU Press;
Glassick, C. E., Huber, M. T., & Maeroff, G. I. (1997). Scholarship Assessed: Evaluation of the Professoriate. Special Report. Jossey Bass Inc., Publishers, 989 Market Street, San Francisco, CA 94103
MedEdPORTAL https://www.mededportal.org/
Societies:
International Association of Medical Science Educators (IAMSE)
Association for Medical Education in Europe (AMEE)
Association of American Medical Colleges (AAMC)
# Health Professions Educations Resources

## Slide 9
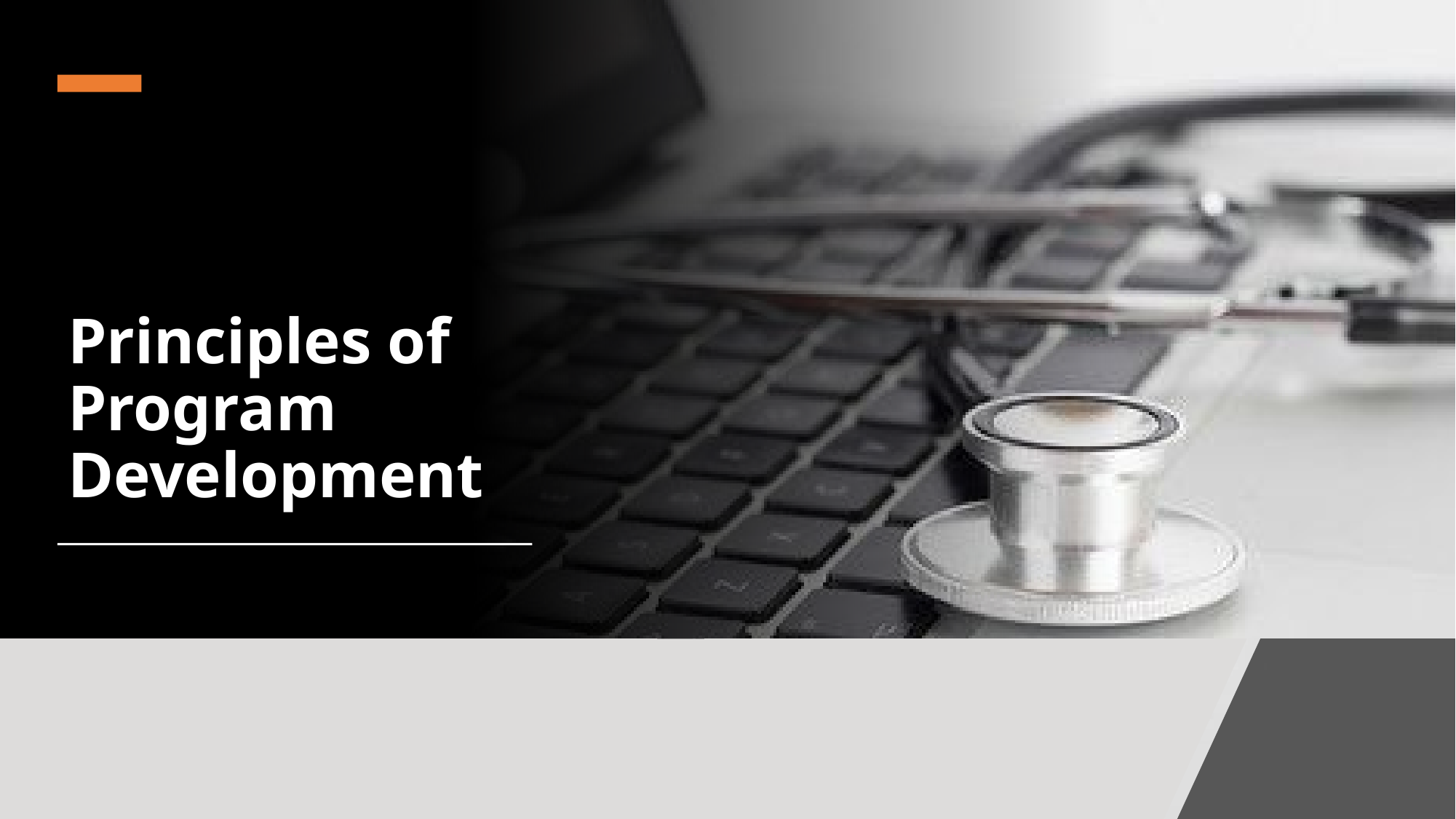

# Principles of Program Development

## Slide 10
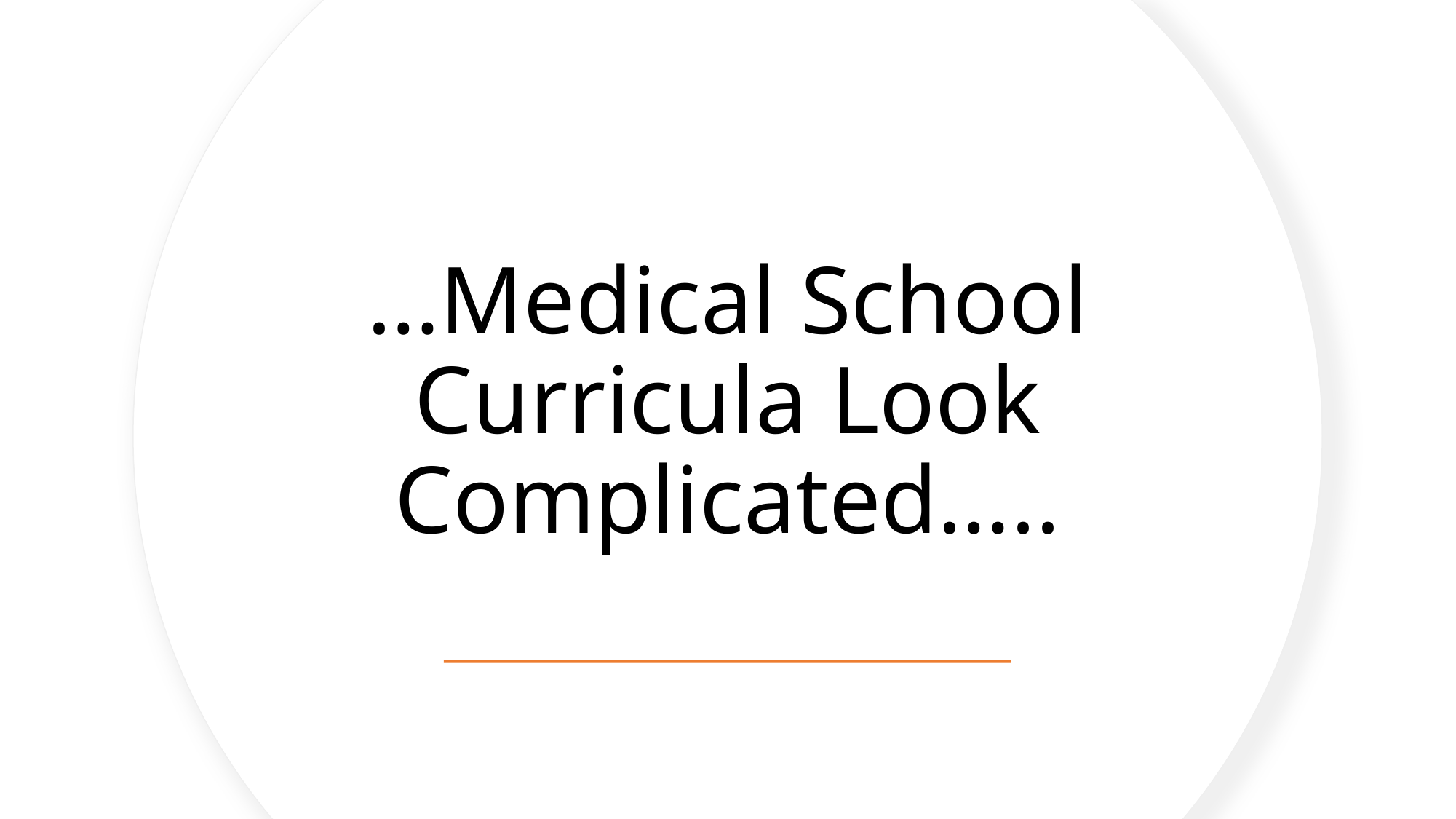

# …Medical School Curricula Look Complicated…..

## Slide 11
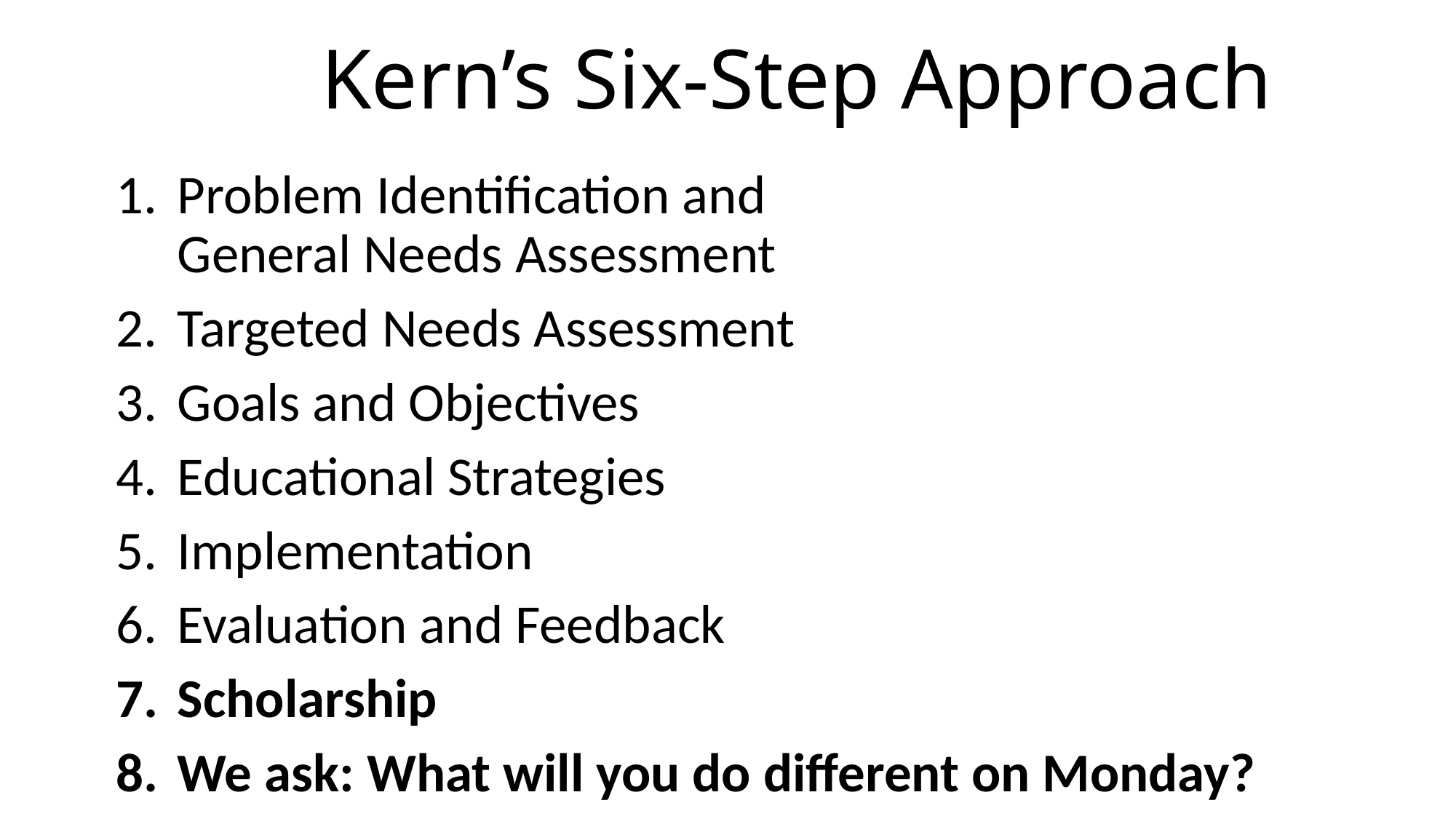

# Kern’s Six-Step Approach
Problem Identification and
General Needs Assessment
Targeted Needs Assessment
Goals and Objectives
Educational Strategies
Implementation
Evaluation and Feedback
Scholarship
We ask: What will you do different on Monday?

## Slide 12
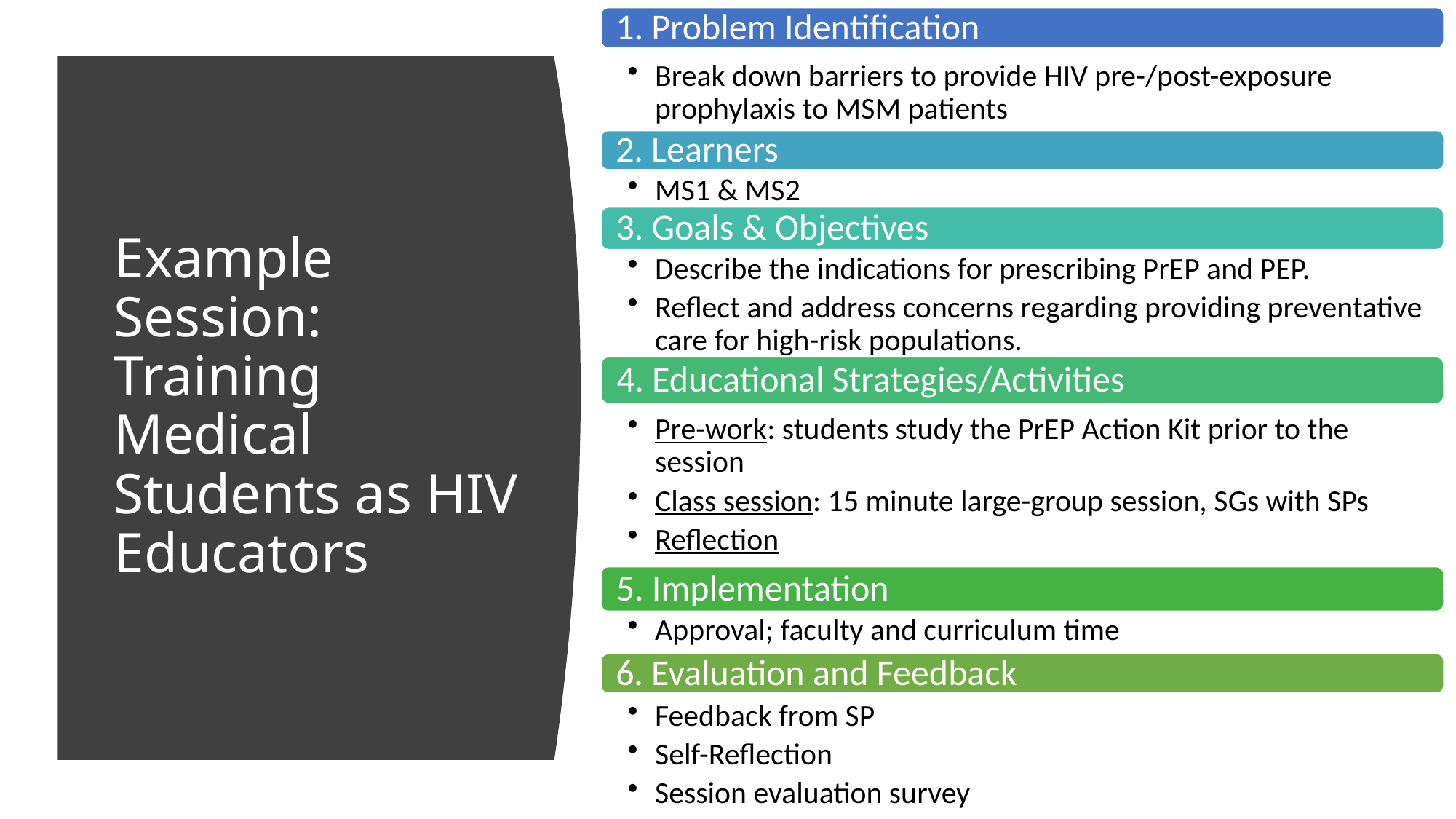

# Example Session: Training Medical Students as HIV Educators

## Slide 13
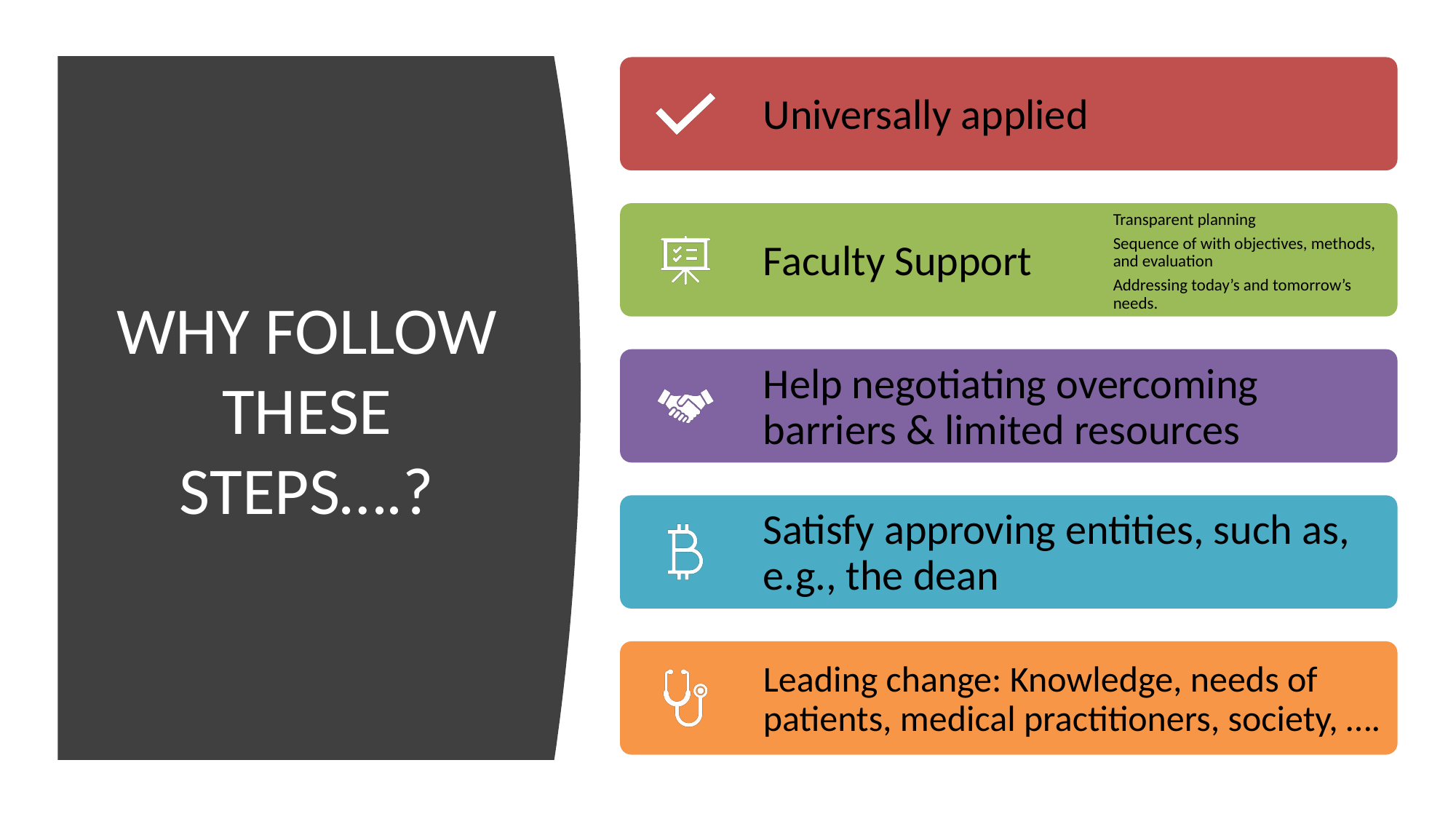

# WHY FOLLOW THESE STEPS….?

## Slide 14
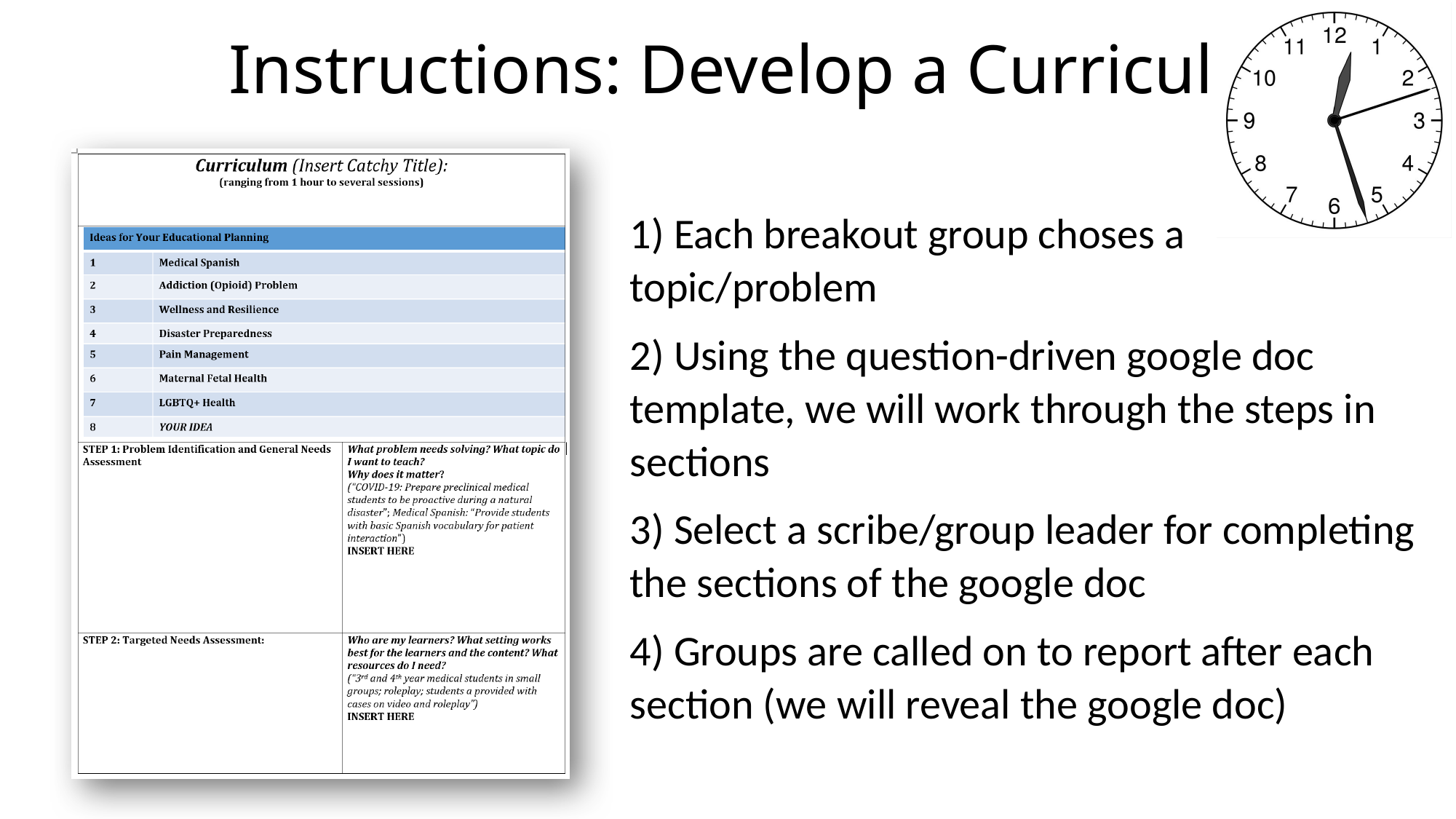

# Instructions: Develop a Curriculum
1) Each breakout group choses a topic/problem
2) Using the question-driven google doc template, we will work through the steps in sections
3) Select a scribe/group leader for completing the sections of the google doc
4) Groups are called on to report after each section (we will reveal the google doc)

## Slide 15
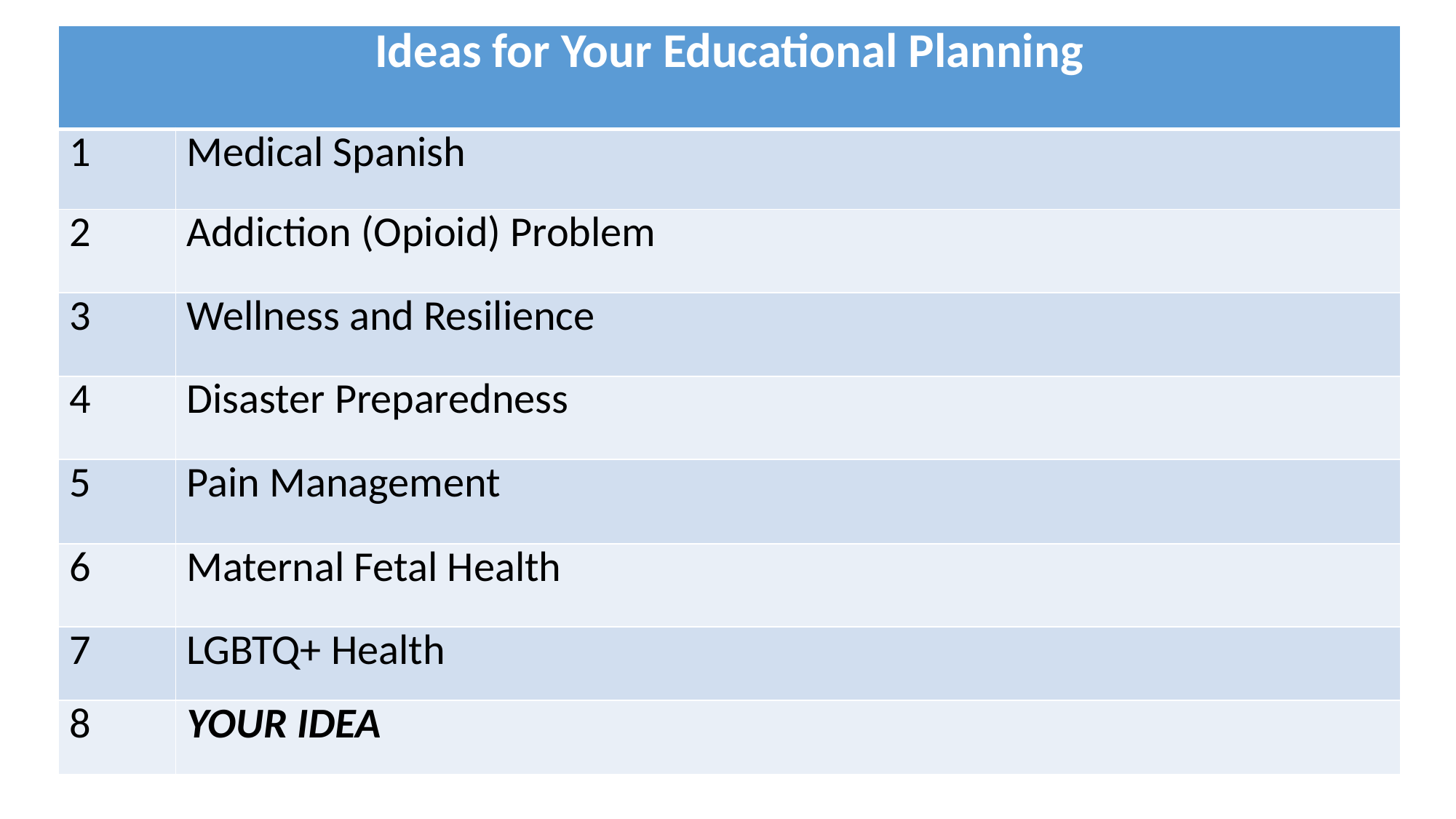

| Ideas for Your Educational Planning | |
| --- | --- |
| 1 | Medical Spanish |
| 2 | Addiction (Opioid) Problem |
| 3 | Wellness and Resilience |
| 4 | Disaster Preparedness |
| 5 | Pain Management |
| 6 | Maternal Fetal Health |
| 7 | LGBTQ+ Health |
| 8 | YOUR IDEA |

## Slide 16
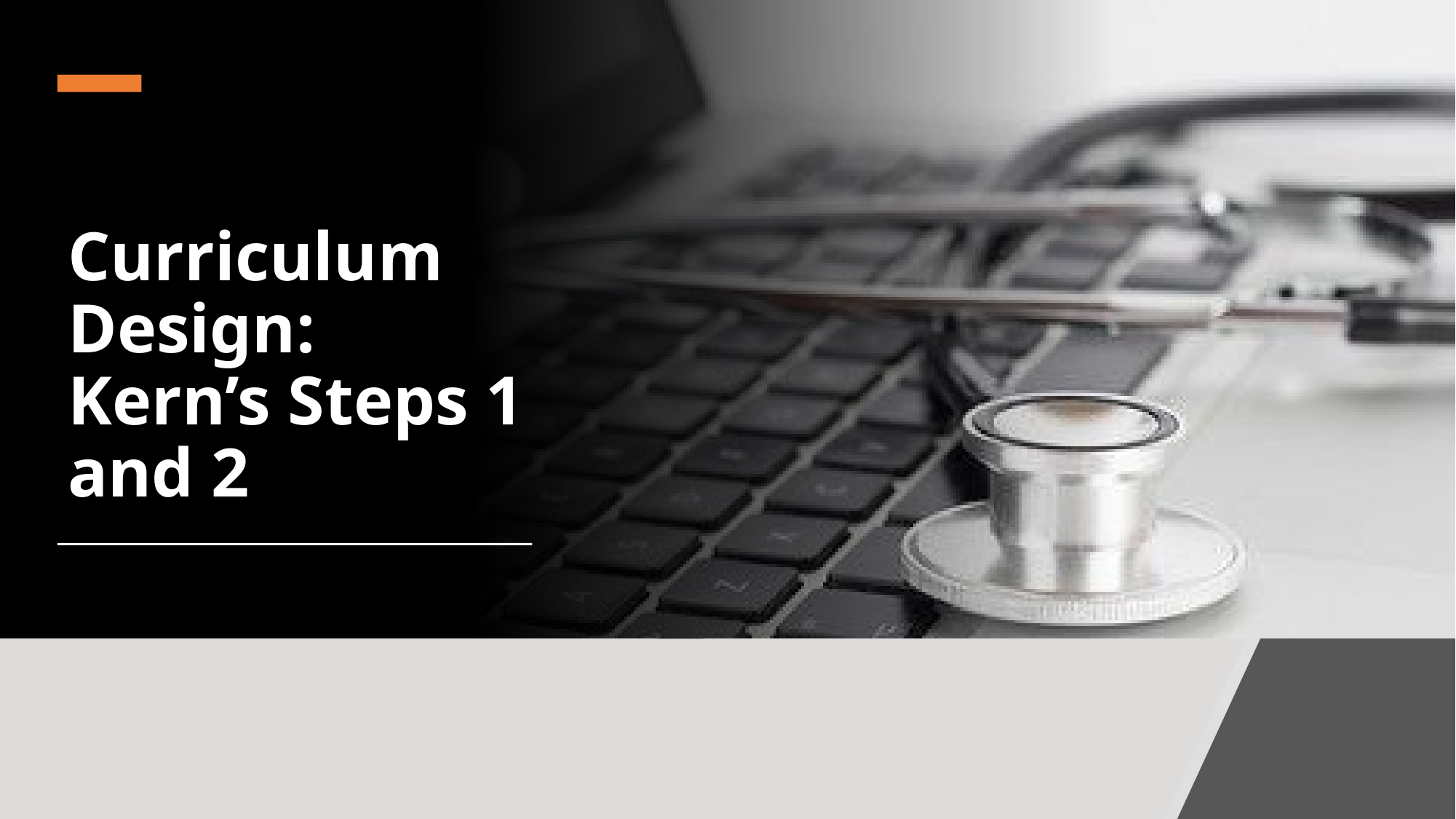

# Curriculum Design: Kern’s Steps 1 and 2

## Slide 17
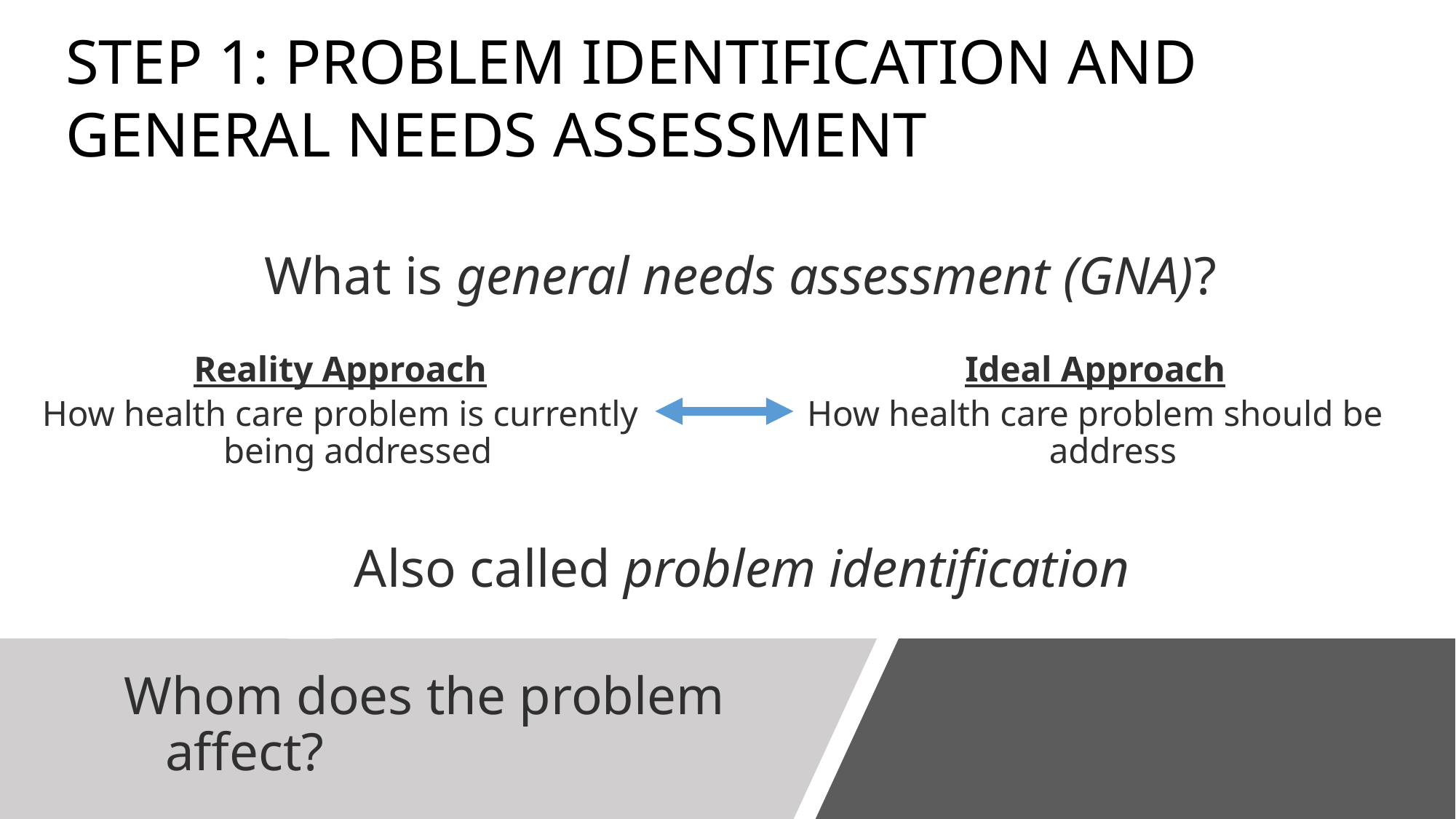

STEP 1: PROBLEM IDENTIFICATION AND GENERAL NEEDS ASSESSMENT
What is general needs assessment (GNA)?
Ideal Approach
How health care problem should be address
Reality Approach
How health care problem is currently being addressed
Also called problem identification
# Whom does the problem affect?

## Slide 18
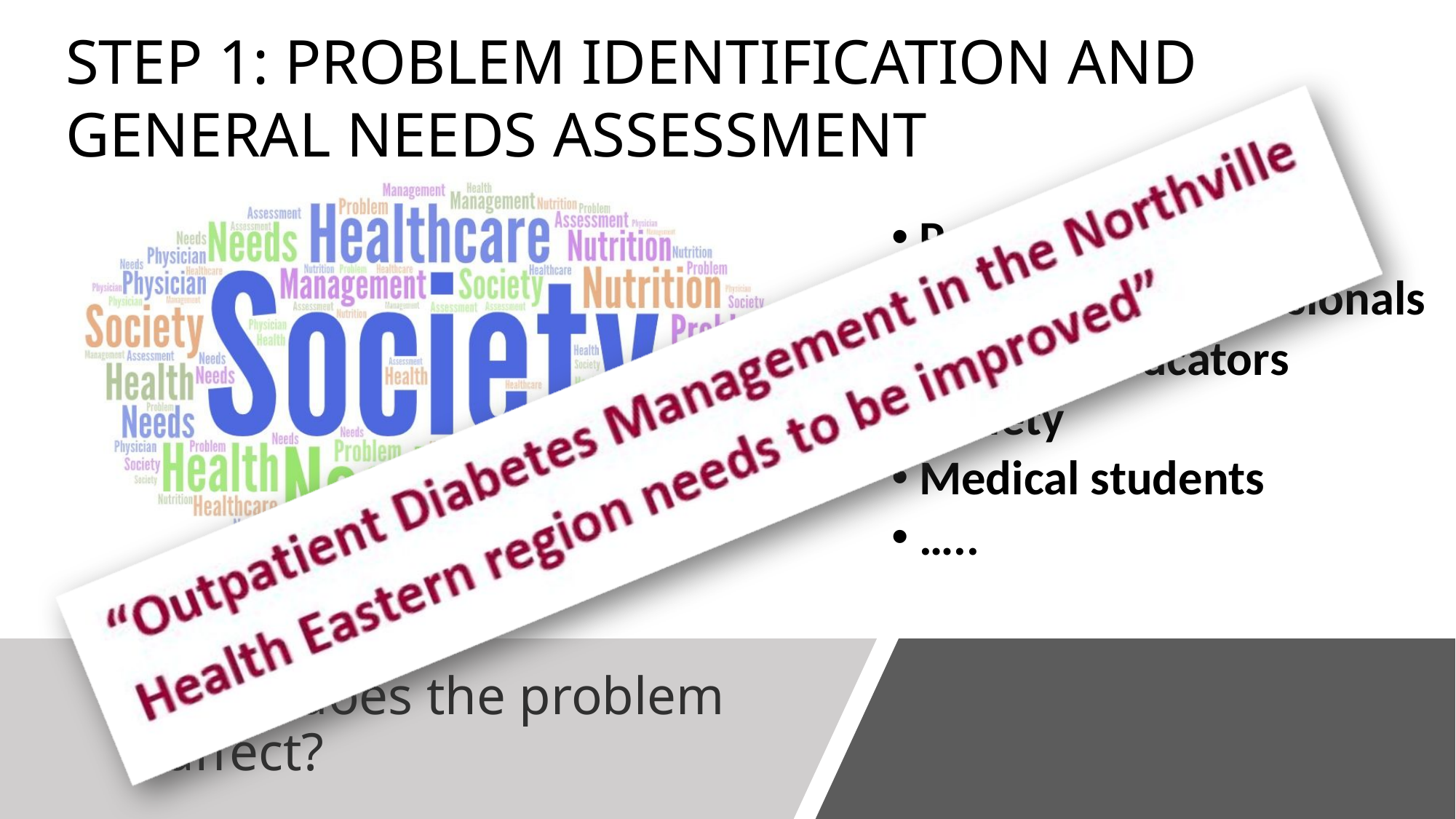

STEP 1: PROBLEM IDENTIFICATION AND GENERAL NEEDS ASSESSMENT
Patients
Health care professionals
Medical educators
Society
Medical students
…..
# Whom does the problem affect?

## Slide 19
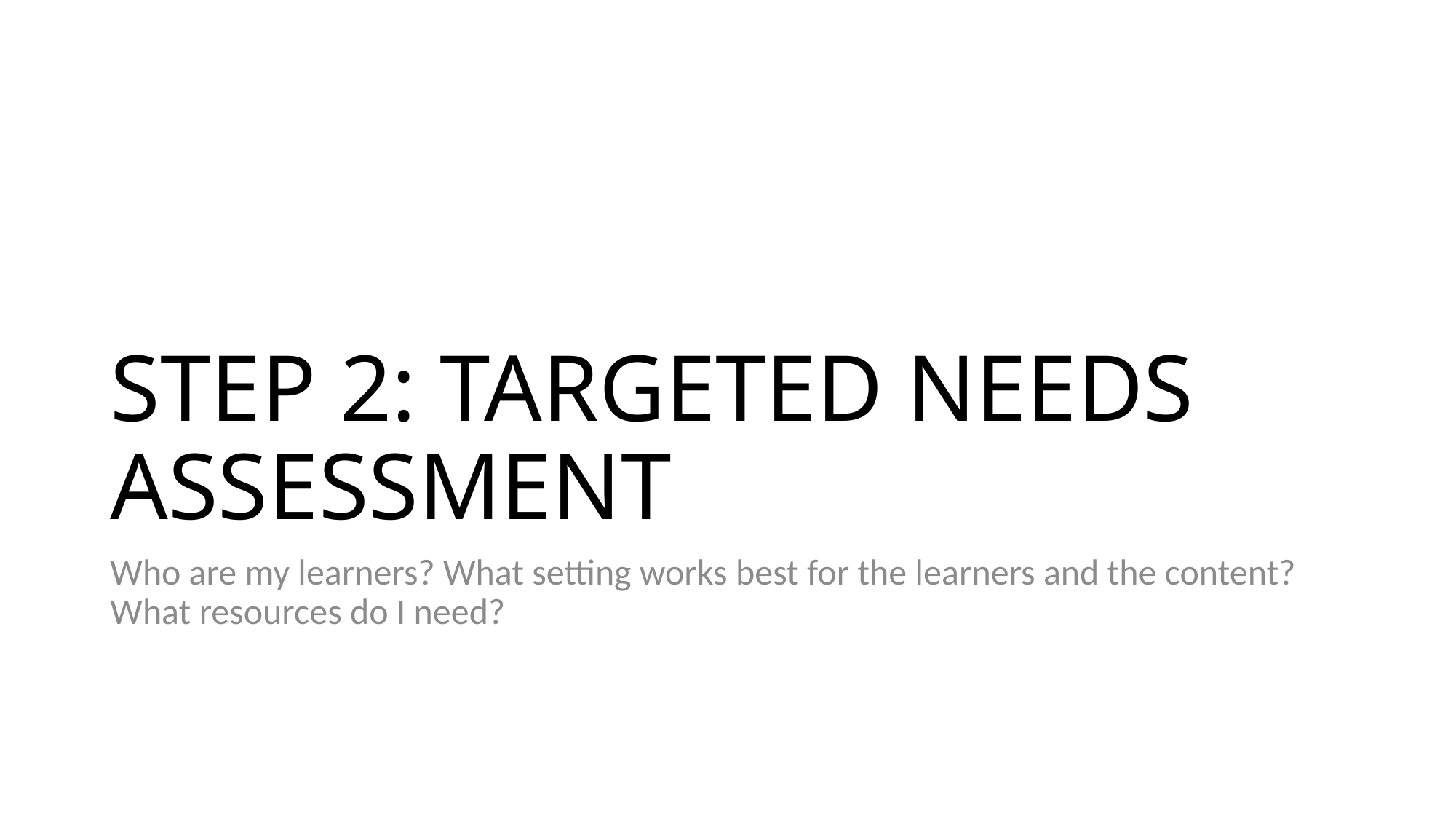

# STEP 2: TARGETED NEEDS ASSESSMENT
Who are my learners? What setting works best for the learners and the content? What resources do I need?

## Slide 20
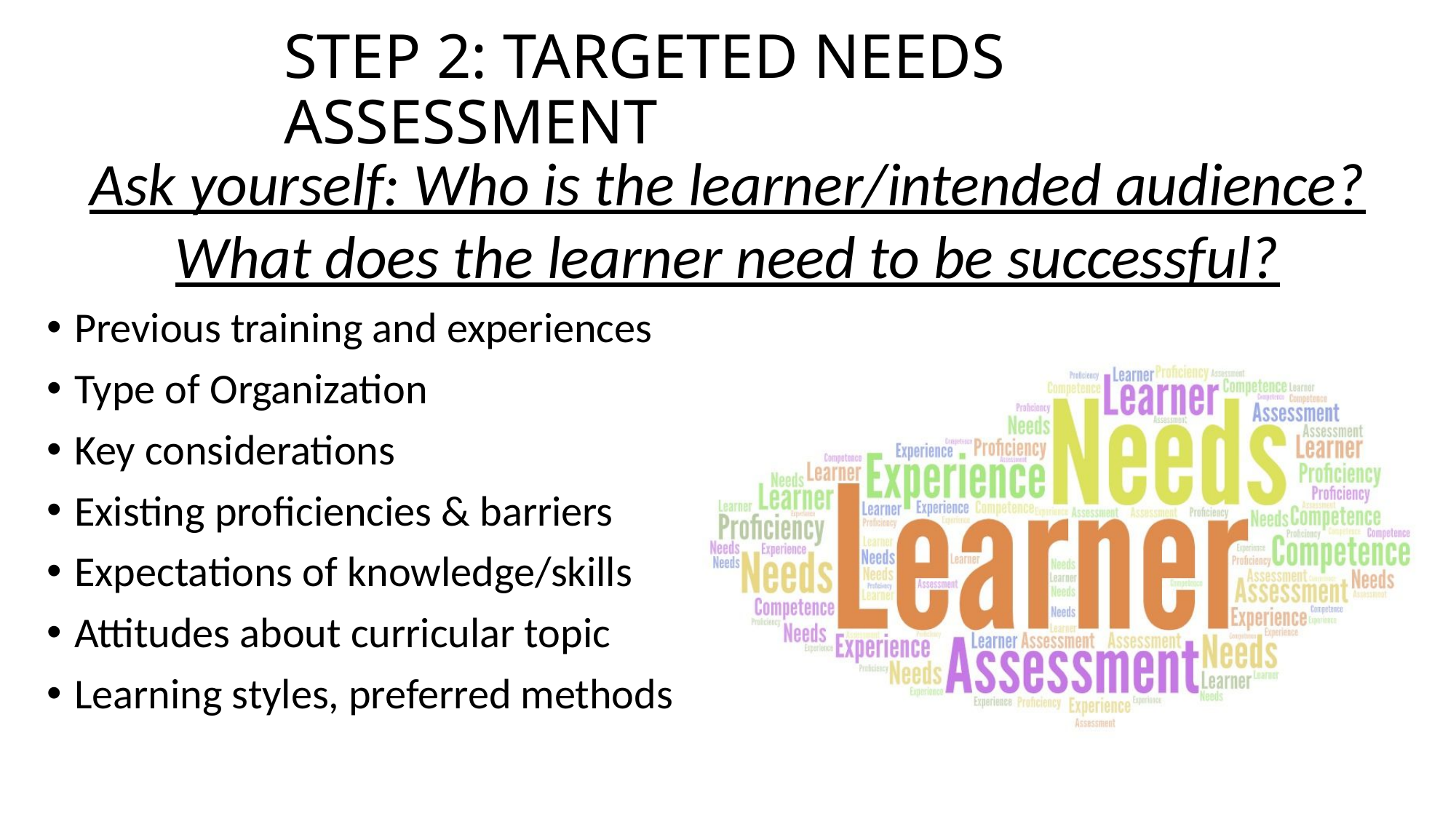

# STEP 2: TARGETED NEEDS ASSESSMENT
Ask yourself: Who is the learner/intended audience?
What does the learner need to be successful?
Previous training and experiences
Type of Organization
Key considerations
Existing proficiencies & barriers
Expectations of knowledge/skills
Attitudes about curricular topic
Learning styles, preferred methods

## Slide 21
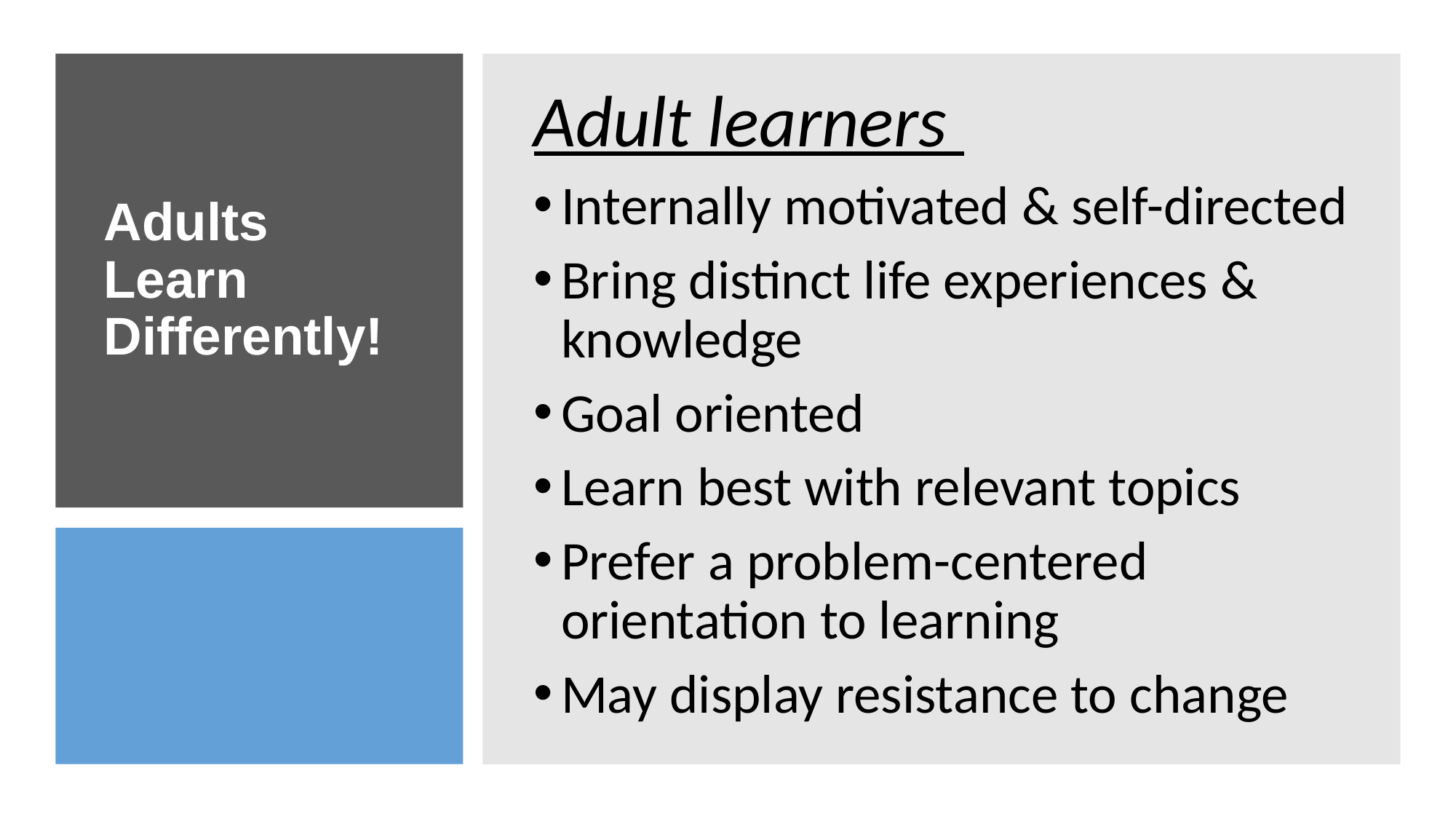

Adult learners
Internally motivated & self-directed
Bring distinct life experiences & knowledge
Goal oriented
Learn best with relevant topics
Prefer a problem-centered orientation to learning
May display resistance to change
# Adults Learn Differently!

## Slide 22
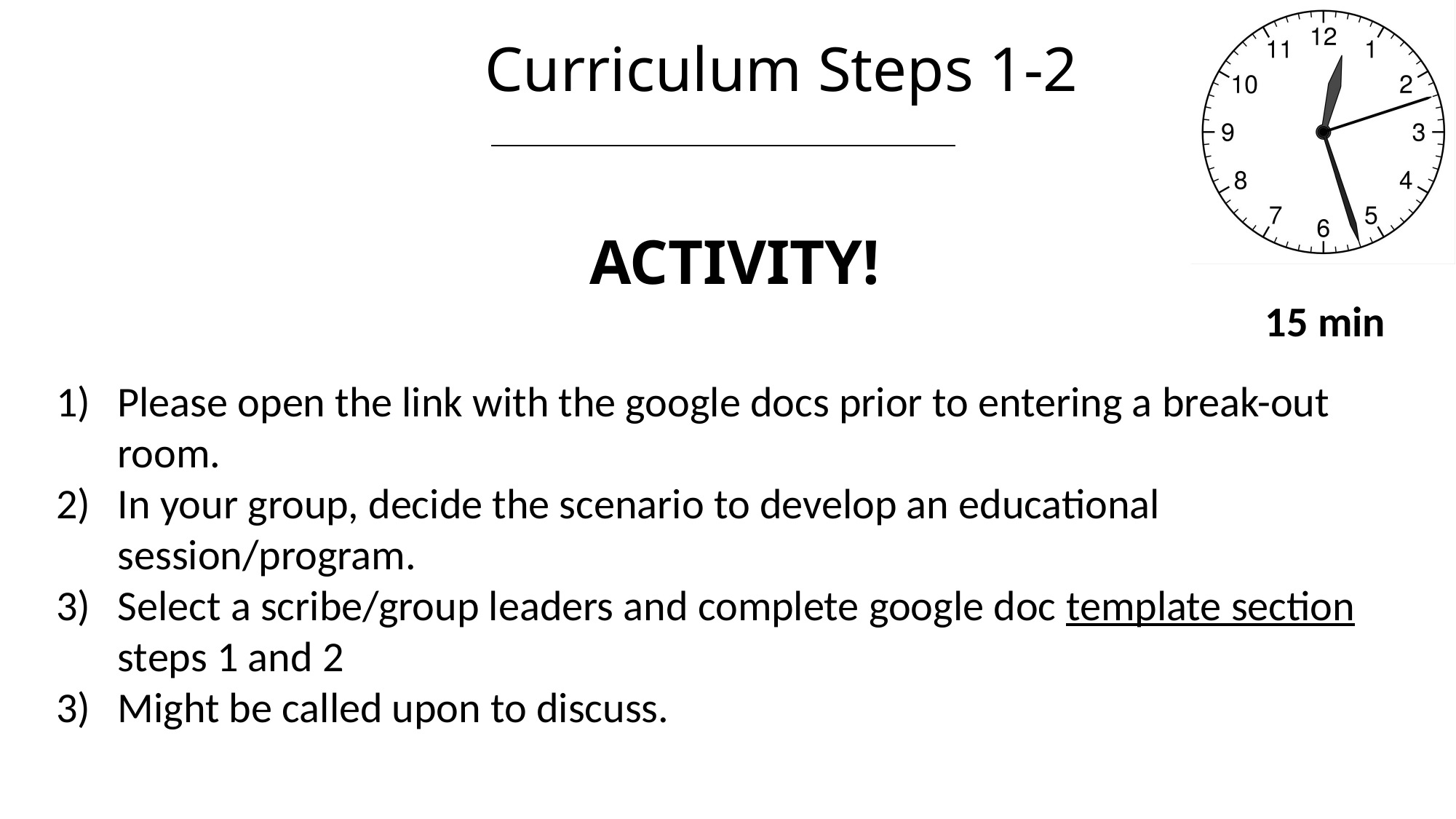

Curriculum Steps 1-2
ACTIVITY!
15 min
Please open the link with the google docs prior to entering a break-out room.
In your group, decide the scenario to develop an educational session/program.
Select a scribe/group leaders and complete google doc template section steps 1 and 2
Might be called upon to discuss.

## Slide 23
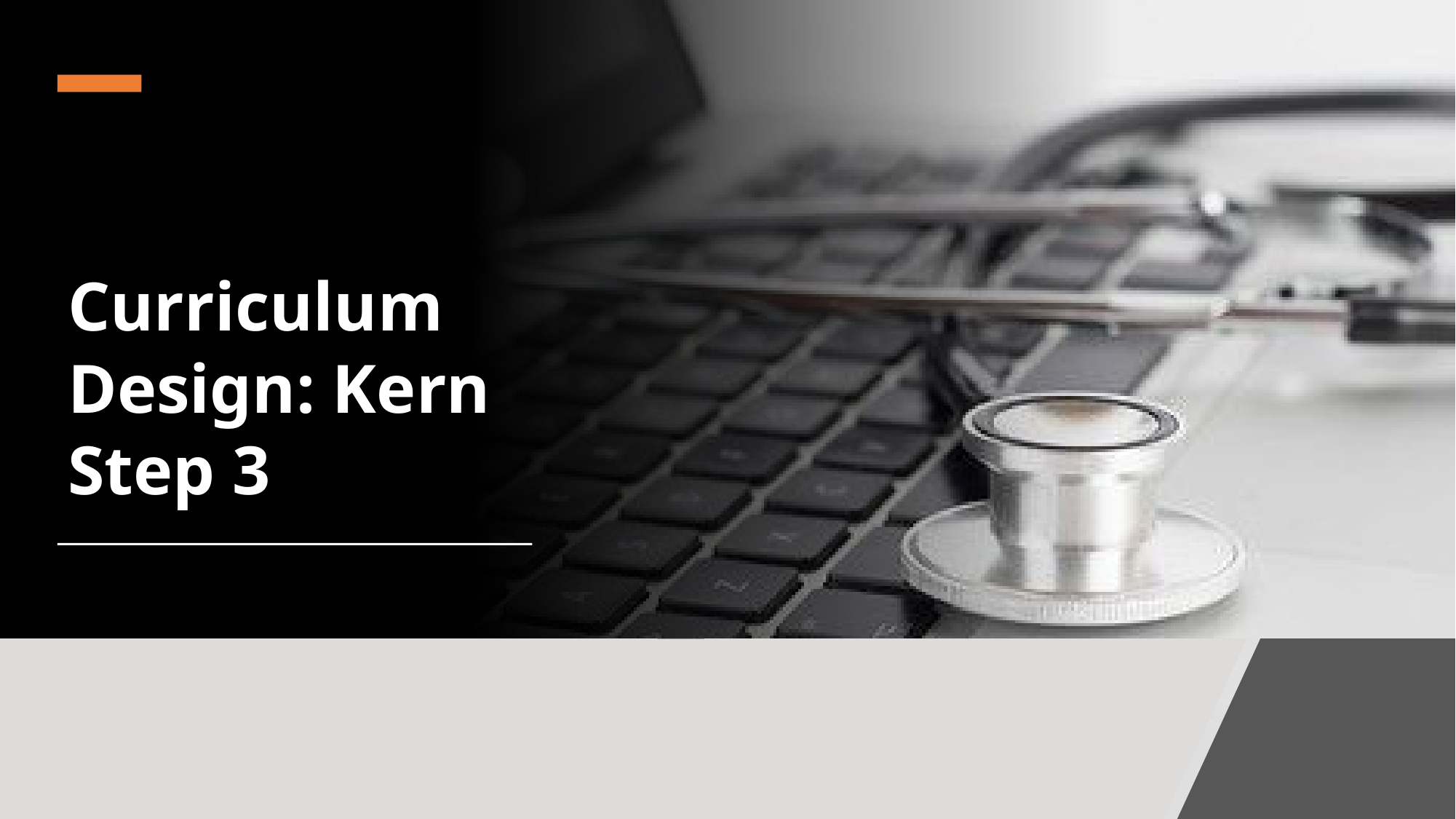

# Curriculum Design: Kern Step 3

## Slide 24
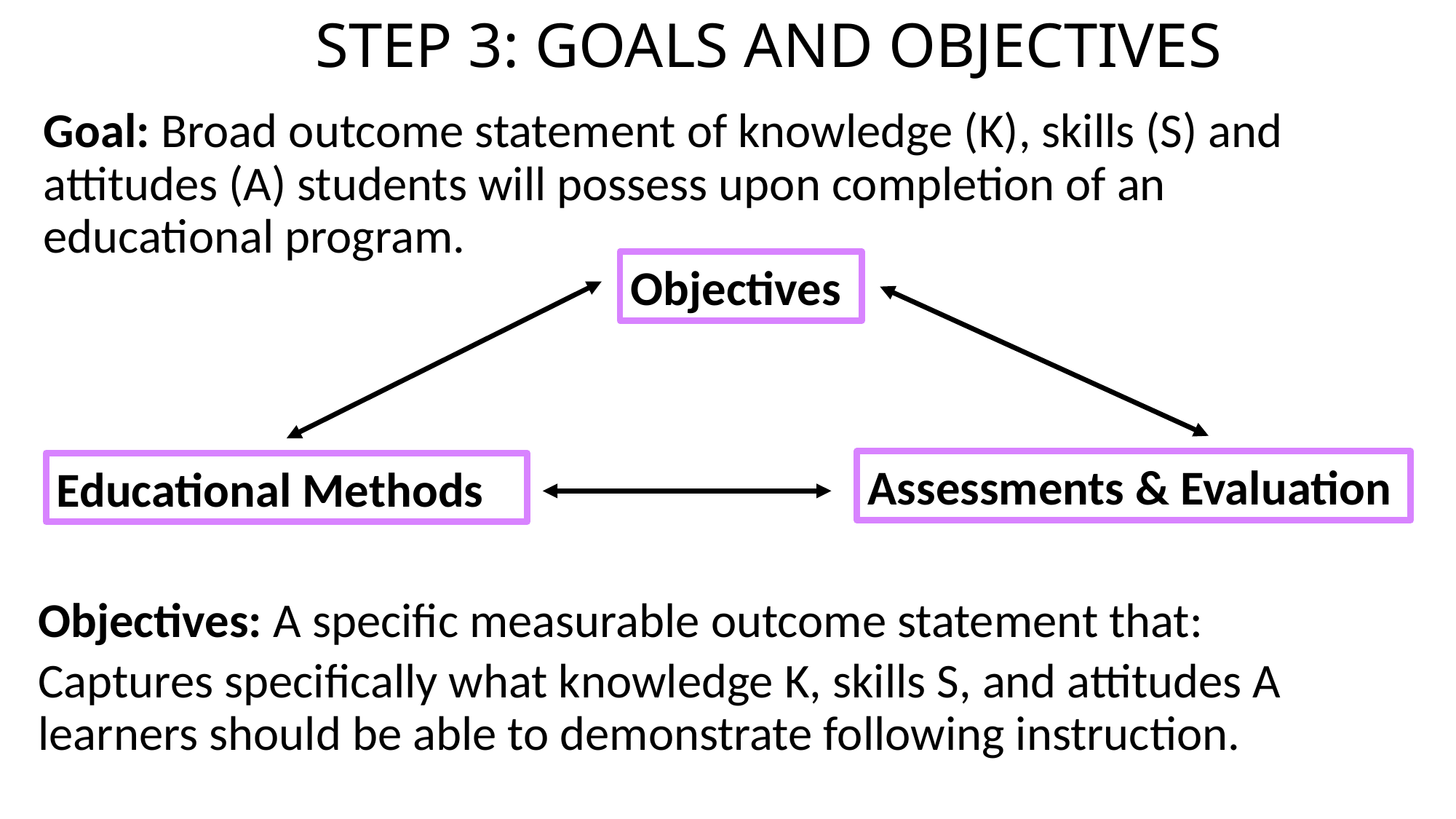

# STEP 3: GOALS AND OBJECTIVES
Goal: Broad outcome statement of knowledge (K), skills (S) and attitudes (A) students will possess upon completion of an
educational program.
Objectives
Assessments & Evaluation
Educational Methods
Objectives: A specific measurable outcome statement that:
Captures specifically what knowledge K, skills S, and attitudes A learners should be able to demonstrate following instruction.

## Slide 25
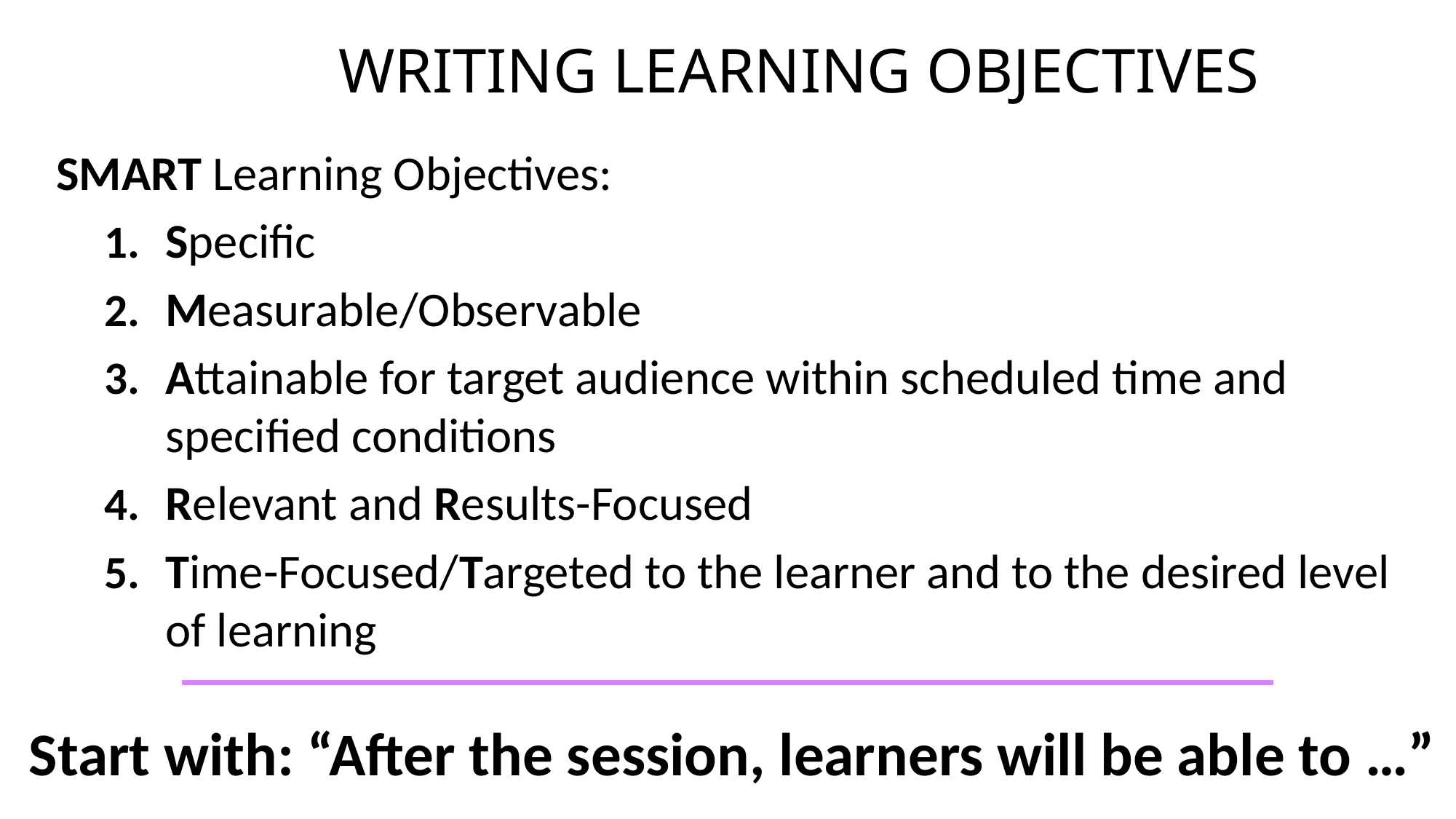

WRITING LEARNING OBJECTIVES
# Definition
SMART Learning Objectives:
Specific
Measurable/Observable
Attainable for target audience within scheduled time and specified conditions
Relevant and Results-Focused
Time-Focused/Targeted to the learner and to the desired level of learning
Start with: “After the session, learners will be able to …”

## Slide 26
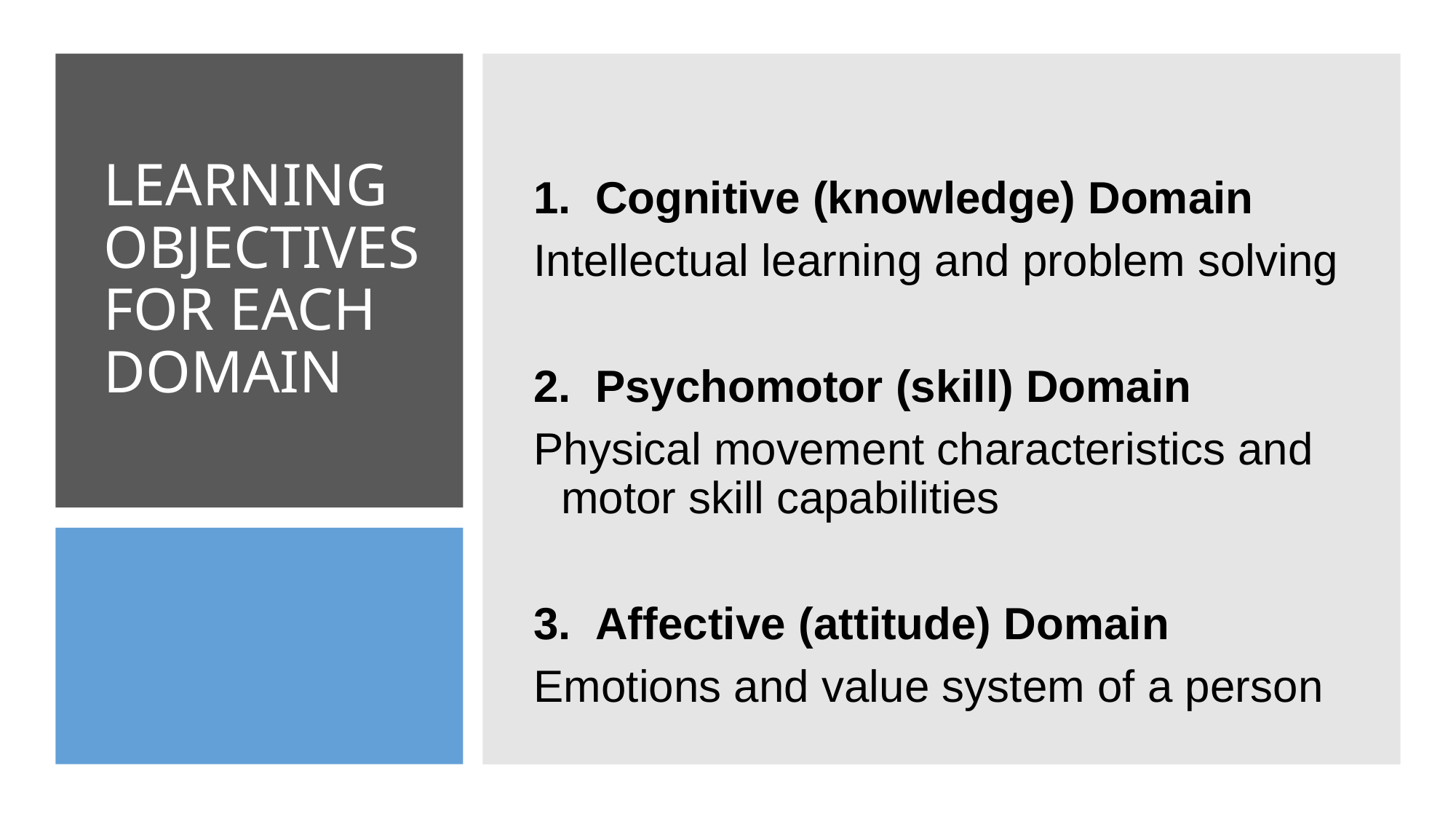

Cognitive (knowledge) Domain
Intellectual learning and problem solving
Psychomotor (skill) Domain
Physical movement characteristics and motor skill capabilities
Affective (attitude) Domain
Emotions and value system of a person
# LEARNING OBJECTIVES FOR EACH DOMAIN

## Slide 27
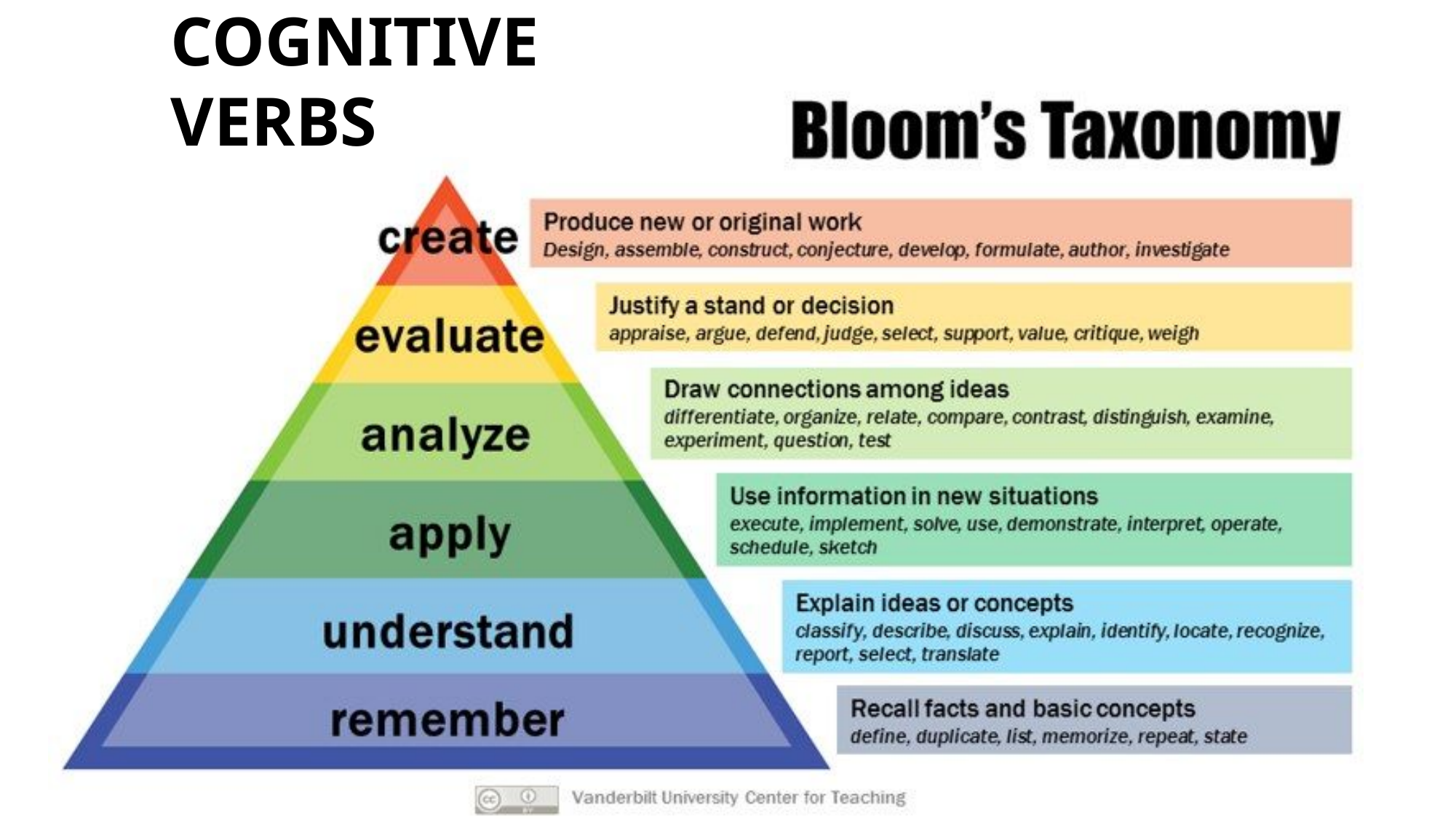

COGNITIVE VERBS
# Definition

## Slide 28
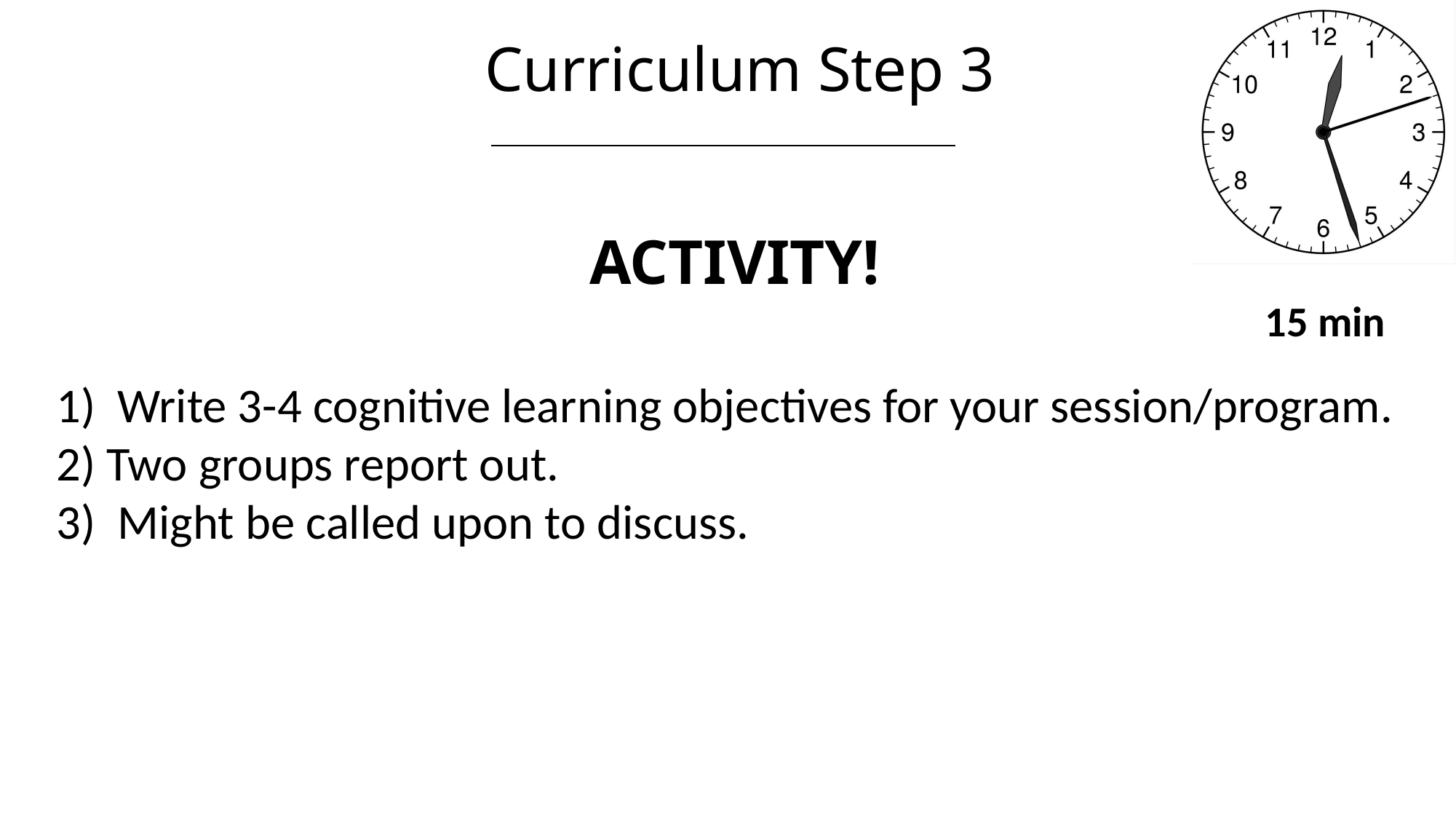

Curriculum Step 3
ACTIVITY!
15 min
1) Write 3-4 cognitive learning objectives for your session/program.
2) Two groups report out.
Might be called upon to discuss.

## Slide 29
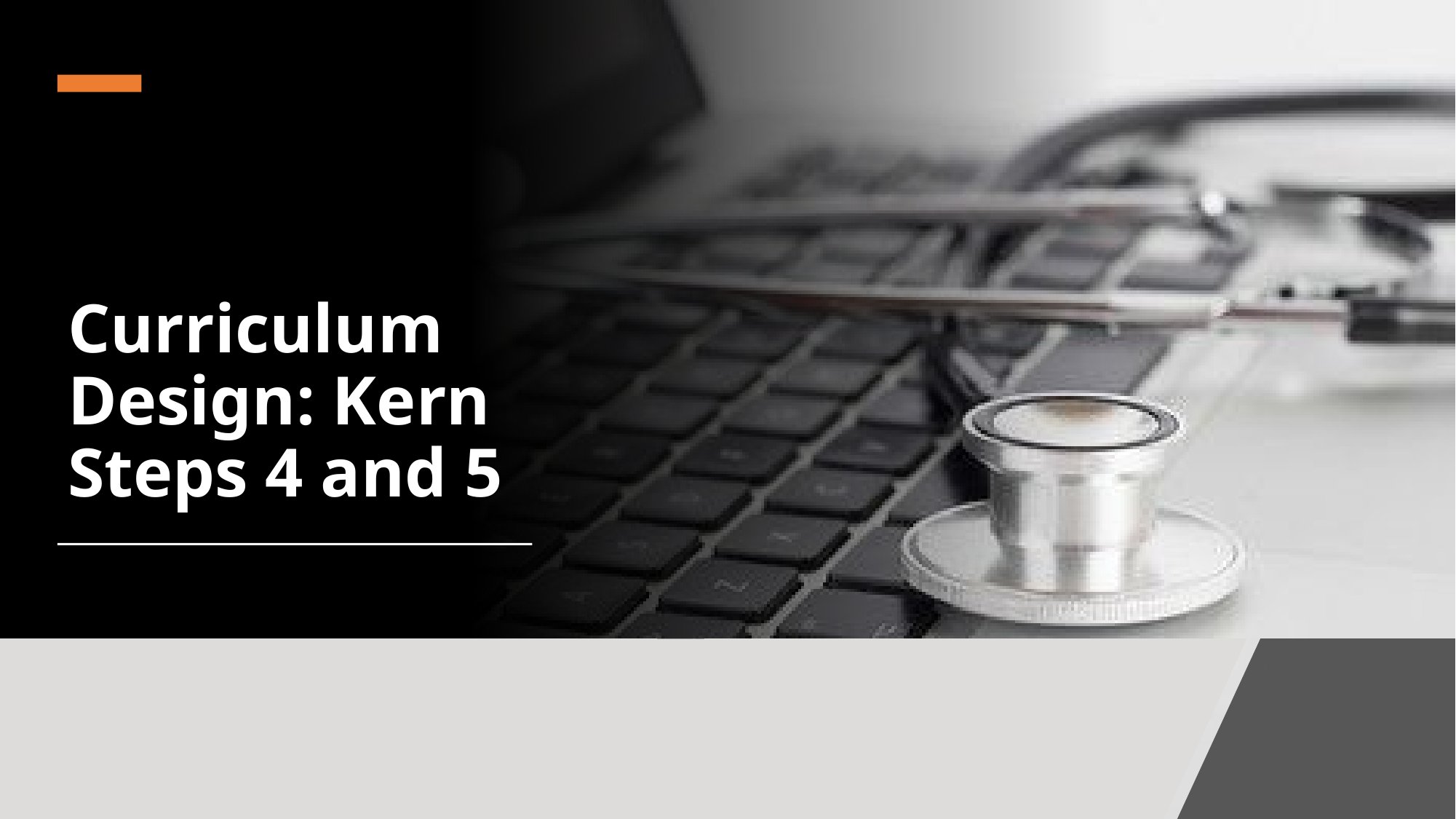

# Curriculum Design: Kern Steps 4 and 5

## Slide 30
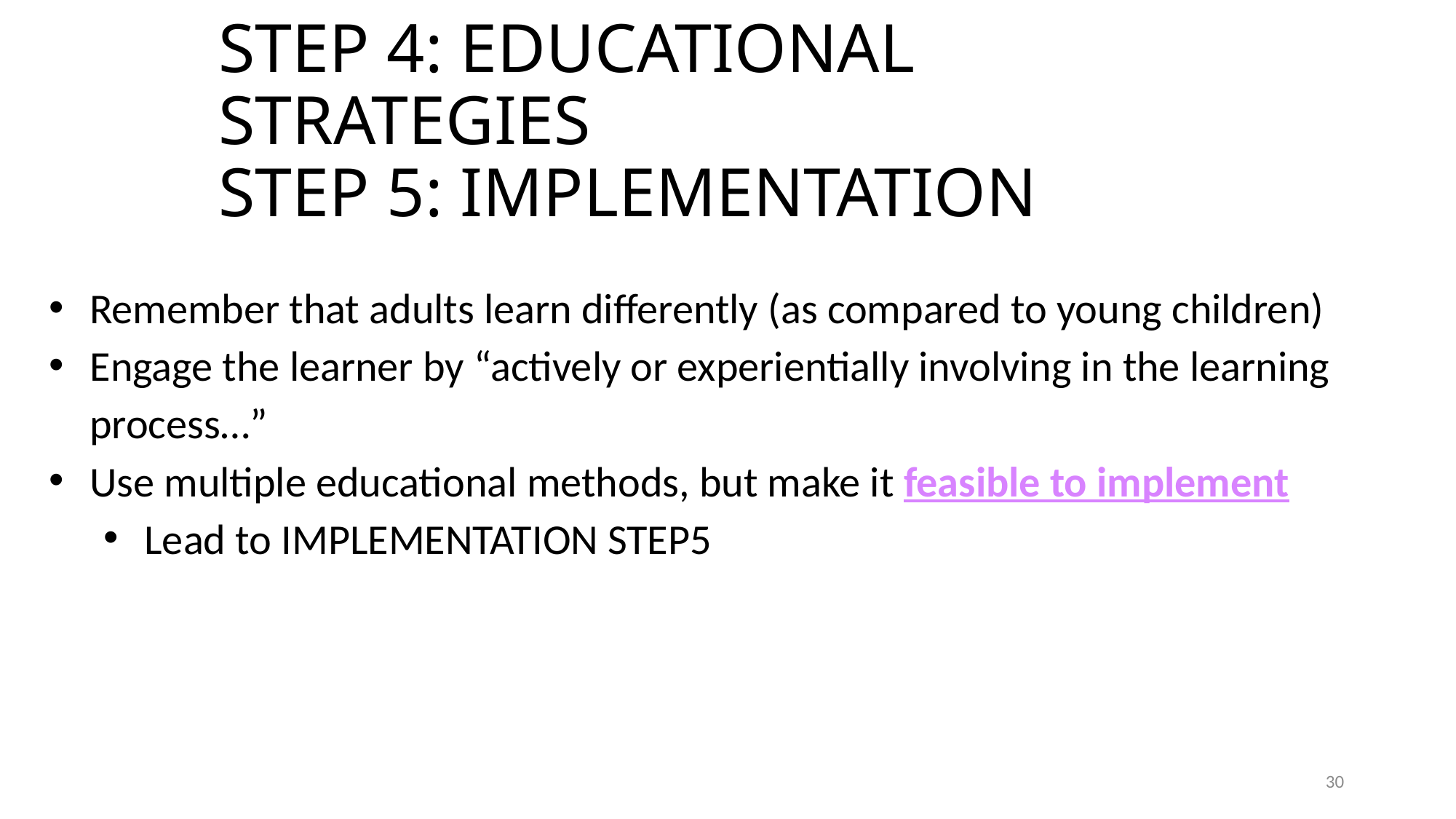

STEP 4: EDUCATIONAL STRATEGIES
STEP 5: IMPLEMENTATION
Remember that adults learn differently (as compared to young children)
Engage the learner by “actively or experientially involving in the learning process…”
Use multiple educational methods, but make it feasible to implement
Lead to IMPLEMENTATION STEP5
30

## Slide 31
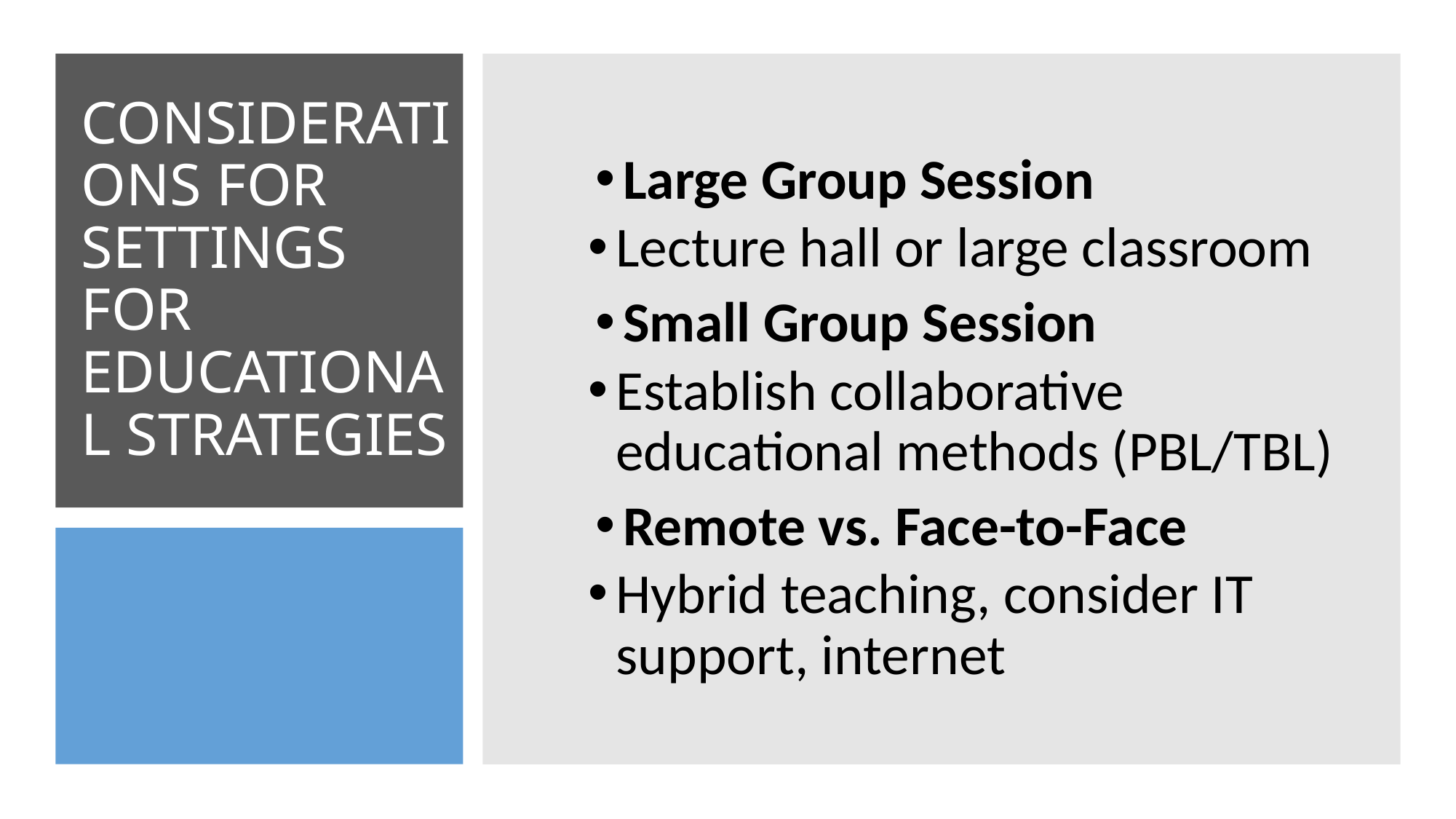

Large Group Session
Lecture hall or large classroom
Small Group Session
Establish collaborative educational methods (PBL/TBL)
Remote vs. Face-to-Face
Hybrid teaching, consider IT support, internet
Considerations for Settings for
Educational Strategies

## Slide 32
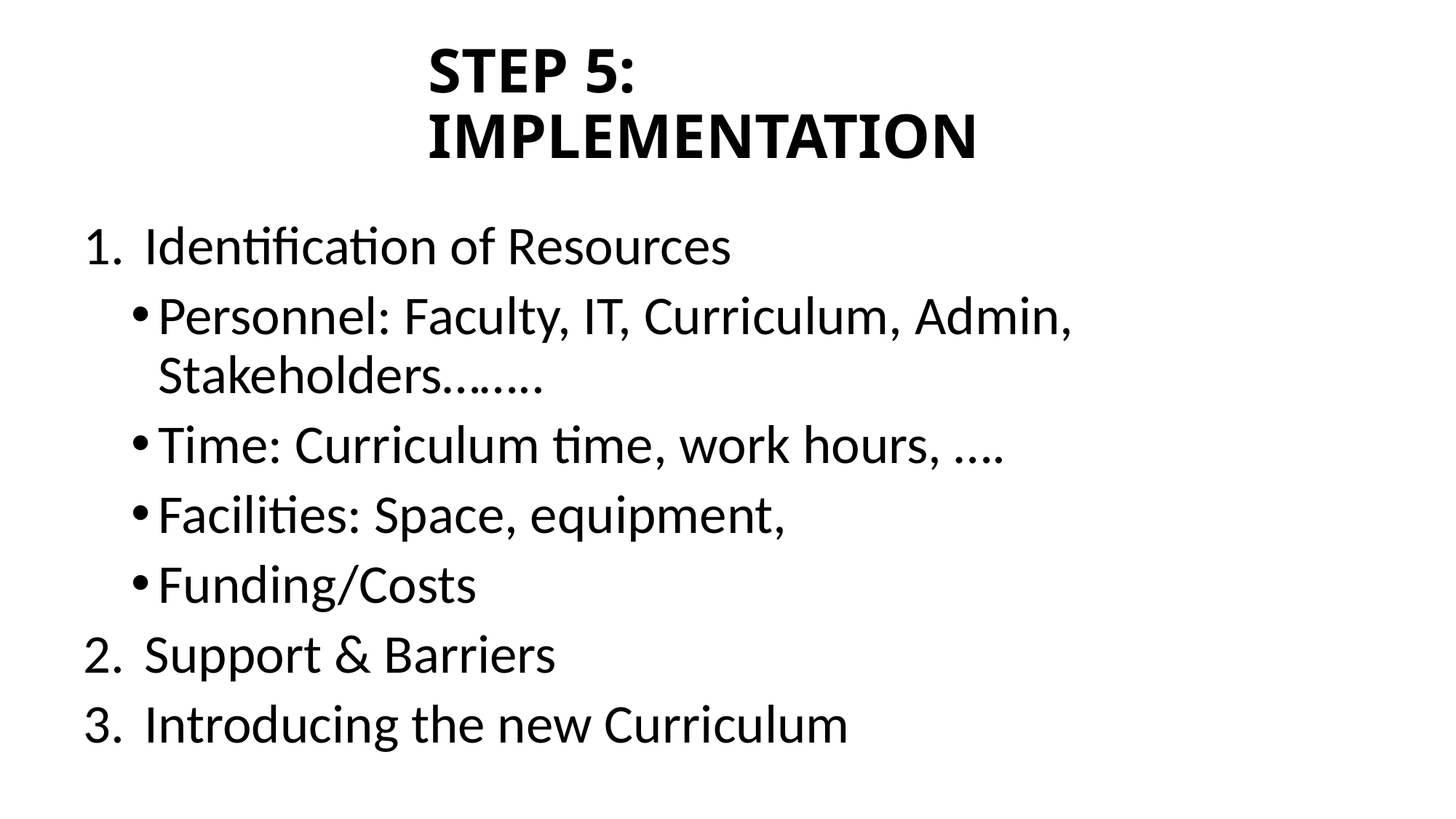

STEP 5: IMPLEMENTATION
Identification of Resources
Personnel: Faculty, IT, Curriculum, Admin, Stakeholders……..
Time: Curriculum time, work hours, ….
Facilities: Space, equipment,
Funding/Costs
Support & Barriers
Introducing the new Curriculum

## Slide 33
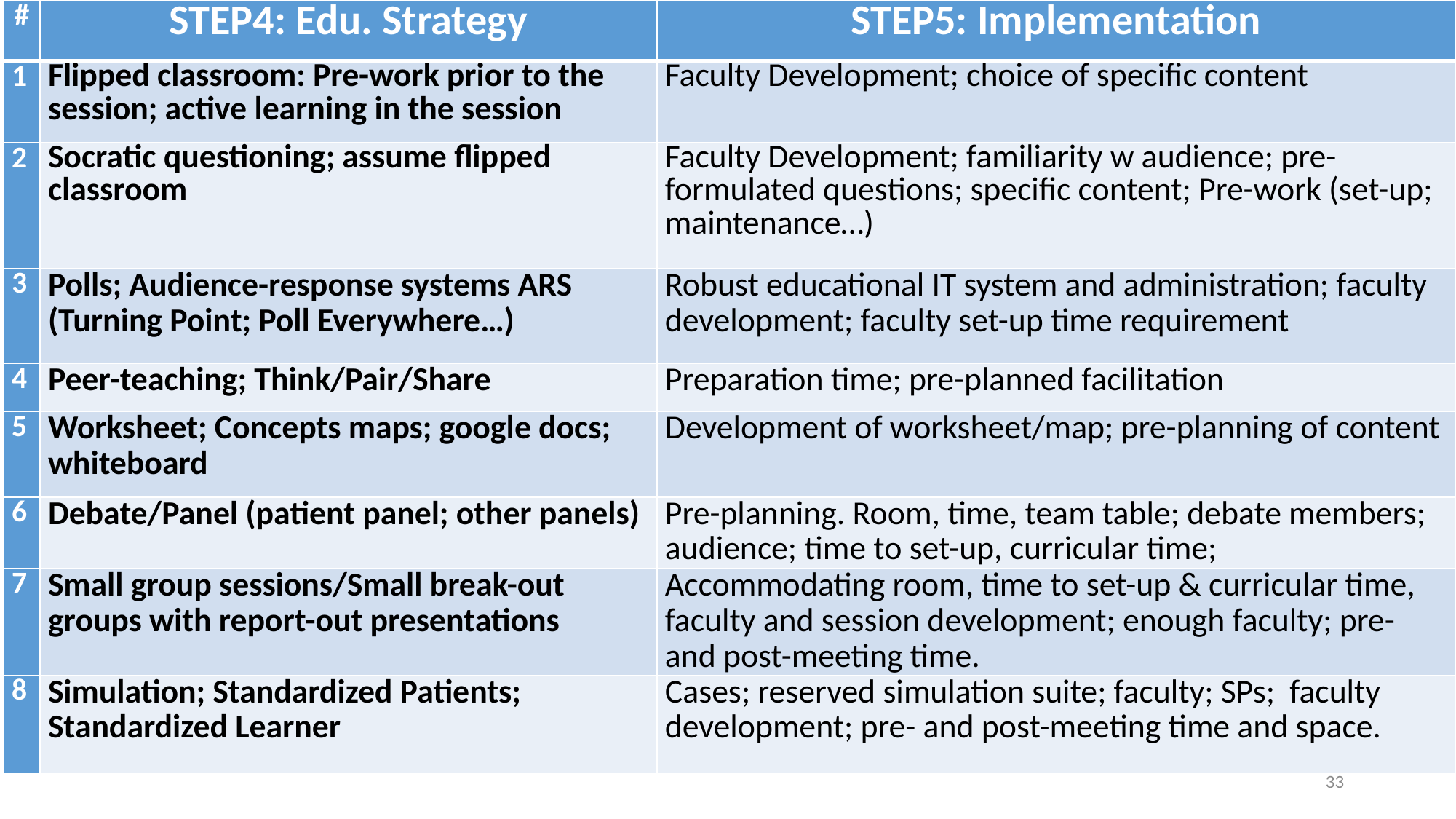

| # | STEP4: Edu. Strategy | STEP5: Implementation |
| --- | --- | --- |
| 1 | Flipped classroom: Pre-work prior to the session; active learning in the session | Faculty Development; choice of specific content |
| 2 | Socratic questioning; assume flipped classroom | Faculty Development; familiarity w audience; pre-formulated questions; specific content; Pre-work (set-up; maintenance…) |
| 3 | Polls; Audience-response systems ARS (Turning Point; Poll Everywhere…) | Robust educational IT system and administration; faculty development; faculty set-up time requirement |
| 4 | Peer-teaching; Think/Pair/Share | Preparation time; pre-planned facilitation |
| 5 | Worksheet; Concepts maps; google docs; whiteboard | Development of worksheet/map; pre-planning of content |
| 6 | Debate/Panel (patient panel; other panels) | Pre-planning. Room, time, team table; debate members; audience; time to set-up, curricular time; |
| 7 | Small group sessions/Small break-out groups with report-out presentations | Accommodating room, time to set-up & curricular time, faculty and session development; enough faculty; pre- and post-meeting time. |
| 8 | Simulation; Standardized Patients; Standardized Learner | Cases; reserved simulation suite; faculty; SPs; faculty development; pre- and post-meeting time and space. |
33

## Slide 34
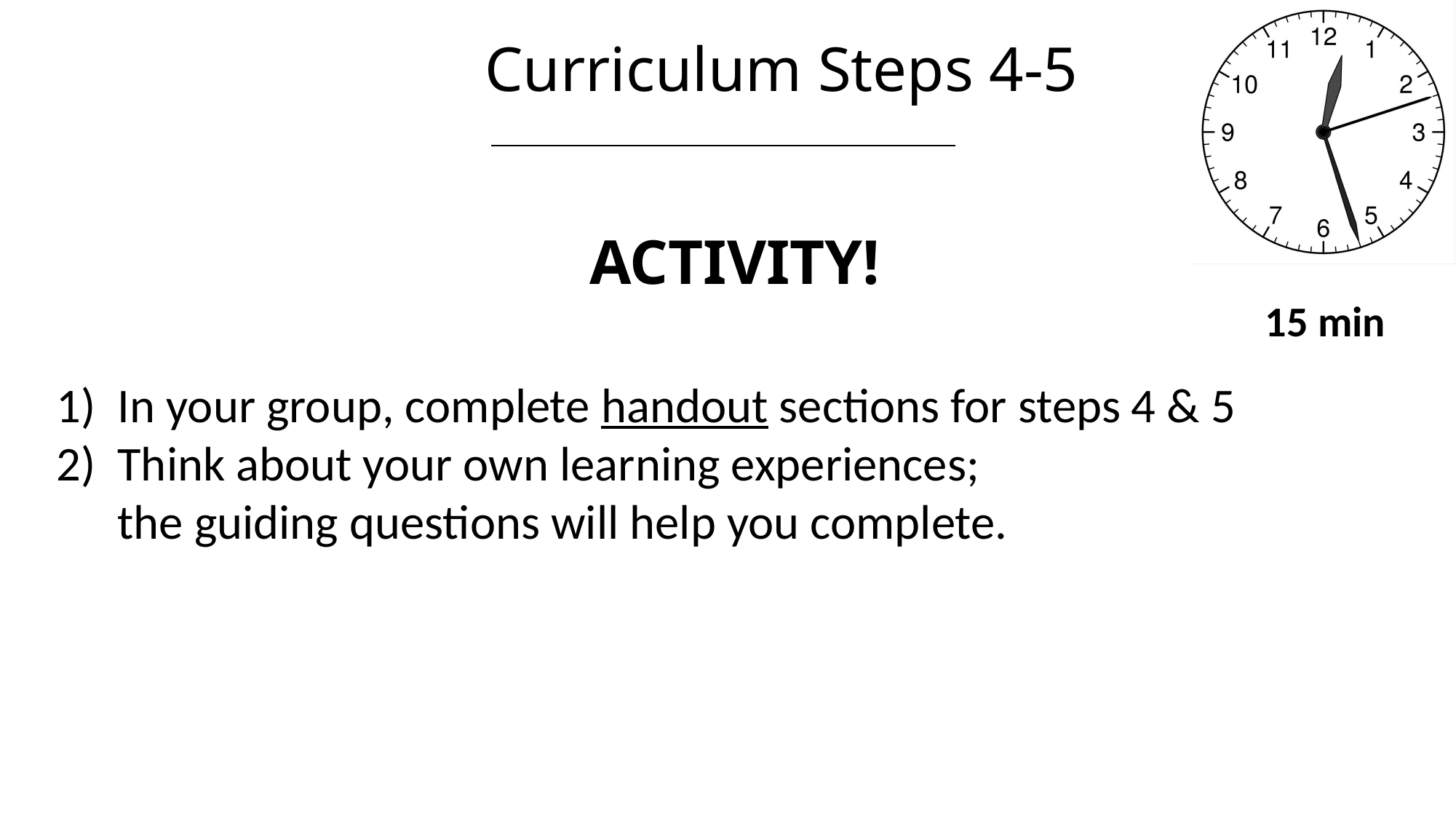

Curriculum Steps 4-5
ACTIVITY!
15 min
In your group, complete handout sections for steps 4 & 5
Think about your own learning experiences;
the guiding questions will help you complete.

## Slide 35
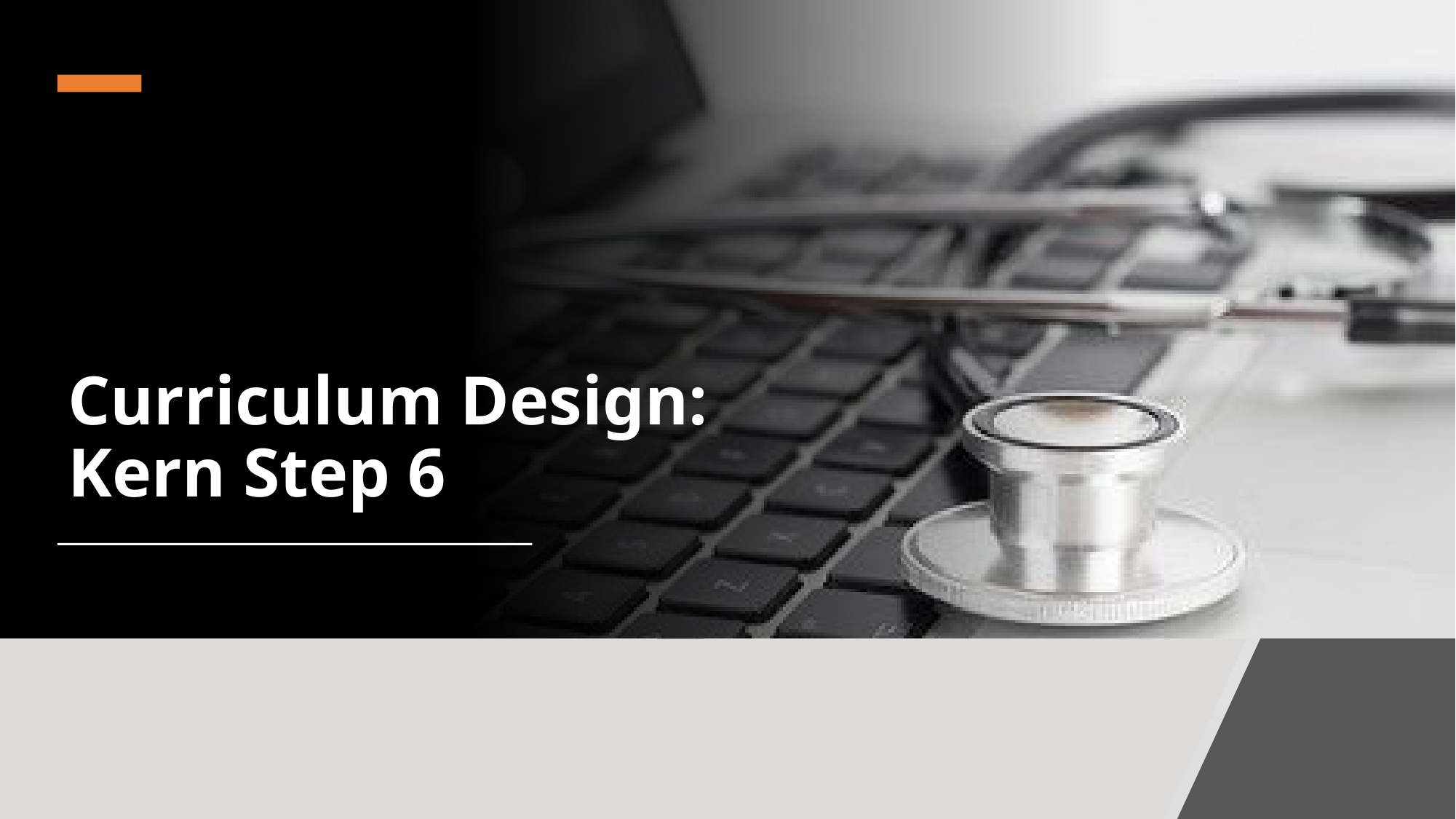

# Curriculum Design: Kern Step 6

## Slide 36
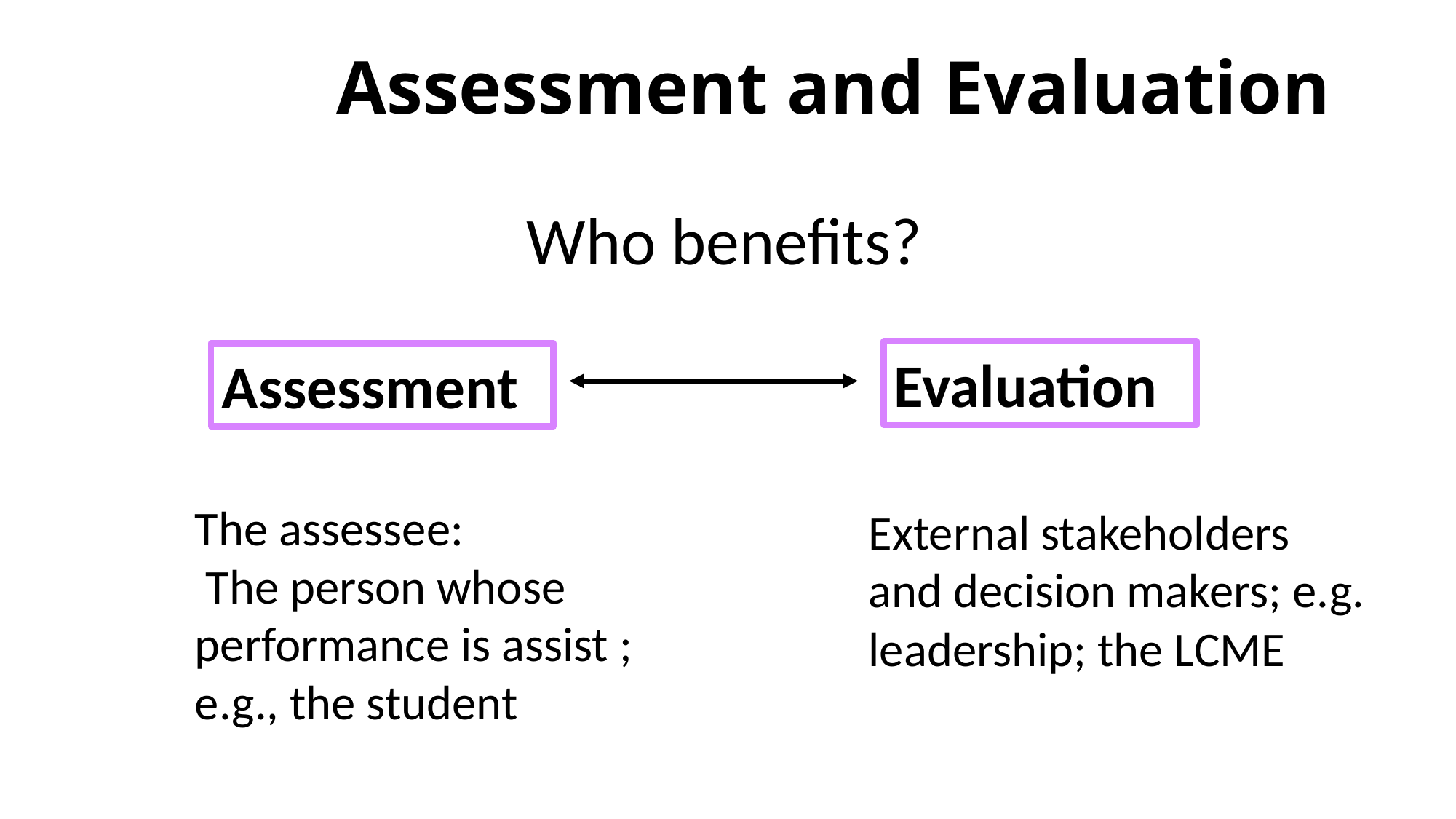

# Assessment and Evaluation
Who benefits?
Evaluation
Assessment
The assessee:
 The person whose performance is assist ; e.g., the student
External stakeholders and decision makers; e.g. leadership; the LCME
36

## Slide 37
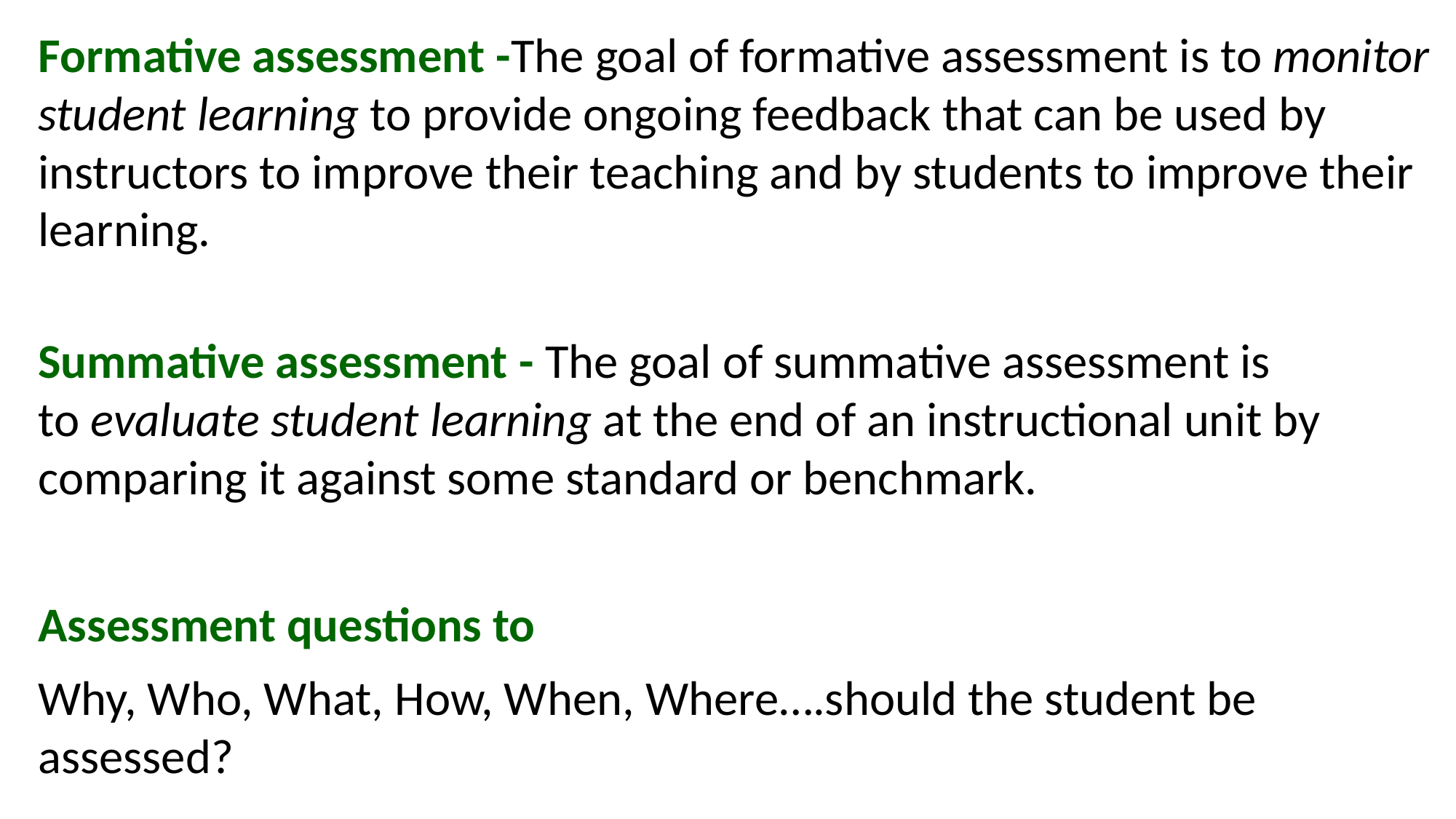

Formative assessment -The goal of formative assessment is to monitor student learning to provide ongoing feedback that can be used by instructors to improve their teaching and by students to improve their learning.
Summative assessment - The goal of summative assessment is to evaluate student learning at the end of an instructional unit by comparing it against some standard or benchmark.
Assessment questions to
Why, Who, What, How, When, Where….should the student be assessed?

## Slide 38
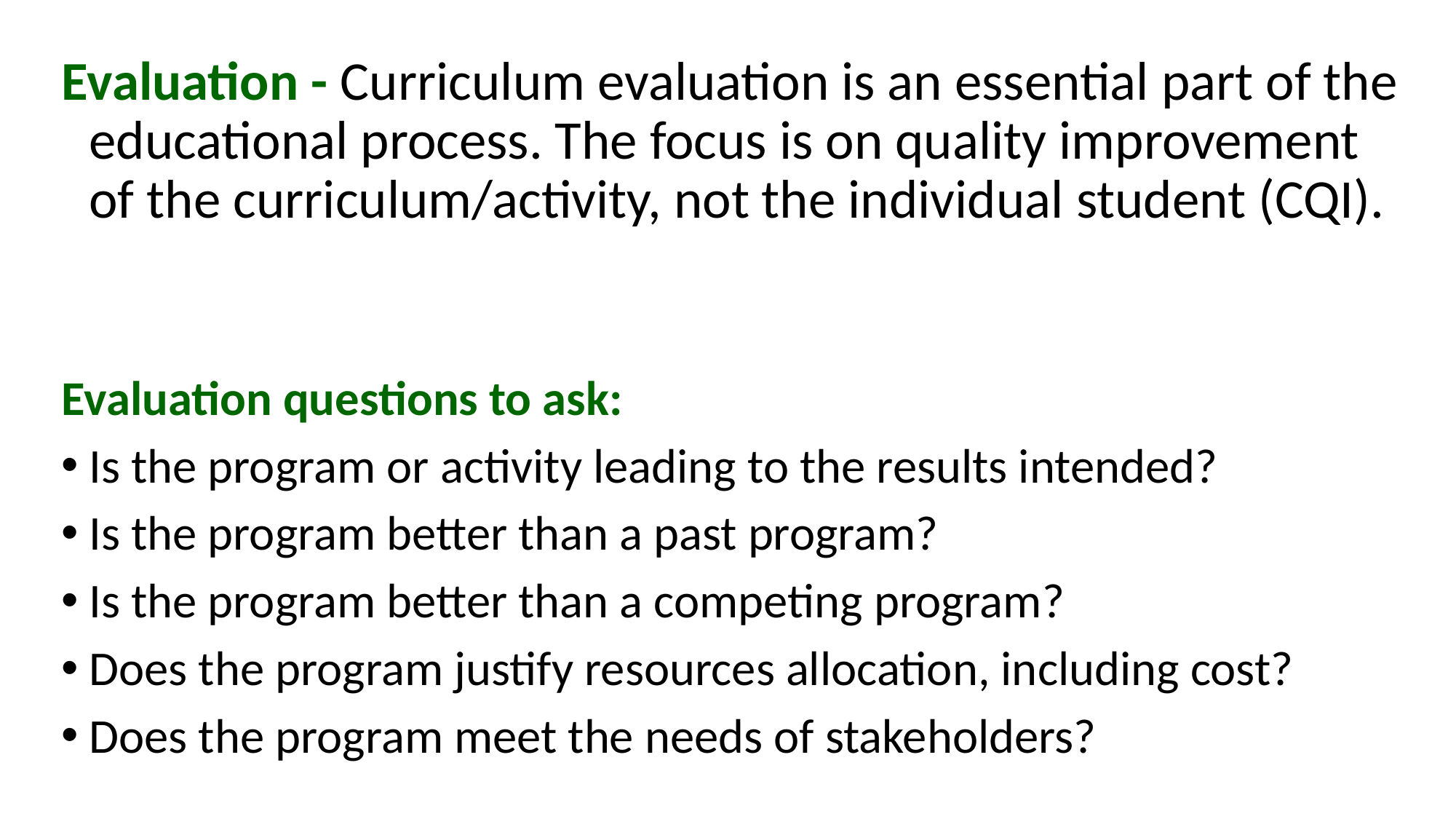

Evaluation - Curriculum evaluation is an essential part of the educational process. The focus is on quality improvement of the curriculum/activity, not the individual student (CQI).
Evaluation questions to ask:
Is the program or activity leading to the results intended?
Is the program better than a past program?
Is the program better than a competing program?
Does the program justify resources allocation, including cost?
Does the program meet the needs of stakeholders?

## Slide 39
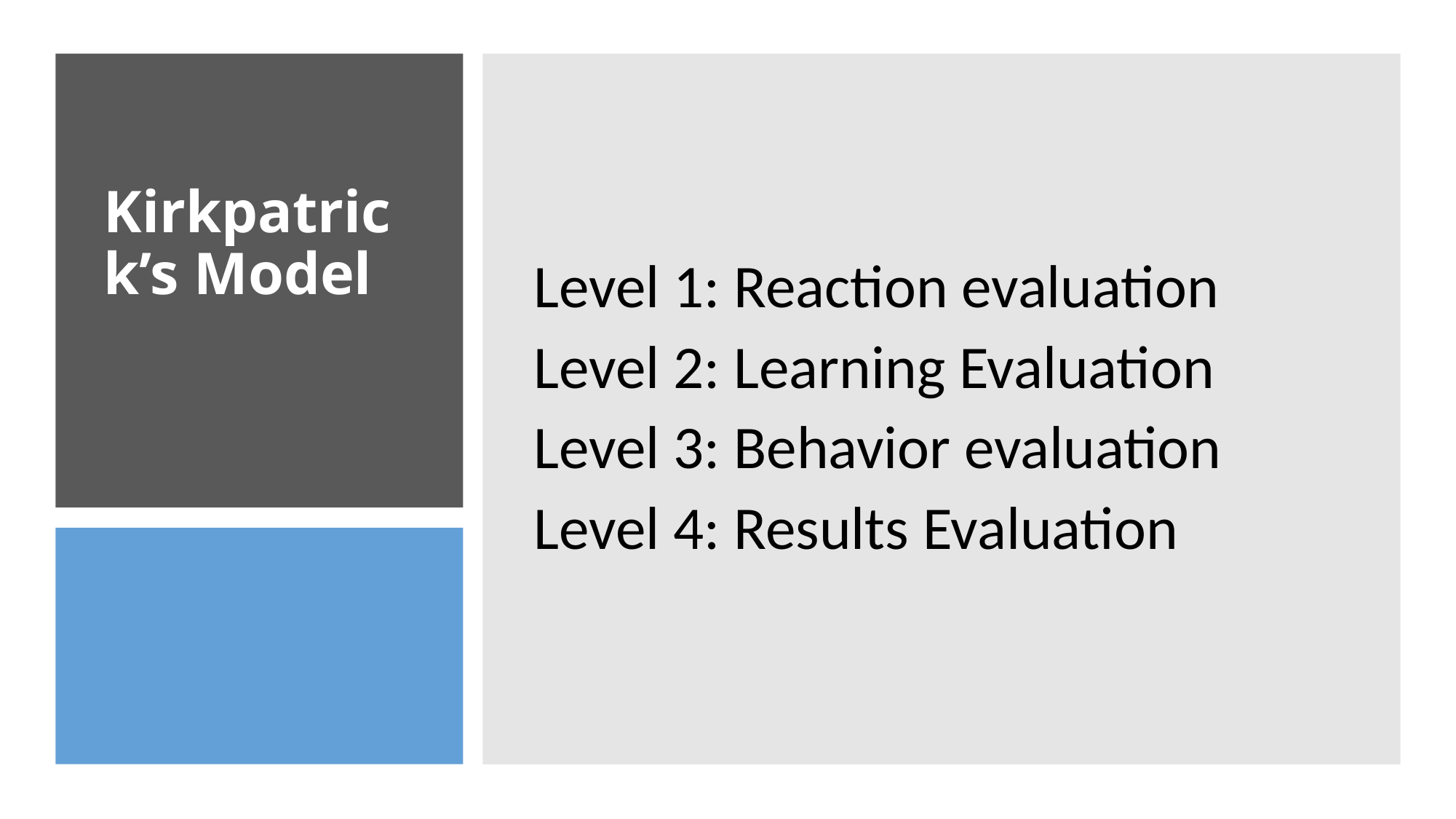

Level 1: Reaction evaluation
Level 2: Learning Evaluation
Level 3: Behavior evaluation
Level 4: Results Evaluation
Kirkpatrick’s Model

## Slide 40
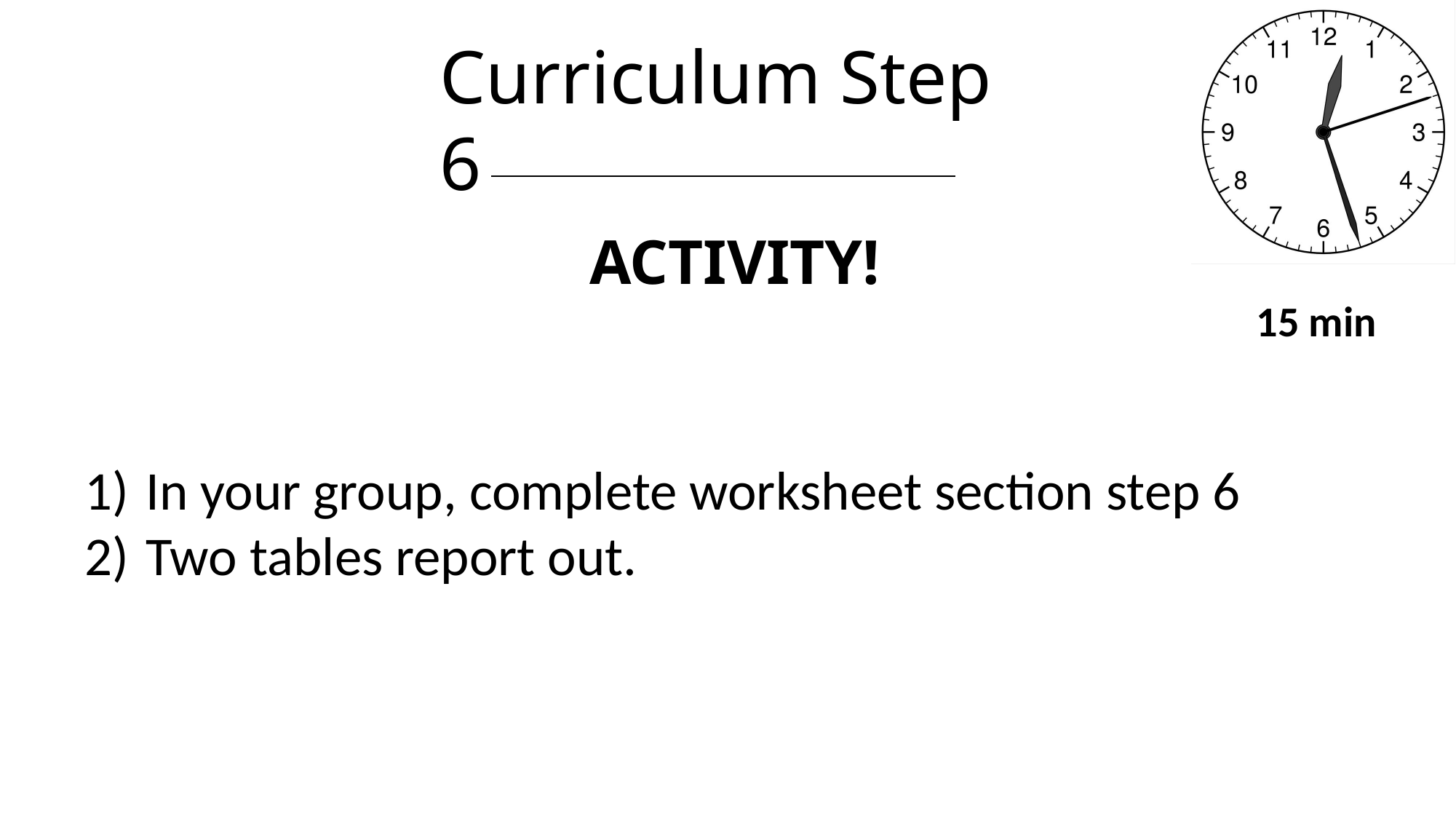

Curriculum Step 6
ACTIVITY!
15 min
In your group, complete worksheet section step 6
Two tables report out.

## Slide 41
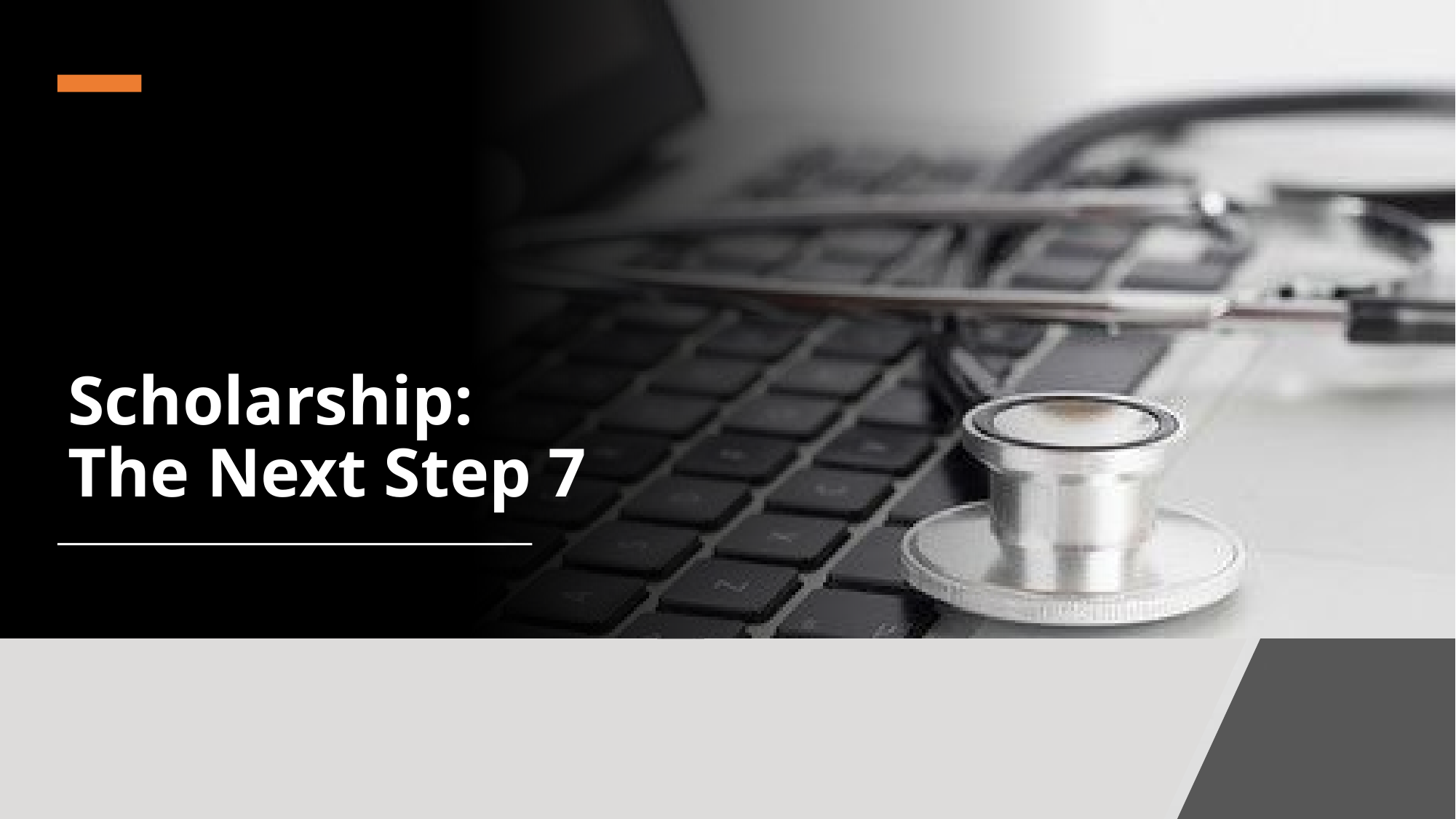

# Scholarship: The Next Step 7

## Slide 42
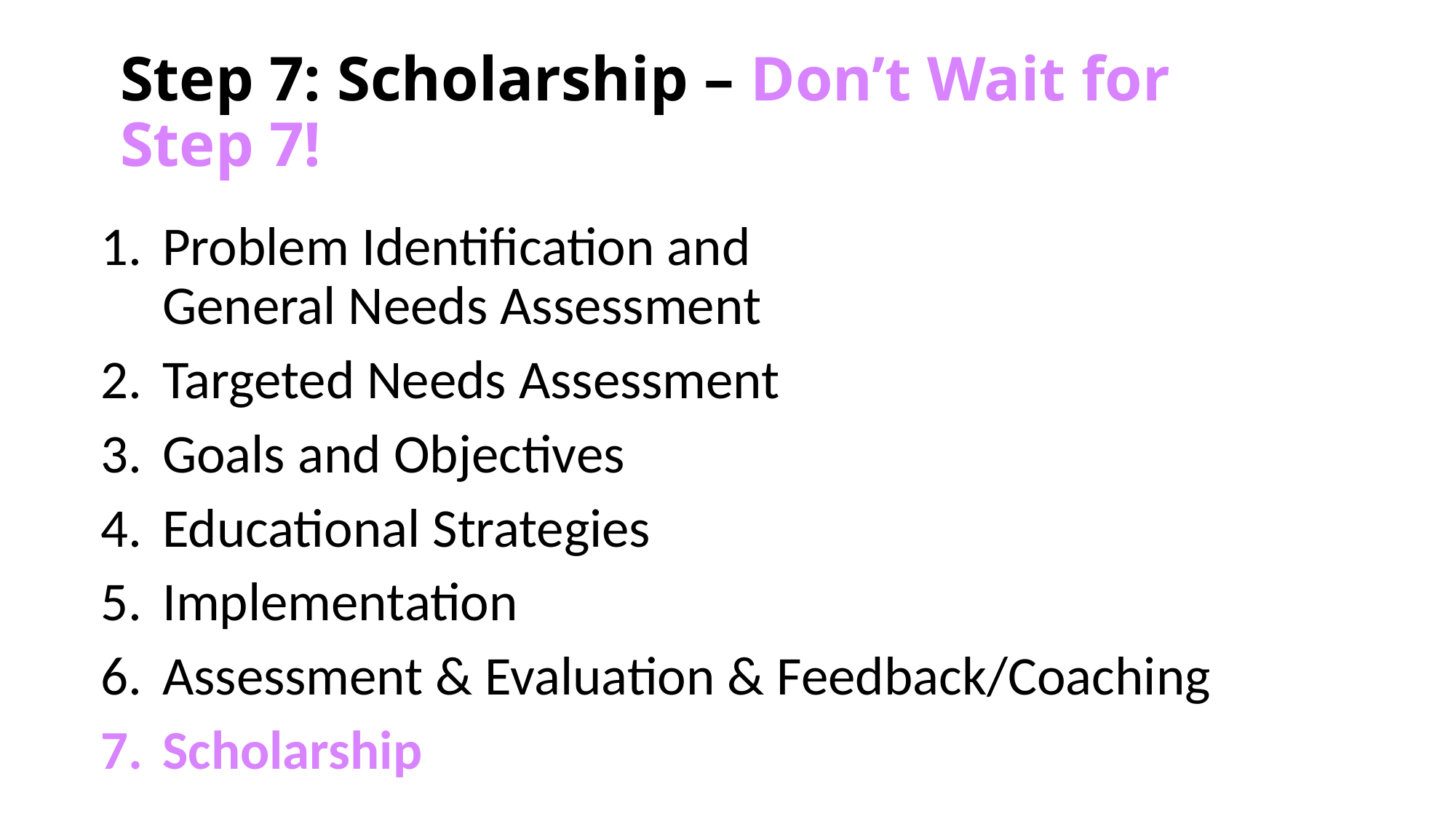

# Step 7: Scholarship – Don’t Wait for Step 7!
Problem Identification and
General Needs Assessment
Targeted Needs Assessment
Goals and Objectives
Educational Strategies
Implementation
Assessment & Evaluation & Feedback/Coaching
Scholarship
42

## Slide 43
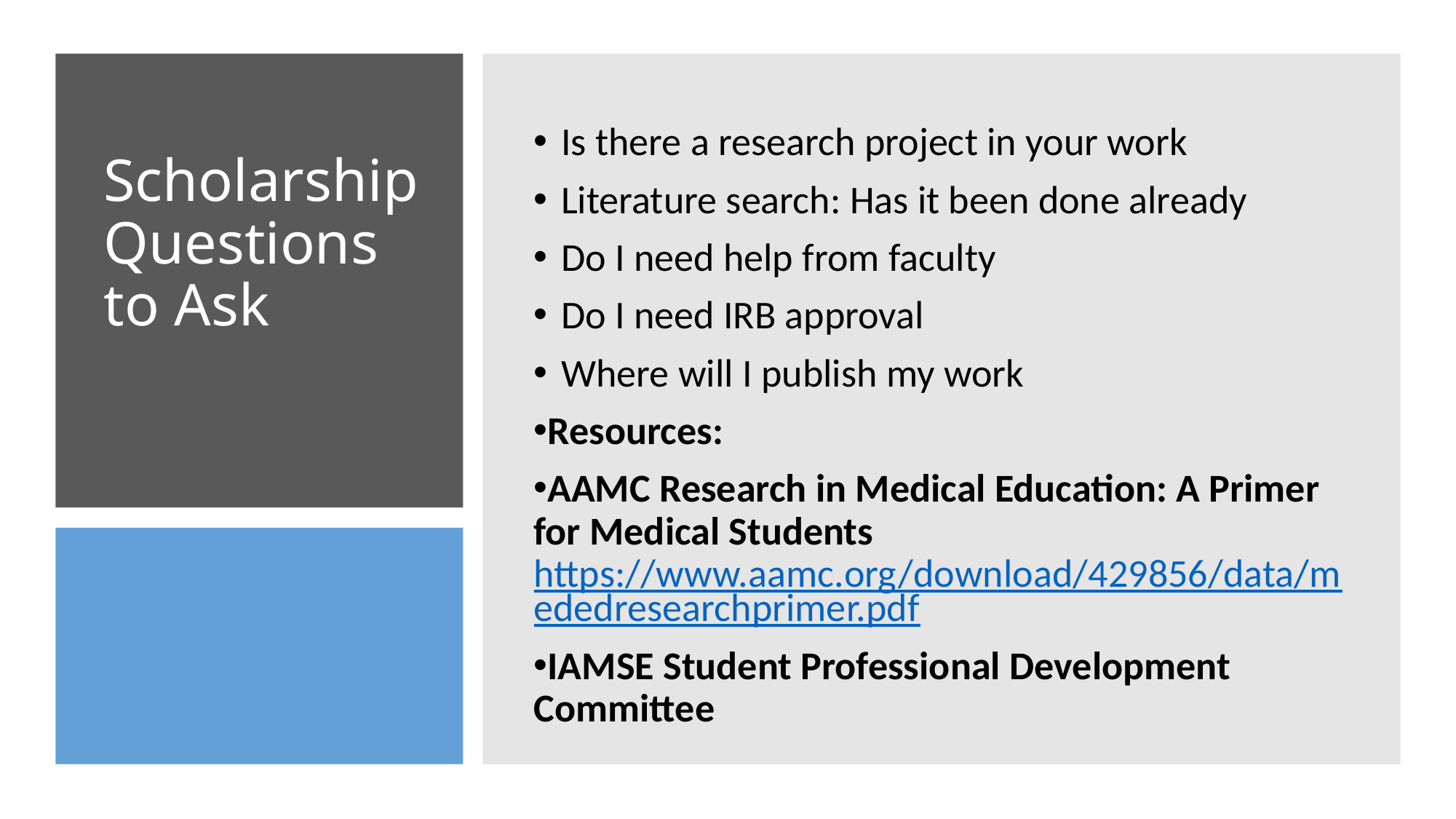

Is there a research project in your work
Literature search: Has it been done already
Do I need help from faculty
Do I need IRB approval
Where will I publish my work
Resources:
AAMC Research in Medical Education: A Primer for Medical Students https://www.aamc.org/download/429856/data/mededresearchprimer.pdf
IAMSE Student Professional Development Committee
Scholarship Questions to Ask

## Slide 44
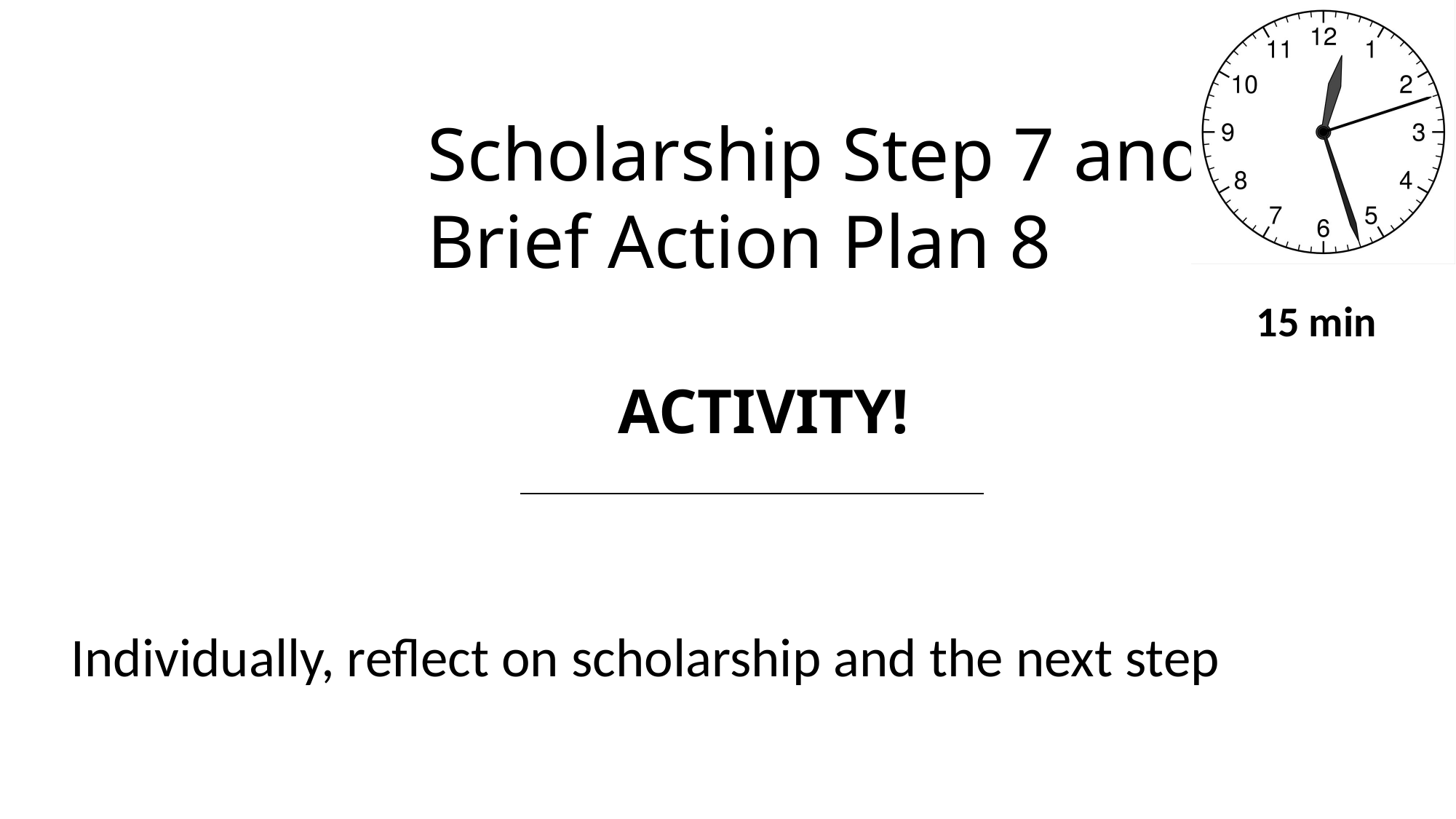

Scholarship Step 7 and
Brief Action Plan 8
15 min
ACTIVITY!
Individually, reflect on scholarship and the next step

## Slide 45
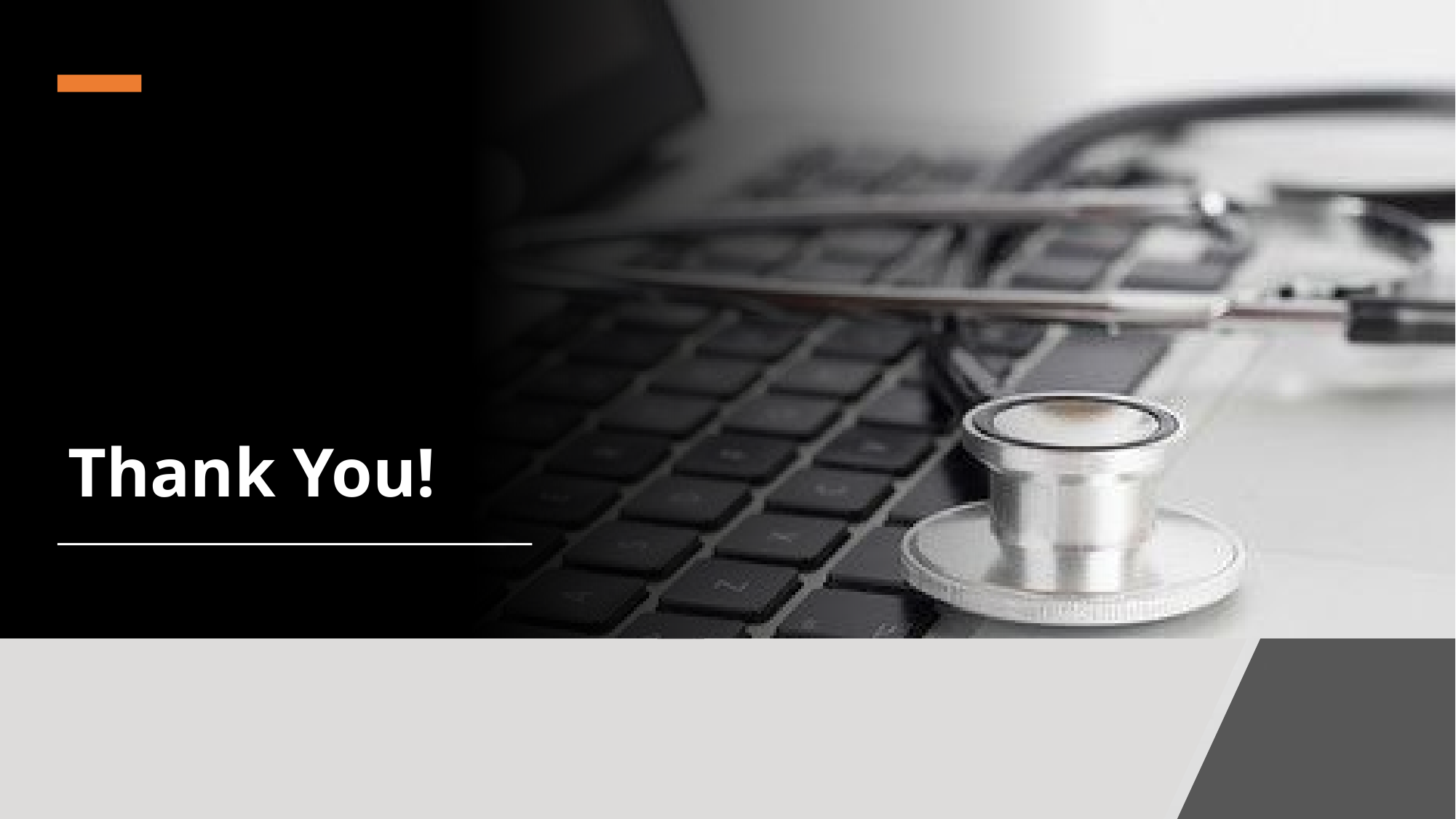

# Thank You!
