# Supplementary material for: Curriculum Design and Scholarship for New Educators: A Professional Development Workshop for Medical Students
Source: MedEdPORTAL. 2021 Apr 26;17:11130. doi: 10.15766/mep_2374-8265.11130 (PMC8071841; doi:10.15766/mep_2374-8265.11130)
Supplement: Supplementary file 1 — Workshop Agenda.docxPresentation.pptxWorksheet.docxFacilitator Notes.docxWorkshop Survey.docx [file mep_2374-8265.11130-s001.zip › C. Worksheet.docx]

| ***Curriculum*** *(Insert Catchy Title):*  **(ranging from 1 hour to several sessions)** | | |
| --- | --- | --- |
| \| **Ideas for Your Educational Planning** \| \| \| --- \| --- \| \| **1** \| **Medical Spanish** \| \| **2** \| **Addiction (Opioid) Problem** \| \| **3** \| **Wellness and Resilience** \| \| **4** \| **Disaster Preparedness** \| \| **5** \| **Pain Management** \| \| **6** \| **Maternal Fetal Health** \| \| **7** \| **LGBTQ+ Health** \| \| **8** \| ***YOUR IDEA*** \| | | |
| **STEP 1: Problem Identification and General Needs Assessment** | ***What problem needs solving? What topic do I want to teach? Why does it matter*?**  *(“COVID-19: Prepare preclinical medical students to be proactive during a natural disaster*”; *Medical Spanish:* “*Provide students with basic Spanish vocabulary for* *patient interaction*”)  **INSERT HERE** | |
| **STEP 2: Targeted Needs Assessment:** | ***Who are my learners? What setting works best for the learners and the content? What resources do I need?***  *(“3^rd^ and 4^th^ year medical students in small groups; roleplay; students a provided with cases on video and roleplay”)*  **INSERT HERE** | |
| 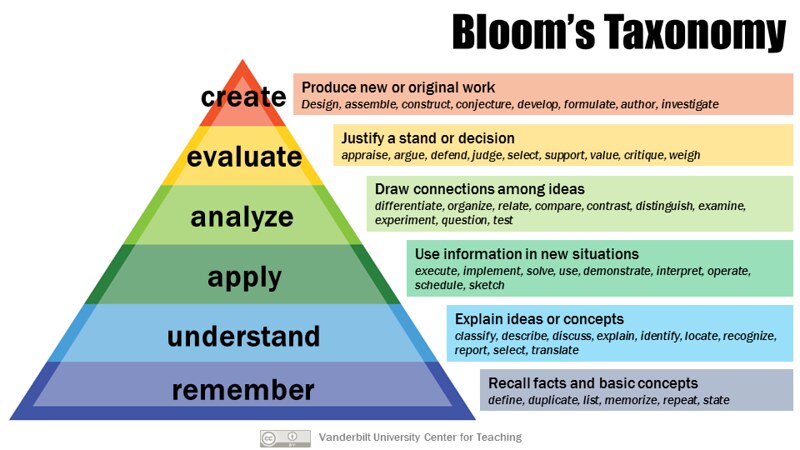  Image **Bloom's Taxonomy** Vanderbilt University Center for Teaching <http://cft.vanderbilt.edu/>, retrieved from: [https://www.flickr.com](https://www.flickr.com/photos/vandycft/29428436431) on July 06, 2020. No changes were made. Creative Commons License associated: Attribution 2.0 Generic (CC BY 2.0) <https://creativecommons.org/licenses/by/2.0/> [Photo: https://www.flickr.com/photos/vandycft/29428436431/in/photolist-LQuqT2](https://www.flickr.com/photos/vandycft/29428436431/in/photolist-LQuqT2) | | |
| **STEP3: Goal: Broad outcome statement for topic:**  *(“To demonstrate the ability triage in a disaster situation”)*  *(“To possess and apply basic vocabulary to provide nutritional counsel to outpatients in Spanish”, …..)* | | ***INSERT HERE:*** |
| **STEP3: Learning Objectives (SMART):**  ***What knowledge do I want my learners to learn*?** | | **Knowledge (cognitive):**  *(“Describe the indications for prescribing opiates”)* |

**SMART LEARNING OBJECTIVES:
S**pecific**, M**easurable**, A**ttainable**, R**esults-focused and **R**elevant**, T**ime-focused**/T**argeted

Additional space:

**INSERT HERE**: After the session, learners will be able to:

| **STEP 4: Educational Strategies** | ***How am I going to teach my learners? What works best for them, e.g., group discussion; webinars; training; simulation; ….***  *(“Standardized patient experience”; “small group role-play with script”; Large group lecture”; “a panel of pts and providers”)*  **INSERT HERE** |
| --- | --- |
| **STEP 5: Implementation** | ***What resources do I need for my session, e.g., a classroom, a projector, block of two hours…***  *(“manpower – pts/providers”; “lecture hall and breakout rooms”; “approval from dean”)*  **INSERT HERE** |
| **STEP 6: Learner Assessment and Program Evaluation** | ***Did my students learn what I intended?***  **Individual Learner Assessment**  **Formative:** *(“open discussion about how it went”)*  **INSERT HERE**  **Summative:** *(“written graded test”)*  **INSERT HERE** |
|  | **Program Evaluation (in broad strokes):**  ***Did they enjoy it and are they able to apply it?***  *(“the session was applicable for me”; “I am comfortable to prescribe …..” )*  **INSERT HERE** |
| **STEP 7: Scholarship plan and Dissemination plan Challenge**  Take a moment and reflect on the following,   1. **What is your research question?** 2. **How could you disseminate this novel session/program?** 3. **What is your final product (journal article or MedEdPORTAL submission)?** 4. **What journal could you publish in?** 5. **If an article, what types of article?** 6. **…………?** | |
| **STEP 8: Action Items: What are you going to do on Monday?** | |
