# Supplementary material for: Curriculum Design and Scholarship for New Educators: A Professional Development Workshop for Medical Students
Source: MedEdPORTAL. 2021 Apr 26;17:11130. doi: 10.15766/mep_2374-8265.11130 (PMC8071841; doi:10.15766/mep_2374-8265.11130)
Supplement: Supplementary file 1 — Workshop Agenda.docxPresentation.pptxWorksheet.docxFacilitator Notes.docxWorkshop Survey.docx [file mep_2374-8265.11130-s001.zip › D. Facilitator Notes.docx]

**Facilitator Notes: Curriculum Design and Scholarship for New Educators:
A Professional Development Workshop for Medical Students**

**1) Slide 1: Facilitator Introduction.**

All facilitators introduce themselves.

-----

The title slide contains an image showing a **Stethoscope on laptop keyboard**, retrieved from: www.flickr.com on July 12, 2020 by Marco Verch (<https://foto.wuestenigel.com/>), Creative Commons 2.0. (<https://creativecommons.org/licenses/by/2.0/>)

Stethoscope on laptop keyboard: <https://foto.wuestenigel.com/stethoscope-on-laptop-keyboard/>

Photo: <a href="https://foto.wuestenigel.com/stethoscope-on-laptop-keyboard/" target="_blank">Stethoscope on laptop keyboard</a> by <a href="https://www.plaghunter.com/marco-verch/" target="_blank">Marco Verch</a> under <a href="https://creativecommons.org/licenses/by/2.0/" target="_blank">Creative Commons 2.0</a>

**2) Slide 2: Workshop Objectives.**

Facilitators convey the workshop objectives. By the end of this activity, learners will be able to:

1. Apply principles of the generally accepted six-step approach for curriculum development for health professions education
2. Describe characteristics of adult learners
3. Develop SMART Learning Objectives
4. Describe educational strategies considered to promote active learning
5. Identify factors influencing the implementation of health professions curricula
6. Differentiate assessment and evaluation
7. Describe foundations of educational scholarship

**3) Slide 3: Workshop Instructions.**

This slide includes the instructions for the remote workshop. This helps establishing framework for the workshop and is also a good time to ask for questions and let the participants know how you're going to answer them. In the remote workshop students could ask questions through the chat box, which was monitored by the facilitators.

We provide the instructions for the interactive program:

“In this workshop In this workshop you will create a short curriculum. This is a tall order on zoom, but we provide a worksheet with guiding questions for help. We will use break out groups where you develop steps of a curriculum with your group. Every breakout group is limited to 8 members and is assigned a number. A Google doc worksheet is associated with each individual workout group and has the same number. In the chat box below we will place the link for all the Google docs. Please open it prior to entering your breakout room so you can then choose the right number that is the same as your breakout group. We will call upon a group for discussion. Please keep yourself muted until ready to speak”.

**4) Slide 4: Section Slide.**

This slide is the lead-over to the role of students as active collaborators and leaders in medical education.

-----

The image shows a **Stethoscope on laptop keyboard**, retrieved from: www.flickr.com on July 12, 2020 by Marco Verch (<https://foto.wuestenigel.com/>), Creative Commons 2.0. (<https://creativecommons.org/licenses/by/2.0/>)

Stethoscope on laptop keyboard: <https://foto.wuestenigel.com/stethoscope-on-laptop-keyboard/>

Photo: <a href="https://foto.wuestenigel.com/stethoscope-on-laptop-keyboard/" target="_blank">Stethoscope on laptop keyboard</a> by <a href="https://www.plaghunter.com/marco-verch/" target="_blank">Marco Verch</a> under <a href="https://creativecommons.org/licenses/by/2.0/" target="_blank">Creative Commons 2.0</a>

**5) Slide 5: Who knows the curriculum best? Students as stakeholders know the curriculum best.**

This slide is the lead-over to the role of students as active collaborators and leaders in medical education. Students as the critical consumers-turned-stakeholders of education understand the impact of education best.

-----

Image by author Elisabeth Schlegel showing a word cloud generated using [wordart.com](https://nam04.safelinks.protection.outlook.com/?url=http%3A%2F%2Fwordart.com%2F&data=02%7C01%7CElisabeth.Schlegel%40hofstra.edu%7Cb992dfa949df433d867808d825c521e2%7Ce32fc43d7c6246d9b49fcd53ba8d9424%7C0%7C0%7C637300876931360147&sdata=cRS5RDdiqDlti2uxapneM97lTKIIvJD9EjA%2B9KhWviQ%3D&reserved=0).

**6) Slide 6: Reflexions of an MD on past curricular experience. Where did I need help? How did I know my Perspectives were valued?**

This slide is the lead-over to the role of students as active collaborators and leaders in medical education.

Students as the critical consumers-turned-stakeholders of education understand the impact of education best.

This is a good point to pause and ask the students in the room about their prior experiences in curricular decision-making:

Has anyone been involved in curriculum development?

Can you describe how curriculum content and changes are decided for each course at your institution?

**7) Slide 7: Flow of Communication and Collaborating Committees Governing Curricular Changes. Advocacy while Navigating the Politics of Curriculum Change**

This is another personal reflection on past curricular decision making. How did advocacy work?

This is a good point to pause and ask the students in the room about their prior experiences.

This is also a good place to highlight the complexities of the process for curriculum design and change.

**8) Slide 8: Increasingly, there are Resources for Curricular Decisions and Scholarship Available. Referral to established Health Professions Educations Resources. Growing Volume of online/outside Resources.**

Yun: [There are great resources available for curricular decisions. Aside from international conferences and associations such as the Association for Medical Education in Europe (AMEE) or the International Association of Medical Science Educators (IAMSE), There are important publications such as curriculum development for medical education By David Kern and colleagues, or Scholarship Assessed by Charles Glassick.]

Reference:

- Glassick, C. E., Huber, M. T., & Maeroff, G. I. (1997). *Scholarship Assessed: Evaluation of the Professoriate. Special Report*. Jossey Bass Inc., Publishers, 989 Market Street, San Francisco, CA 94103.

**9) Slide 9: Section Slide**.

This slide is the lead-over to the Principles of program development.

Yun: [This is another personal reflection on past curricular decision making. How did advocacy work?]
This is another good point to pause and ask the students in the room about their prior experiences.

The image shows a **Stethoscope on laptop keyboard**, retrieved from: www.flickr.com on July 12, 2020 by Marco Verch (<https://foto.wuestenigel.com/>), Creative Commons 2.0. (<https://creativecommons.org/licenses/by/2.0/>)

Stethoscope on laptop keyboard: <https://foto.wuestenigel.com/stethoscope-on-laptop-keyboard/>

Photo: <a href="https://foto.wuestenigel.com/stethoscope-on-laptop-keyboard/" target="_blank">Stethoscope on laptop keyboard</a> by <a href="https://www.plaghunter.com/marco-verch/" target="_blank">Marco Verch</a> under <a href="https://creativecommons.org/licenses/by/2.0/" target="_blank">Creative Commons 2.0</a>

**10) Slide 10: Behind complex Curricula are Connecting Assumptions. …Medical School Curricula Look Complicated…..**

Yun: [Hidden behind this curriculum map are several assumptions commonly shared by medical schools:

1. Educational programs have goals, objectives, and an educational philosophy
2. Medical education has a professional and ethical obligation to meet the needs of learners, patients, and society
3. Medical educators should be held accountable for the outcomes of their interventions
4. A logical, systematic approach to curriculum development will help achieve these ends. There is such a system in place, and it works worldwide in the same way.

Reference:

- Thomas, P. A., Kern, D. E., Hughes, M. T., & Chen, B. Y. (Eds.). (2016). Curriculum development for medical education: a six-step approach. JHU Press.

**11) Slide 11: Kern’s Six-Step Approach.**

Here we introduce Kern’s Six Step approach:

“Kern’s Six Step approach, published in “Curriculum Development for Medical Education”, now it is 3^rd^ edition, proposes such a system.

By breaking down program development into six steps, curriculum development becomes feasible. Please note that such an approach can be used to create workshops, courses or an individual session.

These steps included are, (1) problem identification & general needs assessment; (2) targeted needs assessment; (3) goals and objectives; (4) educational strategies; (5) implementation; and (6) evaluation and feedback.

We added two more steps, (7) scholarship; and (8), and Action Plan: What is your next step that you will be doing different on Monday?”

Image of book cover retrieved from <https://books.google.com/>on July 6, 2020. The image is in the public domain.

**12) Slide 12: Application of Kern’s Six-Step Approach.**

Here we introduce an example of an application of Kern’s Six Step approach.

A previous MS4 Student, now a resident developed a training session for medical students becoming HIV Educators.

His problem identification was breaking down the barriers to provide pre and post exposure HIV prophylaxis to MSM patients.

His learners were year one and two medical students; his goals were describing the indications for prescribing prep and pep reflecting and addressing concerns regarding providing preventative care for high risk populations.

As educational strategies he chose to give the students pre-work, to study the prep action kits prior to the session. In class, Josh provided a 15-minute large group session, small group breakout groups and standardize patience and reflection. His implementation included approval by the dean, from the faculty, and negotiating curricular time.
Evaluation and feedback included feedback from the standardized patience, self-reflection of the students and a session evaluation survey.

Background reflecting societal needs:

MSM patients represent 70% of new HIV diagnoses per year. Despite this heavy disease burden, studies have indicated that multiple factors conspire to make providers less efficient or willing to prescribe pre-exposure prophylaxis (PrEP) or post-exposure prophylaxis (PEP), both effective tools for preventing the spread of HIV.

An intervention is needed at multiple stages of medical education in order to help learners identify their own misconceptions and potential barriers to care for this patient population.

**13) Slide 13: Relate to Kern’s Six-Step Approach. Why follow these Steps…?**

Here we introduce the rationale of applying Kern’s Six Step approach.

Yun/Elisabeth: [From planning a medical program to a brief help-line curriculum, these steps and considerations are best practices. Please note, that due to the nature of the expanding knowledge of teaching and planning medical programs, these considerations represent the bare minimum. Additional directions and requirements will be necessary depending on the framework, such as residency training and competencies which depend on accrediting bodies. Considering implementing training in an institution, this structure will have to be rigidly connected to institutional outcomes.

These steps are universally applied and help securing faculty support but also negotiate overcoming barriers and limited resources. Using these steps you demonstrate that you're prepared to lead change because you have the knowledge to fill a gap in the needs of the patient care, for medical practitioners or the society

**14) Slide 14: Introduction of the Worksheet. Instructions: Develop a Curriculum.**

Your instructions for the day are to develop a curriculum in your breakout group. Each breakout group chooses a topic for a curriculum to work on using the question driven template. We will work through the steps in sections, but I suggest you select a group leader or scribe for completing the sections. After the time is up groups are called on to report which also means that we will reveal the Google doc.

**15) Slide 15: Ideas for Planning of your Educational Intervention.**

The table shows a list of topics which are also included in the Google doc. Students are encouraged to develop their own ideas.

**16) Slide 16: Section Slide.**

This slide is the lead-over to Curriculum design, Kern’s steps one and two.

The image shows a **Stethoscope on laptop keyboard**, retrieved from: www.flickr.com on July 12, 2020 by Marco Verch (<https://foto.wuestenigel.com/>), Creative Commons 2.0. (<https://creativecommons.org/licenses/by/2.0/>)

Stethoscope on laptop keyboard: <https://foto.wuestenigel.com/stethoscope-on-laptop-keyboard/>

Photo: <a href="https://foto.wuestenigel.com/stethoscope-on-laptop-keyboard/" target="_blank">Stethoscope on laptop keyboard</a> by <a href="https://www.plaghunter.com/marco-verch/" target="_blank">Marco Verch</a> under <a href="https://creativecommons.org/licenses/by/2.0/" target="_blank">Creative Commons 2.0</a>

**17). Slide 17: STEP 1: Problem Identification and General Needs Assessment.**

Problem Identification and General Needs assessment

Problem identification in general needs assessment is your service to society. It means identifying the biggest problem in health care right where you currently are. That might involve several sectors of the society such as patients, health care professionals, medical educators, the society as a whole. In addition, you compared the current approach, which is currently used with the ideal approach and how health care should be addressed. This slide also defines what the general needs assessment (GNA), which is the difference between a realistic approach and an ideal approach to addressing a health care problem. After thinking about a specific problem or issue, it is then important to consider whom the topic affects.

One example, which I invented, might be: “Outpatient diabetes management in the Northville Health Eastern Region needs to be improved”. That would be a typical statement about the problem identification and general needs assessment as a first step. That is how you would phrase it.

**18). Slide 18: A Health Care Need for many.**

Whom does the problem effect?

The health care need may relate to a specific problem, such as providing care to patients infected with a rare pathogen, or it may relate to the health care needs of society in general, such as whether the access to health care is effective. As describe in Thomas and colleagues (2016), the general needs assessment is the difference between the ideal approach and the current approach.

Reference: Thomas, P. A., Kern, D. E., Hughes, M. T., & Chen, B. Y. (Eds.). (2016). *Curriculum development for medical education: a six-step approach*. JHU Press.

**Note:** One click adds a banner about the (fictional) outpatient diabetes management in the Northville health eastern region.

------

Image by author Elisabeth Schlegel showing a word cloud generated using [wordart.com](https://nam04.safelinks.protection.outlook.com/?url=http%3A%2F%2Fwordart.com%2F&data=02%7C01%7CElisabeth.Schlegel%40hofstra.edu%7Cb992dfa949df433d867808d825c521e2%7Ce32fc43d7c6246d9b49fcd53ba8d9424%7C0%7C0%7C637300876931360147&sdata=cRS5RDdiqDlti2uxapneM97lTKIIvJD9EjA%2B9KhWviQ%3D&reserved=0).

**19). Slide 19: Targeted Needs Assessment**

This slide is a lead over to the following questions:

- Who are my learners?
- What setting works best for the learners and the content?
- What resources do I need?

**20). Slide 20: Targeted Needs Assessment.**

The targeted needs assessment means identifying the students or learners.

You ask yourself, who is your intended audience? And what does the learner need to be successful? You might have to think about previous training and experience, or the type of organization knowledge and skills.

Consider:

- Previous training and experiences
- Type of Organization
- Key considerations
- Existing proficiencies & barriers
- Expectations of knowledge/skills
- Attitudes about curricular topic
- Learning styles, preferred methods

And I believe this is just the right moment to talk about how adults learn (as opposed to children).

------

Image by author Elisabeth Schlegel showing a word cloud generated using [wordart.com](https://nam04.safelinks.protection.outlook.com/?url=http%3A%2F%2Fwordart.com%2F&data=02%7C01%7CElisabeth.Schlegel%40hofstra.edu%7Cb992dfa949df433d867808d825c521e2%7Ce32fc43d7c6246d9b49fcd53ba8d9424%7C0%7C0%7C637300876931360147&sdata=cRS5RDdiqDlti2uxapneM97lTKIIvJD9EjA%2B9KhWviQ%3D&reserved=0).

**21). Slide 21: The targeted needs Assessment means identifying the Learners. Adults Learn Differently!**

The targeted needs assessment means identifying the students or learners. This is a good time to take a step back and reflect about our own learning experiences throughout our careers.

Adult learners learn completely different as compared to children: Think about yourself as a learner. We need to be respected and be perceived as self-directed and self-motivated. We bring our own life experiences. As adult learners we are practical and want to include our previous knowledge into a learning situation or the immediate workplace.

There might be resistance to change, since adult learners already established their learning preferences. Consider this when you describe your learners.

A few Highlights from Adult Learning Theory:

Adults require:

- A save learning environment
- Feel save to disagree and ask “naïve” questions
- Raise opposing ideas
- Take time to reflect & comprehend
- Thoughtful choice of Active Learning

Reference:

- Knowles, M. S., Holton, E., & Swanson, R. A. (1973). The adult learner. *A Neglected Species. Houston, ss*, *104*.
- Knowles, M. S. (1984). Introduction: the art and science of helping adult learn. Andragogy in action: applying modern principles of adult learning.

**22). Slide 22: Activity: Complete the Google doc template sections steps 1 and 2**

Now we will break out into break out rooms where you develop steps of a curriculum with your group. Remember, every breakout room is assigned a number. A Google doc is associated with each individual breakout group/room and has the same number.

In the chat box below we will place the link for all the Google docs.

Please open it prior to entering your breakout room so you can then choose the right number that is the same as your breakout group.

*Question 1: What problem do I want to solve? Write a statement about what needs to change.*

*Question 2: Who are my learners? What setting works best for the learners and the content? What resources do I need?*

*(“3^rd^ and 4^th^ year medical students in small groups; roleplay; students a provided with cases on video and roleplay”)*

**23). Slide 23: Section Slide.**

This slide is the lead-over to Kern’s step three.

The image shows a **Stethoscope on laptop keyboard**, retrieved from: www.flickr.com on July 12, 2020 by Marco Verch (<https://foto.wuestenigel.com/>), Creative Commons 2.0. (<https://creativecommons.org/licenses/by/2.0/>)

Stethoscope on laptop keyboard: <https://foto.wuestenigel.com/stethoscope-on-laptop-keyboard/>

Photo: <a href="https://foto.wuestenigel.com/stethoscope-on-laptop-keyboard/" target="_blank">Stethoscope on laptop keyboard</a> by <a href="https://www.plaghunter.com/marco-verch/" target="_blank">Marco Verch</a> under <a href="https://creativecommons.org/licenses/by/2.0/" target="_blank">Creative Commons 2.0</a>

**24). Slide 24: Goals and Objectives**

After the needs of targeted learners have been determined, goals and objectives for the curriculum can be developed. Goals and objectives allow communication of what the curriculum is about to others and provide a basis for assessments and evaluation. Goals are broad outcome statement of knowledge (K), skills (S) and attitudes (A) students which students will possess upon completion of the educational program. Similarly, objectives may include cognitive (knowledge), affective (attitudinal), or psychomotor (skill and behavioral) objectives for the learner. Importantly, objectives, educational methods (Step 4), and assessment and evaluation have to be aligned provide guidance for educators' efforts to facilitate students' progress toward desired academic outcomes.

- Roach, A. T., Niebling, B. C., & Kurz, A. (2008). Evaluating the alignment among curriculum, instruction, and assessments: Implications and applications for research and practice. Psychology in the Schools, 45(2), 158-176.
- Thomas, P. A., Kern, D. E., Hughes, M. T., & Chen, B. Y. (Eds.). (2016). Curriculum development for medical education: a six-step approach. JHU Press.

**25). Slide 25: Writing SMART Learning Objectives**

A learning objective is a description of what the learner will be able to do after completion of an educational activity. It specifies the knowledge (K), skills (S) and/or attitude (A) the learners will gain from the educational activity in a measurable manner. Using action verbs, it is best practice to formulate learning objectives as SMART learning objectives. This five elements include who, will do, how much or how well, of what, by when. Start formulating learning objectives “After the session, students will be able to…”, abbreviated LWBAT.

- 1. **S**pecific
  2. **M**easurable/Observable
  3. **A**ttainable for target audience within scheduled time and specified conditions
  4. **R**elevant and **R**esults-Focused
  5. **T**ime-Focused/**T**argeted to the learner and to the desired level of learning
- Chatterjee, D., & Corral, J. (2017). How to write well-defined learning objectives. The journal of education in perioperative medicine: JEPM, 19(4).
- Thomas, P. A., Kern, D. E., Hughes, M. T., & Chen, B. Y. (Eds.). (2016). Curriculum development for medical education: a six-step approach. JHU Press.

**26). Slide 26: Learning Objectives for Each Domain**

Learning objectives relate to learning in the cognitive, affective, and psychomotor domains.

- Learning objectives pertaining to the cognitive domain of learning are often referred to as “knowledge” objectives.
- Learning objectives pertaining to the affective domain are also termed as “attitudinal” objectives, referring to specific attitudes, values, beliefs, biases, emotions, or role expectations that can affect a learner’s learning or performance. Affective learning objectives are usually more difficult to formulate and to measure than cognitive objectives. Observation or review of reflective work are choices to evaluate attitudes.
- Learning objectives pertaining to the psychomotor domain of learning are often termed “skill” or “behavioral” objectives and refer to specific psychomotor tasks or actions that may involve hand or body movements, vision, hearing, speech, or the sense of touch. Medical interviewing, patient education and counseling, interpersonal communication, physical examination, record keeping, and procedural skills pertain to the psychomotor domain.

Anderson and Krathwohl revised these categories by aligning with cognitive psychology and understanding of learning.

- Anderson, L. W. (2001). Krathwohl (Eds.). A Taxonomy for learning, teaching, and assessing: A revision of Bloom's taxonomy of educational objectives.
- Bloom, B. S. (1984). Taxonomy of Educational Objectives, Book 1 Cognitive Domain (original publication 1956).
- Mager RF. Preparing Instructional Objectives: A Critical Tool in the Development of Effective Instruction, 3rd. ed. Atlanta: CEP Press; 1997. Pp. 151– 54.
- Thomas, P. A., Kern, D. E., Hughes, M. T., & Chen, B. Y. (Eds.). (2016). Curriculum development for medical education: a six-step approach. JHU Press.

**27). Slide 27: Cognitive Verbs, Bloom’s Taxonomy.**

Verbs related to Bloom’s Taxonomy allow writing concise learning objectives. The revised taxonomy published in 2001 addresses the categories and associated action words that allow describing distinct processes which can be tested.

**Note:** The image is also provided in the worksheet.

------

Image by Vanderbilt University Center for Teaching, retrieved from: [https://www.flickr.com](https://www.flickr.com/photos/vandycft/29428436431) on July 06, 2020. No changes were made. Creative Commons License associated: Attribution 2.0 Generic (CC BY 2.0)

**28). Slide 28. Developing Cognitive Learning Objectives.**

Complete the sections of the worksheet pertaining to Step 3: Determine the goals and learning objectives of your educational innovation.

Return to your breakout rooms. In your groups discuss the following:

*1. Determine the broad outcome statement for topic.*

*2. Determine the learning objectives.*

**Note:** Prompts for completing the assignment are included in the worksheet. The fields were to insert the goals and learning objectives are clearly marked. The time can be adjusted to the pace of the learners.

The remote workshop discussed cognitive learning objectives. A verb sheet (Appendix D) is available to develop learning objectives for all domains if desired.

------

Image of the clock[: "File:AnalogClockAnimation1 still frame.svg"](https://commons.wikimedia.org/w/index.php?curid=58350332) by [Jahobr](https://commons.wikimedia.org/wiki/User:Jahobr) is licensed under [CC0 1.0](http://creativecommons.org/publicdomain/zero/1.0/deed.en?ref=ccsearch&atype=rich)

**29). Slide 29: Section Slide.**

This slide is the lead-over to Kern’s steps four and five.

The image shows a **Stethoscope on laptop keyboard**, retrieved from: www.flickr.com on July 12, 2020 by Marco Verch (<https://foto.wuestenigel.com/>), Creative Commons 2.0. (<https://creativecommons.org/licenses/by/2.0/>)

Stethoscope on laptop keyboard: <https://foto.wuestenigel.com/stethoscope-on-laptop-keyboard/>

Photo: <a href="https://foto.wuestenigel.com/stethoscope-on-laptop-keyboard/" target="_blank">Stethoscope on laptop keyboard</a> by <a href="https://www.plaghunter.com/marco-verch/" target="_blank">Marco Verch</a> under <a href="https://creativecommons.org/licenses/by/2.0/" target="_blank">Creative Commons 2.0</a>

**30). Slide 30: Educational Strategies and Implementation: STEP 4 and STEP 5**

This is another point to make a pause and reflection on past educational experiences: How did we learn prior to medical school, and how has our learning changed?

This is the time when you have to think about the ***how*** *are you going to teach* ***what*** *to the learners you described* previously in the targeted needs assessment. Now you have to provide educational strategies and methods that ensure your learners can master your learning objectives and the content you set out to be covered.

Educational strategies might include lectures or facilitated small groups, or perhaps include a flipped-classroom design including pre-work. Based on how you teach, you need to think about the resources, support, and approvals you need for implementation.

More Info:

“Bonwell (1991) *"states that in active learning, students participate in the process and students participate when they are doing something besides passively listening.”*

*Active learning is "a method of learning in which students are actively or experientially involved in the learning process and where there are different levels of active learning, depending on student involvement.”*

Reference:

Bonwell, C. C., & Eison, J. A. (1991). *Active Learning: Creating Excitement in the Classroom. 1991 ASHE-ERIC Higher Education Reports*. ERIC Clearinghouse on Higher Education, The George Washington University, One Dupont Circle, Suite 630, Washington, DC 20036-1183.

**31). Slide 31: Considerations for Settings for Educational Strategies**

This gets into the nitty-gritty. Educational strategies involve space, faculty, support staff, and other resources. Ideally, you aim for implement active learning, but it depends on the type of learners, your timeframe, and your resources.

As an example, while small groups allow experiential learning through several new methodologies (PBL; TBL; Hybrid Case-based/Problem-based), many small spaces are needed. Lectures allow to reach a large audience which saves money and resources but need faculty development to enable active learning.

Some methods need careful capital building planning, since they require either multiple small rooms or specific classroom settings. **The question often is, what is the most that can be accomplished, given any resource limitations?**

**32). Slide 32: Step 5: Implementation**

This is another point to make a pause and reflect on the ideas about educational methods.

Space needs are only one aspect of the resources required for implementation.

Implementation is the moment when you plan very carefully to identify the resources that you need, such as faculty, IT, administration, or stakeholders, but also resources such as time.

You must make an inventory of the facilities, resources, support personnel, …. you might need. In addition, this is also the time to garner support and identify barriers in order to overcome them, and you have to start PR for introducing your new curriculum.

**33). Slide 33: Active Pedagogy Strategies (STEP 4) and STEP 5, Implementation**

Circling back to active learning, here we see a few of the most effective active learning strategies (STEP 4) with the resources necessary (STEP 5).

Please note, active learning strategies require faculty development, and all of them require an appropriate physical space.

Many methodologies are not resource-intense: Simple polls can be done on ZOOM, and worksheets work quite well no matter if they are physical or if they are Google docs that invite collaboration. Sometimes one must be inventive; if a lecture hall is all there is, then panel discussions are exciting ways to expose students to experts and lead to spirited discussions. However, some of these methods require a lot of resources and strict protocols such as, e.g., simulation.

**34). Slide 34: Activity: Develop Educational Strategies (STEP 4) & Implementation (STEP 5)**

Complete the sections of the worksheet pertaining to Steps 4 and 5.

The questions and examples in the worksheet will guide you: *How am I going to teach my learners? What works best for them, e.g., group discussion; webinars; training; simulation; ….(“Standardized patient experience”; “small group role-play with script”; Large group lecture”; “a panel of pts. and providers”).*

You need discuss with your peers about the resources you need for implementing your program: *What resources do I need for my session, e.g., a classroom, a projector, block of two hours…*(“manpower – pts/providers”; “lecture hall and breakout rooms”; “approval from dean”)

**35). Slide 35: Section Slide.**

Lead over to Kern’s Step 6: Assessment and Evaluation.

The image shows a **Stethoscope on laptop keyboard**, retrieved from: www.flickr.com on July 12, 2020 by Marco Verch (<https://foto.wuestenigel.com/>), Creative Commons 2.0. (<https://creativecommons.org/licenses/by/2.0/>)

Stethoscope on laptop keyboard: <https://foto.wuestenigel.com/stethoscope-on-laptop-keyboard/>

Photo: <a href="https://foto.wuestenigel.com/stethoscope-on-laptop-keyboard/" target="_blank">Stethoscope on laptop keyboard</a> by <a href="https://www.plaghunter.com/marco-verch/" target="_blank">Marco Verch</a> under <a href="https://creativecommons.org/licenses/by/2.0/" target="_blank">Creative Commons 2.0</a>

**36). Slide 36: Kern’s Step 6: Assessment and Evaluation (incl. Feedback.**

Step 6 has several components; it includes assessment of the performance of individuals (hence: individual assessment) as well as evaluation of the curriculum (usually termed “program evaluation”). Assessment and evaluation may be formative (including ongoing feedback to the learners for improvement) or summative (providing a final “grade”)

Evaluation usually collects data needed to drive the improvement of a curriculum but also to gain support and resources. Most often, evaluation is needed to answer questions about the effectiveness of a curriculum.

Thomas, P. A., Kern, D. E., Hughes, M. T., & Chen, B. Y. (Eds.). (2016). *Curriculum development for medical education: a six-step approach*. JHU Press.

**37). Slide 37: Formative and Summative Assessment**

It is especially important to provide feedback to the learner. With formative assessment, there is ongoing communication between teacher and learner. The goal of formative assessment is to monitor student learning for ongoing improvement. Summative assessment, on the other hand, will lead to a final grade, which is usually compared against a standard or benchmark. There are important questions to ask such as why assess the student; or who should be doing the assessment. The clearer the assessment the more informative the data gained.

Reference:

Harden, R. M., Laidlaw, J. M., & Mmed, D. (2020). *Essential skills for a medical teacher: an introduction to teaching and learning in medicine*. Elsevier.

**38). Slide 38: Evaluation**

Curriculum evaluation is the final part of an educational process, and essential to ongoing improvement. Ideally, a curriculum is going through several cycles, with improvement during each iteration. The focus is on quality improvement not on students’ performance.

Reference:

Harden, R. M., Laidlaw, J. M., & Mmed, D. (2020). *Essential skills for a medical teacher: an introduction to teaching and learning in medicine*. Elsevier.

**39). Slide 39: Kirkpatrick’s Model**

Kirkpatrick's model of program evaluation is a widely used method. There are four level that comprise this model, (1) Level 1: Reaction; (2) Level 2: Learning; (3) Level 3: Behavior; and (4) Level 4: Results.

Each level implies a more holistic point of view with the inclusion of additional stakeholders.

As an example for program evaluation of a workshop, level 1 would include satisfaction data, level 2 learning outcomes of workshop participants, level 3 participants’ ability to implement the learned in a different environment, and level 4 feedback from this environment (e.g., patients or a staff) on the new implementation. The higher the Kirkpatrick level the more difficult to obtain data. Most often, satisfaction and learner performance are the most surveyed levels of data.

Reference:

Kirkpatrick DI. Evaluating Training Programs: The Four Levels, 3rd ed. San Francisco: Berrett-Koehler; 2006.

**40). Slide 40: Activity: Complete Section STEP 6 on the Worksheet**

Develop formative and summative assessments of your learners and think about a program evaluation in broad strokes.

You could start with a satisfaction surveys as Kirkpatrick level one.

The guiding prompts and examples will help you.

**Note:** The fields were to insert the assessment/evaluation options are clearly marked. The time can be adjusted to the pace of the learners.

------

Image of the clock[: "File:AnalogClockAnimation1 still frame.svg"](https://commons.wikimedia.org/w/index.php?curid=58350332) by [Jahobr](https://commons.wikimedia.org/wiki/User:Jahobr) is licensed under [CC0 1.0](http://creativecommons.org/publicdomain/zero/1.0/deed.en?ref=ccsearch&atype=rich)

**41). Slide 41: Section Slide**

This slide is the lead-over to the next step: Scholarship.

-----

The image shows a **Stethoscope on laptop keyboard**, retrieved from: www.flickr.com on July 12, 2020 by Marco Verch (<https://foto.wuestenigel.com/>), Creative Commons 2.0. (<https://creativecommons.org/licenses/by/2.0/>)

Stethoscope on laptop keyboard: <https://foto.wuestenigel.com/stethoscope-on-laptop-keyboard/>

Photo: <a href="https://foto.wuestenigel.com/stethoscope-on-laptop-keyboard/" target="_blank">Stethoscope on laptop keyboard</a> by <a href="https://www.plaghunter.com/marco-verch/" target="_blank">Marco Verch</a> under <a href="https://creativecommons.org/licenses/by/2.0/" target="_blank">Creative Commons 2.0</a>

**Slide 42: Recap of Kern’s Six Steps, and Scholarship**

This slide is a brief summary about the process accomplished, and leads over to scholarship.

The dissemination of a curriculum or related work can be important for several reasons, such as addressing the health care problem for a given population.

In addition, academic clinician-educators may spend a lot of time on curriculum development and need to publish their work in order to make it count towards a career path. Scholarly work is judged by the degree to which the work has been disseminated and had an impact at a local, regional, national, or international level.

Scholarship of a medical program is related to Kern’s Six steps, and planning should start prior to implementation. Especially the program evaluation provides important data to report on. Scholarship usually starts with the literature search about the field and identifying a gap of knowledge to be filled. Next, a journal to publish in should be identified in order to format the information from the get-go.

An important clearinghouse and journal for medical education is the MedEdPORTAL. This database includes a wide variety of educational documents and materials that have been prepared by educators from many institutions.

References:

- Association of American Medical Colleges. MedEdPORTAL [Internet]. Available at www.mededportal.org.
- Thomas, P. A., Kern, D. E., Hughes, M. T., & Chen, B. Y. (Eds.). (2016). Curriculum development for medical education: a six-step approach. JHU Press.

**43). Slide 43: Scholarship Questions to Ask**

This slide is meant to stimulate a plenary discussion, and individual participants may have different needs. The facilitators may point out resources to the students such as, e.g., the AAMC primer as well as organizations supporting medical education and scholarship in general.

**44). Slide 44: Activity: Complete Section STEP 7 and STEP 8 on the Worksheet**

Experience showed that this activity might rather be completed individually than in groups.

Then, facilitators may thank all participants and make themselves available for individual questions and advice.

**Note:** This exercise is a point of reflection for personal thoughts on scholarship and identification of the next step. The time can be adjusted to the pace of the learners.

------

Image of the clock[: "File:AnalogClockAnimation1 still frame.svg"](https://commons.wikimedia.org/w/index.php?curid=58350332) by [Jahobr](https://commons.wikimedia.org/wiki/User:Jahobr) is licensed under [CC0 1.0](http://creativecommons.org/publicdomain/zero/1.0/deed.en?ref=ccsearch&atype=rich)

**45). Slide 45: Thank you!**

The image shows a **Stethoscope on laptop keyboard**, retrieved from: www.flickr.com on July 12, 2020 by Marco Verch (<https://foto.wuestenigel.com/>), Creative Commons 2.0. (<https://creativecommons.org/licenses/by/2.0/>)

Stethoscope on laptop keyboard: <https://foto.wuestenigel.com/stethoscope-on-laptop-keyboard/>

Photo: <a href="https://foto.wuestenigel.com/stethoscope-on-laptop-keyboard/" target="_blank">Stethoscope on laptop keyboard</a> by <a href="https://www.plaghunter.com/marco-verch/" target="_blank">Marco Verch</a> under <a href="https://creativecommons.org/licenses/by/2.0/" target="_blank">Creative Commons 2.0</a>
