# Supplementary material for: Curriculum Design and Scholarship for New Educators: A Professional Development Workshop for Medical Students
Source: MedEdPORTAL. 2021 Apr 26;17:11130. doi: 10.15766/mep_2374-8265.11130 (PMC8071841; doi:10.15766/mep_2374-8265.11130)
Supplement: Supplementary file 1 — Workshop Agenda.docxPresentation.pptxWorksheet.docxFacilitator Notes.docxWorkshop Survey.docx [file mep_2374-8265.11130-s001.zip › E. Workshop Survey.docx]

**Appendix E: Workshop Survey**

**Insert institutional Consent here: […]**

**Statement of Consent:** By filling out the survey you consent to your responses being used in the study.

**Demographics:**

Participants:

- MS1
- MS2
- MS3
- MS4
- Resident
- Basic Sciences Teaching Faculty
- Clinical Sciences Teaching Faculty
- Other ________________________________________________

**Narrative Questions**

1) What are the key messages/points you will take away from the workshop?

2) What other points would have you like to have covered?

3) Propose why knowledge of a formalized curriculum development process is important to your practice as an educator.

**Workshop Survey: Experience and Logistics**

**Please answer the following questions regarding your experience with the workshop.**

|  | **Strongly Disagree** | **Disagree** | **Neither Agree nor Disagree** | **Agree** | **Strongly Agree** |
| --- | --- | --- | --- | --- | --- |
| 1) The workshop format was enjoyable |  |  |  |  |  |
| 2) The online format of the workshop worked well |  |  |  |  |  |
| 3) The workshop was well-organized |  |  |  |  |  |
| 4) Working in breakout groups during the workshop facilitated learning |  |  |  |  |  |
| 5) The pacing of the workshop effectively facilitated my learning |  |  |  |  |  |
| 6) Overall, the workshop session met my expectations |  |  |  |  |  |

**Retrospective Pre-Post Survey: Knowledge Acquisition**

Dear participants, we are assessing your **knowledge acquisition** from the workshop. Please rate your ability to accomplish each objective prior and after the workshop.

|  | Rating **Pre**-Workshop | | | | Rating **Post**-Workshop | | | |
| --- | --- | --- | --- | --- | --- | --- | --- | --- |
|  | No Knowledge | Slightly Knowledgeable | Knowledgeable | Very Knowledgeable | No Knowledge | Slightly Knowledgeable | Knowledgeable | Very Knowledgeable |
| 1) Ability to explain the generally accepted six-step model for curriculum development for health professions education as developed by Kern and colleagues | o | o | o | o | o | o | o | o |
| 2) Describing characteristics of adult learners | o | o | o | o | o | o | o | o |
| 3) Developing SMART Learning Objectives | o | o | o | o | o | o | o | o |
| 4) Describing educational strategies considered to promote active learning | o | o | o | o | o | o | o | o |
| 5) Identifying factors influencing the implementation of health professions curricula, including but not limited to those centered around stakeholders, sponsors, learners, educators, and learning environments | o | o | o | o | o | o | o | o |
| 6) Differentiating assessment and evaluation | o | o | o | o | o | o | o | o |
