# Supplementary material for: Curriculum Design and Scholarship for New Educators: A Professional Development Workshop for Medical Students
Source: MedEdPORTAL. 2021 Apr 26;17:11130. doi: 10.15766/mep_2374-8265.11130 (PMC8071841; doi:10.15766/mep_2374-8265.11130)
Supplement: Supplementary file 1 — Workshop Agenda.docxPresentation.pptxWorksheet.docxFacilitator Notes.docxWorkshop Survey.docx [file mep_2374-8265.11130-s001.zip › Educational_Summary_Report_11130.pdf]

MedEdPORTAL® | The AAMC Journal of Teaching and Learning Resources

Original Publication Open Access

Curriculum Design and Scholarship for New Educators: A Professional Development Workshop for Medical Students

Elisabeth F.M. Schlegel, MSc, PhD, MBA, MS (HPPL)*, Jeffrey B. Bird, MA, Christopher M. Burns, PhD, Michael Cassara, DO, MSEd, Jessica O'Neil, MD, Yun Weisholtz, MD, PhD, Tao T. Le, MD, MHS

*Corresponding author: Elisabeth.Schlegel@Hofstra.edu

Abstract

Introduction: Medical students' professional development includes their role as educators. Despite greater opportunities to join medical education curriculum development, medical students' engagement in these activities remains limited. A recent national study on student leadership in curricular change revealed a formal lack of leadership and training in medical education as significant barriers. Medical students' unawareness of how to disseminate curricula as educational scholarship and its value to their careers also restricts the fullness of their formation as educators. **Methods:** We designed a 3-hour, interactive, project-focused conference workshop for medical students without prior knowledge in curriculum development. Of participants, 64 worked in 10 groups creating medical curricula using Kern's six-step approach in student-facilitated breakout sessions. Completed group projects were presented, including brief action plans for transforming their work into scholarship. The workshop was evaluated using a mixed-methods approach. **Results:** Of survey respondents, 44 mostly medical students, faculty, and administrators from different institutions rated the workshop as a very positive experience, and the pacing of the breakout groups as effective. A notable increase in self-reported mastery, as measured by learning objectives aligned with Kern's six-step model, was recorded from student respondents as compared to faculty. A sense of readiness to participate in curricular decisions either at the home institution or in individual career paths was evident from narrative comments. **Discussion:** Our workshop provided medical students with a foundation in curriculum development and educational scholarship. Session design provided flexibility in the pace of breakout sessions and allowed in-depth discussion of educational topics.

Keywords
Curriculum Development, Scholarship, Career Choice, Continuing Professional Development, Faculty Development

Educational Objectives

By the end of this activity, learners will be able to:

1. Apply principles of the generally accepted six-step approach for curriculum development for health professions education.
2. Describe characteristics of adult learners.
3. Develop specific, measurable, attainable, relevant, time-bound (SMART) learning objectives.
4. Describe educational strategies considered to promote active learning.
5. Identify factors influencing the implementation of health professions curricula.
6. Differentiate assessment and evaluation.
7. Describe foundations of educational scholarship.

Citation:
Schlegel EFM, Bird JB, Burns CM, et al. Curriculum design and scholarship for new educators: a professional development workshop for medical students. *MedEdPORTAL*. 2021;17:11130.
https://doi.org/10.15766/mep_2374-8265.11130

Introduction

Teaching is considered a physician's core skill, and the professional development of medical students has expanded to include the role of educator.[1] Competencies for clinician-educators include development and evaluation of medical curricula as well as their scholarly dissemination.[2-4] Clinician-educator tracks have advanced considerably over the last 2 decades and play a significant role in medical education workforce development. In addition, the proportion of residents who are receiving faculty appointments has been increasing steadily since at least 2015.[5-9]

Accrediting bodies and competency-based frameworks affirm educator skills as essential for medical trainees.[10,11] Recognizing

Copyright © 2021 Schlegel et al. This is an open-access publication distributed under the terms of the Creative Commons Attribution license.

this need, several medical schools offer workshops, formal student-as-teacher training programs, tracks, or education pathways.[1,12-14] However, regardless of the Liaison Committee on Medical Education graduation requirement of readiness "for the contemporary practice of medicine," comprehensive data confirming obligatory educator training in undergraduate medical education (UME) remain obscure, and most students graduate from medical school without formal skill training for curriculum development/evaluation and educational scholarship.[11,14-16]

Fostering research in the undergraduate curriculum instills skill sets and attitudes important to the pursuit of a medical career (e.g., an inquiring mind, clinical reasoning, and the capacity to engage in evidence-based practice[17]) thereby enabling students to engage in the global community of educational innovators for the benefit of medical students and teachers worldwide. Scholarship prompts the development of coherent and concise writing and recording as well as analytic and reasoning skills, contributing to developing lasting habits of critical thinking.[18] However, as outlined in the *Research in Medical Education Medical Students Primer,*[19] medical education scholarship affects entire educational systems and addresses contemporary questions in medical education—aiming to assess and reform the current medical educational culture as well as designing, evaluating, and supporting curricular innovations.[20] Furthermore, medical students are "front and center of the medical education process" and uniquely positioned to evaluate their own education.[19] Finally, early exposure to medical education research has been identified as a key indicator for considering a career in academic medicine.[21,22]

The importance of student-designed medical curricula leading to successful medical programs has not been recognized until recently,[23] and the role of medical students has been transformed from a passive consumer of education to an empowered stakeholder and curriculum developer during the last decade.[24,25] This rejuvenated approach to medical program development applies design thinking—including students' insights from previous learning experiences—for improvement of medical education.[26] Increasingly, students collaborate with medical teachers and design experts, share educational experiences, and implement curricular improvements, referred to as the cocreation of medical school content and curricula.[27] However, the catalyzing role of medical students in curricular change remains oftentimes unreported—indicating the need for medical students to acquire the foundation to transfer curricular development into scholarship.[27]

Despite increased awareness of the benefit of including students in leading curricular change, obstacles exist, and reports about such formal training remain sparse. In addition, a study on student leadership in curricular change conducted by the American Medical Association's Accelerating Change in Medical Education initiative revealed a "lack of formal leadership and medical education training" as one of many barriers to successful curricular change for students and faculty alike.[28]

To address this gap in medical student professional development and support the initiation of scholarship, we developed a 3-hour interactive workshop in partnership with the International Association of Medical Science Educators (IAMSE) Student Professional Development Committee. The workshop's primary aim was to provide novice learners without prior knowledge in curriculum design with an introductory, hands-on experience in applying the six-step model proposed by Kern at al.[28] to design a complete education activity with appropriate pedagogic strategies. The secondary aim was to explore models of converting curriculum design and development into educational scholarship.

To provide learner-centered active pedagogy, the workshop design was based on tenets of social constructivism, which offered the facilitation of challenging yet achievable tasks in groups using a worksheet with prompts aligned with Kern et al.'s six-step approach. Breakout group members combined previous experience and information provided in the workshop to develop a curriculum, while workshop facilitators circulated and provided guidance. Procedural scaffolding and sequenced discussion assisted in incorporating new knowledge for the educational product.[29]

While we found many scholarship guidance resources represented in *MedEdPORTAL*, we discovered only one resource (a webinar series) offering curriculum development training in combination with scholarship,[30] and only one course development workshop.[31] Both resources targeted either residents or faculty, required graduate medical education knowledge, and were less participant-driven and hands-on. Our workshop is unique in that it: (1) was customized for undergraduate medical students, relying solely on their educational experience and sense of health care needs for developing a curriculum; (2) successfully demonstrated facilitation in face-to-face and remote environments; and (3) can be presented in a standalone fashion or incorporated into a longer course focusing on foundations of medical education.

Copyright © 2021 Schlegel et al. This is an open-access publication distributed under the terms of the Creative Commons Attribution license.

Methods

Background

We designed a professional development preconference workshop for students attending the 2018 IAMSE annual conference, and student fees were paid by sponsorship. Another iteration of the workshop was conducted in 2019. All workshop facilitators were members of IAMSE with extensive experience in publishing, reviewing, and presenting educational innovations. A workshop agenda was created (Appendix A). To stimulate ideas for practicing Kern's curriculum development steps, we suggested seven contemporary topics, which allowed students to integrate previous learning experiences (Appendices B and C, Ideas for Your Educational Planning). The students' role and advocacy in curriculum development were discussed and a student-developed curriculum sample was presented.

Specifically, we asked student attendees to brainstorm and discuss their past and current involvement (if any) with medical curriculum design, and we encouraged them to reflect on how they could adapt what they learned to a specific course they had experienced. An interactive segment about translating educational innovation into scholarship and embracing a call to action inspired commitment. A detailed agenda (Appendix A), a slide deck (Appendix B), a worksheet with guiding prompts aligned with Kern's six-step model (Appendix C), and speaker notes (Appendix D) are included and can be modified for face-to-face or online delivery.

Conducting the Workshop

To facilitate the face-to-face workshop, we used rooms with screens, projectors, and round tables that accommodated four to six participants. Groups received flipcharts and felt pens for ease of presentation of results. Twenty-five participants attended the face-to-face workshops in 2018 and 2019.

With migration to a virtual milieu in 2020 due to public health concerns of the COVID-19 pandemic, we expanded our session to accommodate 64 participants using a web-based video teleconferencing platform (Zoom). Comparable to face-to-face seating, 10 breakout groups with six to eight participants in each were established and assigned to individual facilitators. In both workshop delivery formats, faculty facilitated large-group discussions for all participants. We divided participants into breakout rooms for each step of Kern's model, and each workshop facilitator circulated between breakout rooms to answer questions and monitor small-group progress. In both the face-to-face and remote settings, all participants remained in their teams for the duration of the workshop; a student leader was selected to represent the group when reporting out after completing each section of the Kern six-step model worksheet (Appendix C).

For the 2020 remote session, one host shared the screen with participants during each didactic segment, and the handout was saved as a Google Document. Ten numbered breakout rooms were established and aligned with a numbered Google Document. All participants received a generic Google Document, a link to the numbered Google Documents, and the combined slides. After an initial discussion of the importance of student leadership and involvement as stakeholders in curriculum development, participants were prompted to collaboratively develop a curriculum using the worksheet. The worksheet organized the activities according to Kern's six steps: (1) problem identification and general needs assessment; (2) targeted needs assessment; (3) goals and objectives; (4) educational strategies; (5) implementation; (6) evaluation and feedback.[28]

After reviewing Kern's steps 1 and 2, we provided highlights of adult learning theory.[32] After allocating time for groups to brainstorm topics, identify health care needs, and select the corresponding learner population, two groups were chosen to report out to the large group.

For facilitation of Kern's step 3, we deconstructed the components of specific, measurable, attainable, relevant, time-bound (SMART) learning objectives; explored and clarified the learning hierarchy inherent in each domain of Bloom's taxonomy;[33] and stressed the importance of alignment with instructional strategies and assessments. We asked participants to formulate only SMART learning objectives within the cognitive domain and invited two groups to discuss their goals and objectives with the larger group.

For facilitation of Kern's steps 4 and 5, we used different types of active pedagogy to explore and identify the resources and requirements necessary to implement a new curriculum.[34] Participants brainstormed educational strategies and identified resources needed for the planned curriculum; two groups reported out on their work.

Kern's step 6 included contrasting assessment and evaluation, clarifying summative and formative assessment, and using Kirkpatrick's model to evaluate medical education programs.[35] Participants designed assessment and evaluation systems for their curricula in their breakout groups, followed by a report-out session.

The transference of a medical education resource into scholarship combined group discussion with an individualized

Copyright © 2021 Schlegel et al. This is an open-access publication distributed under the terms of the Creative Commons Attribution license.

question-and-answer session. After reviewing key components of research including institutional review board approval,[20] we introduced medical education journal options and resources, such as *MedEdPORTAL*, *Medical Science Educator*, and the AAMC primer.[19] Finally, time was devoted to reflecting on how to transform curriculum into scholarship.

Mixed-Method Program Evaluation

The research project was approved by the Hofstra University Human Subjects Committee (approval reference number: 20200608-SOM-SCH-1).

We evaluated the 2020 remote workshop using quantitative and thematic analyses (Appendix E). We designed a postevent evaluation of satisfaction and session logistics. We also measured self-reported, perceived knowledge of individual learning objectives prior to and after the workshop through a retrospective pre/post 4-point Likert-style survey.[36] We incorporated three narrative prompts into the survey instrument:

1. What are the key messages/points you will take away from the workshop?
2. What other points would have you like to have covered?
3. Propose why knowledge of a formalized curriculum development process is important to your practice as an educator?

All evaluations were anonymous and collected after the workshop concluded. Feedback was used to modify and improve the workshop for the next iteration.

Descriptive statistics are presented as the percent of participants who responded to the top two Likert scale options for the logistics and satisfaction survey (percent of *agree* and *strongly agree*) and the perceived knowledge survey (percent of *knowledgeable* and *very knowledgeable*). Narrative comments were analyzed via a grounded theory approach to identify themes including key takeaways, additional points of interest, and impact of the newly acquired knowledge in the participants' practice as educators.

Results

The workshop was customized to medical students, with remaining seats made available to participants attending the IAMSE 2020 virtual conference. Of workshop attendees, 44 of 64 (71%) responded to the survey. Respondents were medical students from the United States (39%), or basic or clinical sciences teaching faculty (39%). Remaining respondents represented residents, medical school administration and leadership, and students of basic or medical sciences (Table).

Table. Number and Percent of Respondents by Status ($n = 44$)

Status	Number of Respondents	Percent of Respondents
First-year medical student	1	2
Second-year medical student	3	8
Third-year medical student	4	9
Fourth-year medical student	9	20
Resident	2	5
Basic sciences teaching faculty	16	36
Clinical sciences teaching faculty	1	2
Other	8	18

Experience

Most respondents agreed or strongly agreed that the workshop format was enjoyable (91%; Figure 1), the online format of the workshop worked well (95%), and was well organized (91%). The majority of respondents also agreed or strongly agreed that working in breakout groups (88%) and the pacing (79%) effectively facilitated learning in the workshop. Finally, almost all respondents agreed or strongly agreed that the workshop met their expectations (95%). There were no differences in respondent attitudes by status.

Self-reported Knowledge

The greatest increase in perceived knowledge among all respondents (students and faculty) was reported in developing a curriculum using Kern's six steps (Educational Objective 1), where the percent of respondents who reported being *knowledgeable* or *very knowledgeable* rose from 26% prior to the workshop to 87% after the workshop. There was also a substantial increase in respondents reporting to be *knowledgeable* or *very knowledgeable* in identifying factors influencing implementation (Educational Objective 5: 24% pre vs. 78% post). Respondents also reported an increase in: (1) perceived knowledge in describing characteristics of adult learners (Educational Objective 2: 51% pre vs. 82% post); (2) developing SMART learning objectives (Educational Objective 3: 51% pre vs. 92% post); (3) describing educational strategies to promote active learning (Educational Objective 4: 55% pre vs. 92% post); and (4) differentiating assessment and evaluation (Educational Objective 6: 50% pre vs. 86% post).

Specifically, medical student respondents reported the most perceived knowledge gains in developing a curriculum using Kern's six steps (Educational Objective 1: 18% pre vs. 80% post) and developing SMART learning objectives (Educational Objective 3: 29% pre vs. 87% post; Figure 2). Although faculty reported less perceived knowledge gains than medical students, they reported increases for all learning objectives, and in particular Educational Objective 1 (41% pre vs. 100% post) and Educational Objective 5 (29% pre vs. 79% post; Figure 2).

Copyright © 2021 Schlegel et al. This is an open-access publication distributed under the terms of the Creative Commons Attribution license.

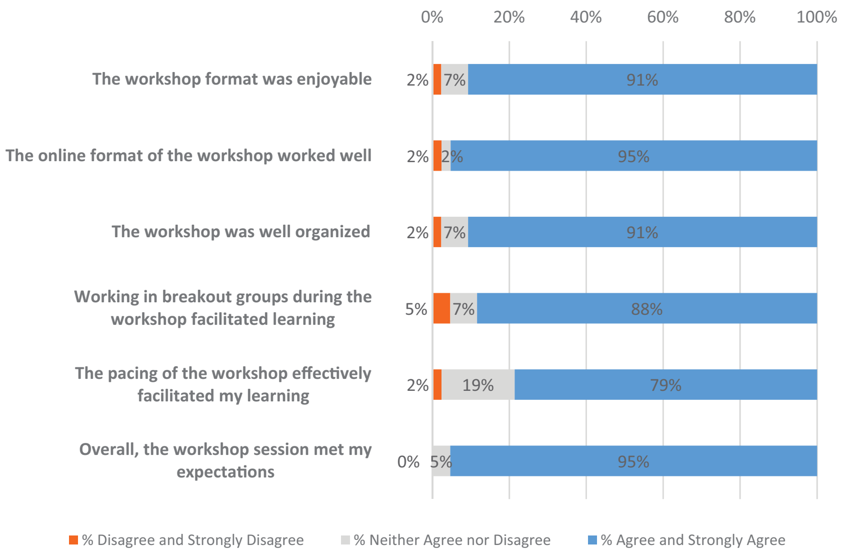

Figure 1. Respondent attitudes toward the quality of the workshop ($n = 44$).

Narrative Feedback

Analyses from the first prompt identified the following themes: (1) planning and designing a well thought out curriculum, (2) preparing a hypothesis for research, (3) connecting curriculum development with scholarship in a feasible manner, (4) being provided with a feasible plan to getting published, and (5) mastering the development of an online workshop. No difference between students and other respondents was noted.

Analysis of the second prompt revealed that all respondents expressed interest in additional in-depth knowledge on personalized curriculum examples from the host educators. It was noted that students desired more information on the transfer into scholarship. Participants at more advanced career levels expressed further discussion on applying learning theories and teaching techniques, assessment, and use of software applications.

Analysis of the third prompt revealed that respondents collectively expressed inspiration for positive changes, from individual sessions and “lectures/labs/simulations/teaching videos, etc.” to the “academic practice.” Respondents appreciated being informed about curriculum development and felt empowered to make personal career decisions and contribute to their institution. In addition, planning and organizing knowledge informed ideas for different educational roles, such as faculty development or scholarly work. A recently graduated respondent mentioned that the introduction to medical education needs to start early to, “Empower learners to become teachers and inspire positive change.”

Discussion

We conducted a 3-hour, interactive, remotely delivered workshop customized for undergraduate medical students (without prerequisite knowledge in curricular design or medical education research) that combined an introduction to Kern’s model of curricular design with multiple breakout sessions focused on knowledge application and the transformation of curricula into scholarship.[28] The goal was to enable participants to develop a structured medical curriculum using solely UME experience and achieve an understanding of its transfer to scholarship. Workshop faculty encouraged students to identify and complete curricular topics and assume leadership roles in the breakout sessions. Based on the outcomes and evaluation data, we believe our workshop’s design and implementation are feasible, adaptable (especially to the dynamic conditions imposed with the emergence of the COVID-19 pandemic), and effective whether implemented using face-to-face or remote modalities.

The workshop aimed primarily to provide novice medical learners without competencies in medical education to: (1) develop their proficiency in engaging in educational activities and scholarship, and (2) expand their academic career options.[2-4] Our approach was effective, evidenced by the student respondents’ report of enhanced perceived knowledge as compared to attending faculty. During the workshop students developed programs with urgent and highly relevant topics such as health and racial

Copyright © 2021 Schlegel et al. This is an open-access publication distributed under the terms of the Creative Commons Attribution license.

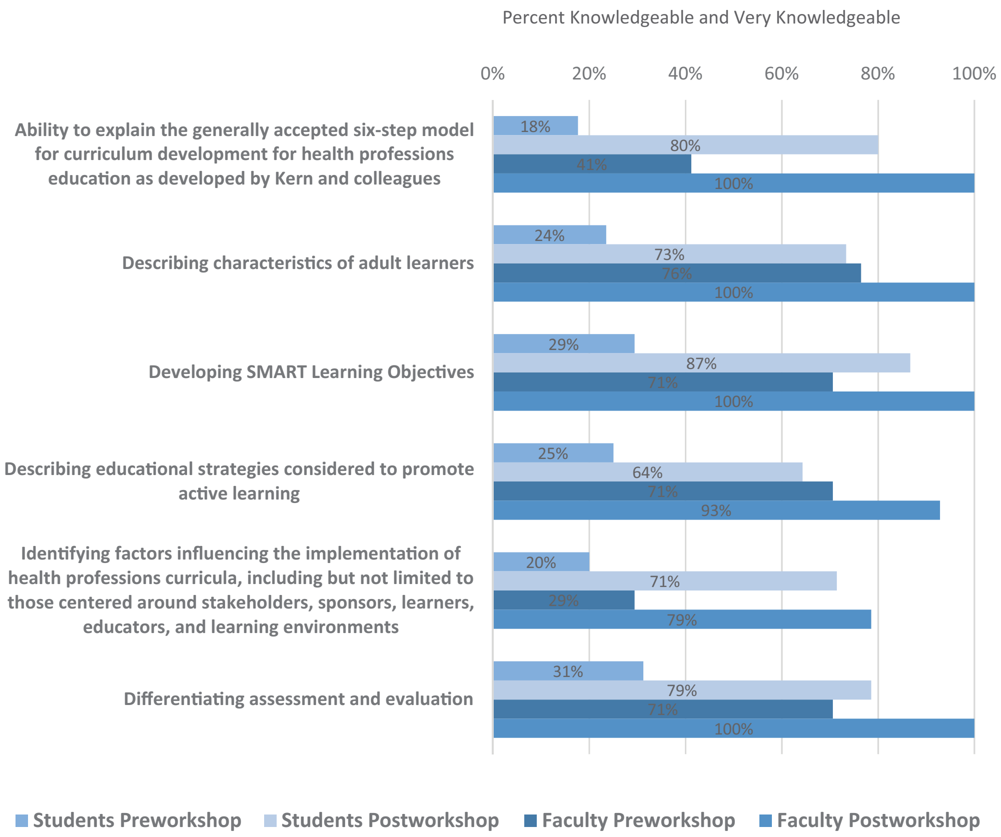

Figure 2. A comparison of clinical and basic science teaching faculty ($n = 17$) and medical students years 1-4 ($n = 17$) perceived knowledge of learning objectives pre- and postworkshop.

disparities, bias in medicine, and burnout in interns. Overall, this action-oriented strategy of the workshop empowered and stimulated students to develop curricula and assume ownership and stakeholder roles in the process, enabling them to navigate opportunities to cocreate teaching and learning.[24,25]

To improve instruction in future iterations, we used a mixed-method approach to evaluate the workshop. Our framework evaluated New World Kirkpatrick's Model (NWKM) level 1 (learner reaction) using a traditional survey, and NWKM level 2 (learning expressed as confidence to accomplish), applying a retrospective pre/postassessment.[35,37] Narrative feedback was elicited through prompts that supported the quantitative questions. The retrospective pre/postassessment evaluated the perceived accomplishment of learning objectives aligned with the course objectives in one step, eliminating the need to administer two separate surveys.[36] Additionally, the survey could remain anonymous.

As reflected by the quantitative feedback from students, the greatest increase in perceived knowledge was reported in developing a curricular framework using Kern's six steps, followed by feeling well informed about steps for implementing a curriculum. The lowest increase in perceived knowledge was reflected in differentiating assessment and evaluation, and in developing SMART learning objectives, indicating that learners were exposed to these significant teaching and learning principles during their undergraduate education.

Participants reported that the remotely delivered workshop was a very positive experience. The use of breakout rooms in the remote session was reported as effective, but both pacing and breakout group collaboration received slightly less favorable feedback. At both iterations in 2019 and for online delivery in 2020 the workshop was realigned with the objectives in content, timeframe, and learning activities. In addition, based on standard workshop surveys conducted by the organizer in 2018 and 2019, workshop pacing has been addressed by flexibility in delivery of the didactic sections and increasing the time for breakout sessions. We now intend to provide question-driven guidance to future student groups by workshop faculty. Although the

Copyright © 2021 Schlegel et al. This is an open-access publication distributed under the terms of the Creative Commons Attribution license.

workshop can be adjusted to accommodate different numbers of participants, resulting in different numbers of breakout groups, smaller numbers of participants allow a more robust discussion, thereby providing time to discuss examples.

Since the launch of the first workshop in 2018, we added a personalized question-and-answer session with faculty at the end of the workshop. Nevertheless, students expressed a strong desire for additional information on scholarly work, which will be addressed by introducing scholarship prior to the curriculum design step-by-step approach. In addition, the workshop can be modified to add pertinent examples and case studies of published educational work. As an additional outcome, faculty participants indicated interest in in-depth discussion of learning theory and narrative assessments relevant for faculty-directed workshops.

There were several limitations to this work. First, the focus of measurement was limited to the reactions and self-reports of participants, corresponding to NWKM levels 1 and 2.[37] Thus, transfer of skills to another educational environment and the outcome of such intervention within this environment were undetermined (NWKM levels 3 and 4). Second, this mixed-method framework could benefit from additional in-depth qualitative research, such as focus groups, to learn more about the needs of participants and the breakout group dynamics. Thus, further studies are needed to refine the workshop framework and content.

Important lessons learned included flexibility in pacing to accommodate participants' workflow and mindfulness of the number of participants, which determined the depth of discussion. For remote delivery, workshop preparation and reliable technological support were key. The number of participants will affect the logistics for remote delivery. For preparation of the remote workshop, it was necessary to determine the number of breakout rooms, a master Google Document in Appendix C (worksheet), and copies of it for each breakout group. Course documents (Appendices B and C) should be ready for upload in the chat as backup. The challenge of pacing the workshop can be mitigated through communication among host faculty and tech support (e.g., via cell phone texting), determining roles among the facilitators (e.g., timekeeper, chat monitor, etc.), and splitting up the breakout groups for individualized facilitation. Finally, all facilitators should (ideally) be able to present the entire session and function as backup for each other if web connectivity fails.

In summary, this workshop and corresponding appendices addressed an important two-fold gap in medical student professional development: educator training in curriculum development and initiation of medical education scholarship. The highly interactive workshop design was based on social constructivism, was adaptable to face-to-face and remote environments, offered a hands-on experience in applying Kern's six-step approach, and provided insight into appropriate pedagogic strategies. The effectiveness of the intervention was evaluated using a mixed-methods framework, which revealed opportunities for improvement as a learning experience to inspire positive change.

Appendices

A. Workshop Agenda.docx
B. Presentation.pptx
C. Worksheet.docx
D. Facilitator Notes.docx
E. Workshop Survey.docx

All appendices are peer reviewed as integral parts of the Original Publication.

Elisabeth F.M. Schlegel, MSc, PhD, MBA, MS (HPPL): Associate Professor of Science Education and Assistant Director of Faculty Development and Medical Education Research, Donald and Barbara Zucker School of Medicine at Hofstra/Northwell; ORCID: https://orcid.org/0000-0002-0279-1438

Jeffrey B. Bird, MA: Assistant Professor of Science Education and Educational Research and Strategic Assessment Analyst, Donald and Barbara Zucker School of Medicine at Hofstra/Northwell; ORCID: https://orcid.org/0000-0002-7651-0432

Christopher M. Burns, PhD: Professor and Chair of Biomedical Education, College of Osteopathic Medicine, California Health Sciences University

Michael Cassara, DO, MSEd: Associate Professor of Emergency Medicine, Donald and Barbara Zucker School of Medicine at Hofstra/Northwell; Associate Professor of Nursing, Hofstra/Northwell School of Nursing and Physician Assistant Studies; Medical Director, Northwell Health Patient Safety Institute/Emergency Medical Institute; ORCID: https://orcid.org/0000-0001-6483-4838

Jessica O'Neil, MD: Resident, Department of Medicine, Massachusetts General Hospital

Yun Weisholtz, MD, PhD: Associate Director of Student Affairs, ScholarRx

Tao T. Le, MD, MHS: Associate Clinical Professor Medicine and Pediatrics, and Chief of Section of Allergy and Immunology, Department of Medicine, University of Louisville School of Medicine

Copyright © 2021 Schlegel et al. This is an open-access publication distributed under the terms of the Creative Commons Attribution license.

Acknowledgments
The authors are very grateful for the support provided by the International Association of Medical Science Educators under the leadership of Neil Osheroff, president; Bonny Dickinson, 2020 program committee chair; and especially Danielle Inscoe, managing director. Ms. Inscoe provided invaluable professional support with the virtual teaching environment that made the session run smoothly. Many thanks also to Alice Fornari, professor of science education at the Zucker School of Medicine at Hofstra/Northwell and vice president of faculty development, Northwell Health, for her contributions in developing the initial worksheet and the overall concept. The authors also appreciate and acknowledge the expert librarian support by Wendy Herman, Zucker School of Medicine at Hofstra/Northwell; valuable advice from Doreen Olvet, medical education research project manager; and knowledgeable editing by Linda Merklin.

Disclosures
None to report.

Funding/Support
None to report.

Ethical Approval
The Hofstra University Human Subjects Committee approved this study.

References

1. Erlich DR, Shaughnessy AF. Student-teacher education programme (STEP) by step: transforming medical students into competent, confident teachers. *Med Teach.* 2014;36(4):322-332. https://doi.org/10.3109/0142159X.2014.887835
2. Marton GE, McCullough B, Ramnanan CJ. A review of teaching skills development programmes for medical students. *Med Educ.* 2015;49(2):149-160. https://doi.org/10.1111/medu.12571
3. Sherbino J, Frank JR, Snell L. Defining the key roles and competencies of the clinician-educator of the 21st century: a national mixed-methods study. *Acad Med.* 2014;89(5):783-789. https://doi.org/10.1097/ACM.0000000000000217
4. Harden RM, Laidlaw JM. *Essential Skills for a Medical Teacher: An Introduction to Teaching and Learning in Medicine.* Elsevier; 2020.
5. *Table C8: Full-Time Faculty Appointment Status at US Medical Schools for Completed Residents, by Specialty and Rank.* Association of American Medical Colleges; 2015.
6. *Table C8: Full-Time Faculty-Appointment Status at US Medical Schools for Residents Who Completed Residencies, by Specialty and Rank.* Association of American Medical Colleges; December 2016.
7. *Table C8. Full-Time Faculty-Appointment Status at US Medical Schools for Residents Who Completed Residencies, by Specialty and Rank.* Association of American Medical Colleges; 2017.
8. *Table C8: Full-Time Faculty-Appointment Status at US Medical Schools for Residents Who Completed Residencies, by Specialty and Rank.* Association of American Medical Colleges; 2018.
9. *Table C8. Full-Time Faculty-Appointment Status at US Medical Schools for Residents Who Completed Residencies, by Specialty and Rank.* Association of American Medical Colleges; 2019.
10. *ACGME Common Program Requirements (Residency).* Accreditation Council for Graduate Medical Education; 2020. Accessed March 6, 2021. https://www.acgme.org/Portals/0/PFAssets/ProgramRequirements/CPRResidency2020.pdf
11. Liaison Committee on Medical Education. *Functions and Structure of a Medical School.* Association of American Medical Colleges; 2020. https://lcme.org/wp-content/uploads/filebase/standards/2021-22_Functions-and-Structure_2020-11-2.docx
12. Soriano RP, Blatt B, Coplit L, et al. Teaching medical students how to teach: a national survey of students-as-teachers programs in US medical schools. *Acad Med.* 2010;85(11):1725-1731. https://doi.org/10.1097/ACM.0b013e3181f53273
13. Blanco MA, Maderer A, Oriel A, Epstein SK. How we launched a developmental student-as-teacher (SAT) program for all medical students. *Med Teach.* 2014;36(5):385-389. https://doi.org/10.3109/0142159X.2014.886770
14. Chen HC, Wamsley MA, Azzam A, Julian K, Irby DM, O'Sullivan PS. The health professions education pathway: preparing students, residents, and fellows to become future educators. *Teach Learn Med.* 2017;29(2):216-227. https://doi.org/10.1080/10401334.2016.1230500
15. Zijdenbos I, Fick T, ten Cate O. How we offer all medical students training in basic teaching skills. *Med Teach.* 2011;33(1):24-26. https://doi.org/10.3109/0142159X.2011.530309
16. Burgess A, McGregor D. Peer teacher training for health professional students: a systematic review of formal programs. *BMC Med Educ.* 2018;18(1):263. https://doi.org/10.1186/s12909-018-1356-2
17. Laidlaw A, Aiton J, Struthers J, Guild S. Developing research skills in medical students: AMEE guide no. 69. *Med Teach.* 2012;34(9):e754-e771. https://doi.org/10.3109/0142159X.2012.704438
18. Bennett C. Why all medical students need to experience research. *Aust Med Stud J.* 2016;7(1):10-11.
19. Atluru A, Wadhwani A, Maurer K, et al. *Research in Medical Education: A Primer for Medical Students*. Association of American Medical Colleges; 2015.
20. Glassick CE, Huber MT, Maeroff GI. *Scholarship Assessed: Evaluation of the Professoriate.* Jossey-Bass; 1997.
21. O'Sullivan PS, Niehaus B, Lockspeiser TM, Irby DM. Becoming an academic doctor: perceptions of scholarly careers. *Med Educ.* 2009;43(4):335-341. https://doi.org/10.1111/j.1365-2923.2008.03270.x
22. Williams R, Holaday L, Lamba S, Soto-Greene M, Sanchez JP. Introducing trainees to medical education activities and

Copyright © 2021 Schlegel et al. This is an open-access publication distributed under the terms of the Creative Commons Attribution license.

opportunities for educational scholarship. *MedEdPORTAL*. 2017;13:10554. https://doi.org/10.15766/mep_2374-8265.10554

23. Peluso M, Takizawa P. Student involvement in the development of integrated curricula. *Med Educ.* 2010;44(11):1120-1121. https://doi.org/10.1111/j.1365-2923.2010.03840.x
24. Geraghty JR, Young AN, Berkel TDM, et al. Empowering medical students as agents of curricular change: a value-added approach to student engagement in medical education. *Perspect Med Educ.* 2020;9(1):60-65. https://doi.org/10.1007/s40037-019-00547-2
25. Könings KD, Mordang S, Smeenk F, Stassen L, Ramani S. Learner involvement in the co-creation of teaching and learning: AMEE guide no. 138. *Med Teach*. Published online November 6, 2020. https://doi.org/10.1080/0142159X.2020.1838464
26. Badwan B, Bothara R, Latijnhouwers M, Smithies A, Sandars J. The importance of design thinking in medical education. *Med Teach.* 2018;40(4):425-426. https://doi.org/10.1080/0142159X.2017.1399203
27. Burk-Rafel J, Harris KB, Heath J, Milliron A, Savage DJ, Skochelak SE. Students as catalysts for curricular innovation: a change management framework. *Med Teach.* 2020;42(5):572-577. https://doi.org/10.1080/0142159X.2020.1718070
28. Thomas PA, Kern DE, Hughes MT, Chen BY, eds. *Curriculum Development for Medical Education: A Six-Step Approach.* 3rd ed. Johns Hopkins University Press; 2016.
29. Taylor DCM, Hamdy H. Adult learning theories: implications for learning and teaching in medical education: AMEE guide no. 83. *Med Teach.* 2013;35(11):e1561-e1572. https://doi.org/10.3109/0142159X.2013.828153
30. Martin SK, Ahn J, Farnan JM, Fromme HB. Introduction to curriculum development and medical education scholarship for resident trainees: a webinar series. *MedEdPORTAL.* 2016;12:10454. https://doi.org/10.15766/mep_2374-8265.10454
31. Hartke A, Devon EP, Burns R, Rideout M. Building a boot camp: pediatric residency preparatory course design workshop and tool kit. *MedEdPORTAL.* 2019;15:10860. https://doi.org/10.15766/mep_2374-8265.10860
32. Knowles MS. *The Adult Learner: A Neglected Species.* Gulf Publishing Co; 1990.
33. Anderson LW, Krathwohl DR, eds. *A Taxonomy for Learning, Teaching, and Assessing: A Revision of Bloom's Taxonomy of Educational Objectives.* Pearson; 2000.
34. Bonwell CC, Eison JA. *Active Learning: Creating Excitement in the Classroom.* Jossey-Bass; 1991.
35. Kirkpatrick DL, Kirkpatrick JD. *Evaluating Training Programs: The Four Levels.* 3rd ed. Berrett-Koehler; 2006.
36. Bhanji F, Gottesman R, de Grave W, Steinert Y, Winer LR. The retrospective pre-post: a practical method to evaluate learning from an educational program. *Acad Emerg Med.* 2012;19(2):189-194. https://doi.org/10.1111/j.1553-2712.2011.01270.x
37. Kirkpatrick J, Kirkpatrick W. *An Introduction to the New World Kirkpatrick Model*. Kirkpatrick Partners; 2019.

Received: August 27, 2020
Accepted: January 14, 2021
Published: April 26, 2021

Copyright © 2021 Schlegel et al. This is an open-access publication distributed under the terms of the Creative Commons Attribution license.
